# Targeting BRCA1 and BRCA2 Deficiencies with G-Quadruplex-Interacting Compounds

**DOI:** 10.1016/j.molcel.2015.12.004

**Published:** 2016-02-04

**Authors:** Jutta Zimmer, Eliana M.C. Tacconi, Cecilia Folio, Sophie Badie, Manuela Porru, Kerstin Klare, Manuela Tumiati, Enni Markkanen, Swagata Halder, Anderson Ryan, Stephen P. Jackson, Kristijan Ramadan, Sergey G. Kuznetsov, Annamaria Biroccio, Julian E. Sale, Madalena Tarsounas

**Affiliations:** 1 Genome Stability and Tumourigenesis Group, CRUK/MRC Oxford Institute for Radiation Oncology, Department of Oncology, University of Oxford, Old Road Campus Research Building, Oxford OX3 7DQ, UK; 2 Area of Translational Research, Regina Elena National Cancer Institute, 00144 Rome, Italy; 3 Institute for Molecular Medicine Finland (FIMM), University of Helsinki, P.O. Box 20, FIN-00014 Helsinki, Finland; 4 Biochemistry and Regulation of DNA Repair Group, CRUK/MRC Oxford Institute for Radiation Oncology, Department of Oncology, University of Oxford, Old Road Campus Research Building, Oxford OX3 7DQ, UK; 5 DNA Damage and Repair Group, CRUK/MRC Oxford Institute for Radiation Oncology, Department of Oncology, University of Oxford, Old Road Campus Research Building, Oxford OX3 7DQ, UK; 6 Lung Cancer Translational Science Research Group, CRUK/MRC Oxford Institute for Radiation Oncology, Department of Oncology, University of Oxford, Old Road Campus Research Building, Oxford OX3 7DQ, UK; 7 The Gurdon Institute, CRUK Laboratories, University of Cambridge, Tennis Court Road, Cambridge CB2 1QN, UK; 8 The Sanger Institute, Hinxton, Cambridge CB10 1SA, UK; 9 Medical Research Council Laboratory of Molecular Biology, Francis Crick Avenue, Cambridge CB2 0QH, UK

## Abstract

G-quadruplex (G4)-forming genomic sequences, including telomeres, represent natural replication fork barriers. Stalled replication forks can be stabilized and restarted by homologous recombination (HR), which also repairs DNA double-strand breaks (DSBs) arising at collapsed forks. We have previously shown that HR facilitates telomere replication. Here, we demonstrate that the replication efficiency of guanine-rich (G-rich) telomeric repeats is decreased significantly in cells lacking HR. Treatment with the G4-stabilizing compound pyridostatin (PDS) increases telomere fragility in BRCA2-deficient cells, suggesting that G4 formation drives telomere instability. Remarkably, PDS reduces proliferation of HR-defective cells by inducing DSB accumulation, checkpoint activation, and deregulated G2/M progression and by enhancing the replication defect intrinsic to HR deficiency. PDS toxicity extends to HR-defective cells that have acquired olaparib resistance through loss of 53BP1 or REV7. Altogether, these results highlight the therapeutic potential of G4-stabilizing drugs to selectively eliminate HR-compromised cells and tumors, including those resistant to PARP inhibition.

## Highlights

• G4 formation on the G-rich strand drives telomere fragility in HR-deficient cells
• G4-stabilizing compounds reduce viability of cells lacking BRCA1, BRCA2, or RAD51
• G4 toxicity stems from excessive replication stress and DNA damage accumulation
• Olaparib-resistant, BRCA-defective cells are sensitive to G4-stabilizing compounds

## Graphical Abstract

**Figure:**
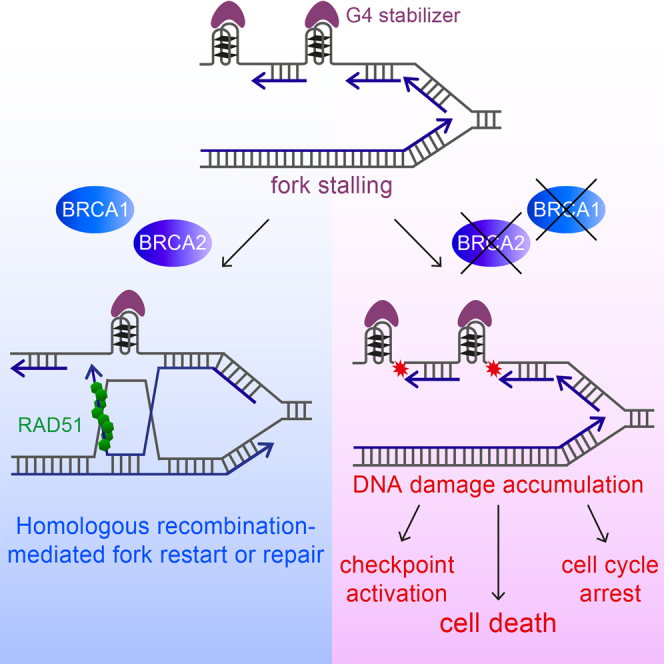

## Teaser

Zimmer et al. discovered that homologous recombination activities of BRCA1 and BRCA2 facilitate replication of genomic regions with G-quadruplex-forming potential, including telomeres, and suppress genomic instability stemming from inefficient replication of these sites. G4-stabilizing compounds are toxic to BRCA1- and BRCA2-deficient cells, highlighting their therapeutic potential in targeting BRCA deficiency.

## Introduction

Genomic instability is a hallmark of cancer caused by failure of normal DNA replication and/or repair mechanisms (Halazonetis et al., 2008, Negrini et al., 2010). During replication, the enzymatic activities of DNA polymerases, helicases, and nucleases act in concert to assemble the active replication fork and to achieve high-fidelity duplication of the genome. Damaged DNA, secondary DNA structures, and DNA-protein complexes obstruct progression of replication forks, leading to fork stalling or, in more severe cases, to irreversible fork collapse and DNA breakage. Several mechanisms have evolved to overcome barriers to replication-fork movement, one of which exploits the HR DNA repair machinery. HR factors act to stabilize stalled replication forks by preventing their nucleolytic degradation (Hashimoto et al., 2010, Schlacher et al., 2011) to restart arrested forks (Lambert et al., 2010) and to repair double-strand breaks (DSBs) arising from disintegrated forks (Aze et al., 2013).

The tumor suppressor BRCA2 is a key component of the HR pathway of DSB repair. BRCA2 promotes recombination reactions by loading the RAD51 recombinase onto single-stranded DNA (ssDNA) in concert with the family of proteins known as the RAD51 paralogs, of which RAD51C is a member (Suwaki et al., 2011). RAD51-coated ssDNA invades an intact, homologous duplex DNA molecule, most commonly a sister chromatid, which becomes the template for accurate DSB repair.

In vitro, G-rich ssDNA can adopt secondary structures known as G4s under physiological-like conditions (Lipps and Rhodes, 2009). G4s consist of stacks of two or more G-quartets formed by four guanines via Hoogsteen base pairing stabilized by a monovalent cation. While in silico analyses have identified more than 300,000 sites with G4-forming potential in the human genome (Huppert and Balasubramanian, 2005), more recent G4-seq approaches enabled detection of more than 700,000 G4 structures genome-wide (Chambers et al., 2015). The first in vitro visualization of a G4 structure was based on diffraction patterns of a guanylic acid solution (Gellert et al., 1962), while evidence that G4s assemble in vivo initially came from immunostaining of *Stylonychia* macronuclei with antibodies raised against G4 structures with telomere sequences (Schaffitzel et al., 2001). This study demonstrated that telomeres adopt a G4 configuration in vivo. G4 structures have been subsequently detected with several other structure-specific antibodies (Biffi et al., 2013, Henderson et al., 2014, Schaffitzel et al., 2001) and interacting small molecules (Lam et al., 2013, Müller et al., 2010, Rodriguez et al., 2012). Importantly, telomeric G-rich DNA sequences have a high propensity to adopt G4 configurations (Parkinson et al., 2002). Telomeres, repetitive DNA sequences bound by the protein complex shelterin, protect chromosome ends from degradation and fusion. Telomeric G4s can interfere with telomere replication, leading to fragile, shorter telomeres. Supporting this concept, treatment with G4-stabilizing compounds induces telomere dysfunction (Gomez et al., 2006, Rodriguez et al., 2008, Salvati et al., 2007, Tahara et al., 2006).

During DNA replication, G4s are thought to assemble spontaneously on G-rich ssDNA displaced during fork movement. Due to their thermodynamic stability, G4s cause uncoupling of replisome components and fork stalling, which have the potential to trigger genomic instability. Helicases such as FANCJ, PIF1, RECQ, BLM, and WRN, the chromatin remodeler ATRX, and the REV1 translesion polymerase act to dismantle G4s in vitro. Several lines of evidence support a similar function in vivo for these factors, essential to preserve genome stability during DNA replication (Murat and Balasubramanian, 2014). Conversely, G4 configurations can be stabilized by specific ligands that exhibit higher binding specificity for G4s over duplex DNA, with the G4-interacting compound PDS being one example (Chambers et al., 2015). In mammalian cells, G4 stabilization by PDS results in dissociation of shelterin components from telomeres (Rodriguez et al., 2008). More recently, PDS was demonstrated to trigger replication- and transcription-associated DNA damage at genomic sites with predicted G4-forming potential (Lam et al., 2013, Rodriguez et al., 2012). These findings highlight the deleterious consequences of persistent G4s for telomere and genome integrity.

HR factors, including BRCA2 and RAD51, are required to facilitate telomere replication and to prevent telomere shortening (Badie et al., 2010). It remained unclear, however, whether assembly of telomeric G4s could contribute to the telomere replication defect of HR-deficient cells. In this work, we demonstrate that telomere fragility in cells lacking HR repair is enhanced by PDS treatment. Importantly, G4-stabilizing compounds, including PDS, decrease the viability of BRCA1-, BRCA2-, or RAD51-deficient cells, which is associated with elevated levels of DNA damage and replication stress. We suggest that in the context of HR deficiency, persistent G4 structures exacerbate the cell-intrinsic challenges that arise during replication of regions with G4-forming potential, thus eliciting checkpoint activation, G2/M cell-cycle arrest, and cell death. This work is therefore highly relevant to the search for treatments that selectively kill tumor cells whose capacity for HR-mediated repair has been compromised.

## Results

### BRCA2 and RAD51C Are Required for G-Rich Strand Telomere Replication

**Figure 1:**
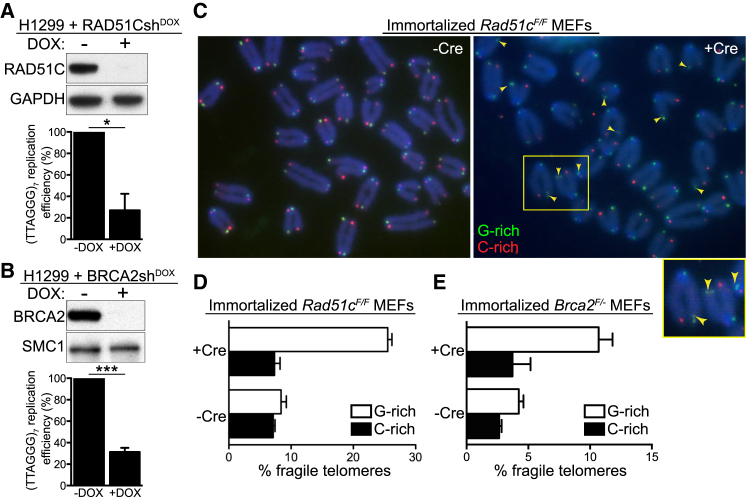
RAD51C and BRCA2 Prevent Lagging-Strand Telomere Fragility (A and B) Replication efficiency of a plasmid containing (TTAGGG)_7_ in H1299 cells expressing doxycycline (DOX)-inducible RAD51C (A) or BRCA2 (B) shRNAs is shown relative to the replication efficiency of the empty vector (n = 3 for RAD51Csh^DOX^; n = 4 for BRCA2sh^DOX^; error bars, SEM). p values were calculated using a one-sample t test (^∗^p ≤ 0.05 and ^∗∗∗^p ≤ 0.001). Cell extracts prepared at the time of plasmid transfection were immunoblotted as indicated. GAPDH and SMC1 were used as loading controls. (C) CO-FISH detection of lagging (G-rich, green) and leading (C-rich, red) telomeric strands in immortalized *Rad51c*^*F/F*^ MEFs treated with Cre (+Cre) and control (−Cre) retroviruses. Enlarged inset shows the area marked with the yellow rectangle. Arrows mark lagging-strand fragile telomeres. (D and E) Quantification of fragile telomeres in immortalized *Rad51c*^*F/F*^ (D) and *Brca2*^*F/-*^ (E) MEFs. Approximately 1,000 telomeres were scored per condition per replica (n = 2; error bars, SD). See also Figure S1.

Abrogation of key HR activities elicits telomere fragility (Badie et al., 2010) suggestive of a role for HR in telomere replication. To further investigate this concept, we used a plasmid-based replication assay (Szüts et al., 2008) in H1299 cells harboring inducible small hairpin RNA (shRNA) against RAD51C or BRCA2. Doxycycline addition induced efficient depletion of both proteins, as determined by western blotting (Figures 1A and 1B). The replication efficiency of a plasmid containing an array of seven telomeric repeats (TTAGGG)_7_ was significantly lower in RAD51C- or BRCA2-deficient cells compared to control cells (Figures 1A and 1B). RAD51C inhibition did not affect cell proliferation rate (Figure S1A, available online). Full-length human RAD51C rescued the telomere replication defect completely, indicating specificity of the shRNA for its target (Figure S1B). Importantly, replication of a plasmid containing a (TTACGC)_7_ sequence, with two G-to-C substitutions in the telomere repeat, which abrogate the G4-forming potential of the sequence, was not affected by loss of RAD51C expression (Figure S1C). Collectively, these data suggest that assembly of G4 secondary structures on the telomere-containing plasmid underlines its inefficient replication in BRCA2- or RAD51C-depleted cells.

We previously reported that *Brca2* or *Rad51c* deletion in mouse embryonic fibroblasts (MEFs) leads to increased levels of multiple telomeric fluorescence in situ hybridization (FISH) signals (Badie et al., 2010), indicative of telomere fragility. To examine the specificity of the fragile telomere phenotype to the leading or lagging-strand template, chromosome orientation FISH (CO-FISH) assays were performed in immortalized *Brca2*^*F/-*^ or *Rad51c*^*F/F*^ MEFs, in which gene deletion was induced with “hit-and-run” Cre recombinase. The telomeric G-rich strand showed a clear propensity for FISH signal fragmentation (Figure 1C, green). Quantification of fragmented telomeric CO-FISH signals further demonstrated the bias toward fragility of the G-rich telomeric strand in Cre-treated *Brca2*^*F/-*^ and *Rad51c*^*F/F*^ MEFs (Figures 1D and 1E) as well as in a *Brca2*^*−/−*^ mouse mammary tumor-derived cell line (Evers et al., 2010; Figure S1D).

### G4 Structure Stabilization Exacerbates the Telomere Fragility in *Brca2*-Deleted MEFs

**Figure 2:**
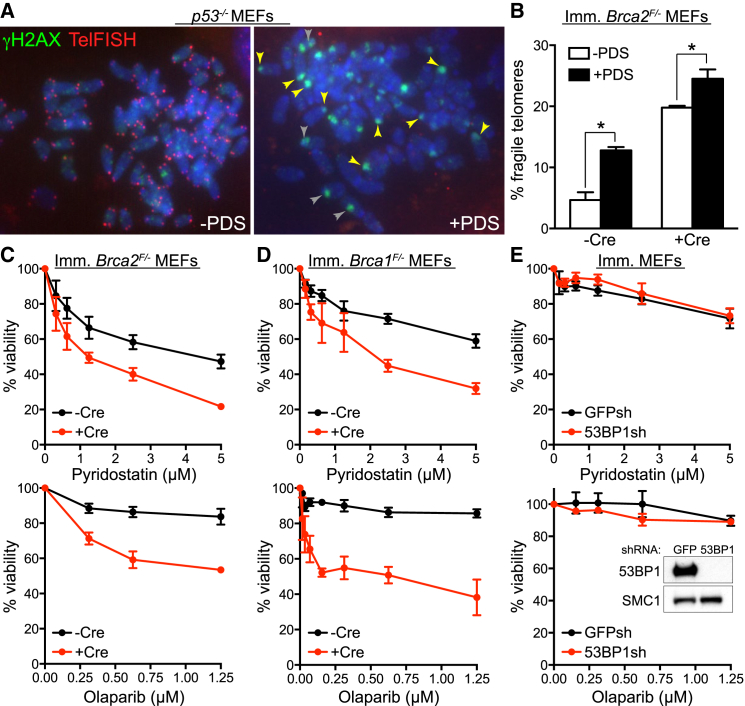
Effect of the G4-Interacting Compound PDS on Telomere Fragility and Viability of *Brca*-Deficient MEFs (A) Mitotic chromosome spreads of *p53*^*−/−*^ MEFs grown in the presence (+PDS) or absence (−PDS) of 5 μM PDS for 48 hr. Preparations were fixed and stained with anti-γH2AX monoclonal antibody (green). Telomeres were visualized with a Cy3-conjugated (CCCTAA)_6_-PNA probe (red), using identical exposure conditions for untreated and PDS-treated cells. DNA was counterstained with DAPI (blue). (B) Quantification of fragile telomeres visualized by FISH on metaphase chromosomes from *Brca2*^*F/-*^ MEFs treated with Cre (+Cre) and control (−Cre) retroviruses incubated with 5 μM PDS for 40 hr (n = 2; > 1,500 long-arm telomeres were scored per condition per replica; error bars, SD). p values were calculated using an unpaired two-tailed t test (^∗^p ≤ 0.05). (C) Dose-dependent viability assays of *Brca2*^*F/-*^ MEFs treated with Cre (+Cre) and control (−Cre) retroviruses exposed to PDS or olaparib at the indicated concentrations. (D) Dose-dependent viability assays of *Brca1*^F/-^ MEFs treated as in (C). (E) Dose-dependent viability assays of immortalized (imm.) MEFs treated as in (C) with retroviruses encoding shRNA against GFP or 53BP1 (Bouwman et al., 2010). Cell extracts were immunoblotted as indicated. SMC1 was used as a loading control. See also Figures S1 and S2. Graphs shown are representative of at least two independent experiments, each performed in triplicate. Error bars represent SD of triplicate values obtained from a single experiment.

Telomere fragility indicates a telomere replication defect (Martínez et al., 2009, Sfeir et al., 2009), which is thought to stem from the potential of telomere DNA sequences to adopt G4 secondary structures known to obstruct replication fork progression. To test whether telomere fragility in HR-deficient cells was linked to G4 formation, we used the G4 ligand PDS (Rodriguez et al., 2008, Rodriguez et al., 2012) to treat *p53*^*−/−*^ MEFs, which are known to proliferate in the presence of DNA damage, followed by immunofluorescence combined with telomere FISH (IF-FISH) detection. Exposure to PDS led to accumulation of nuclear foci of the Ser139-phosphorylated form of histone H2AX (γH2AX, Figure 2A), a well-established DSB marker. A subset of these foci colocalized with chromosome ends (Figure 2A, yellow arrowheads), while others localized intrachromosomally (Figure 2A, gray arrowheads). A similar PDS effect has been reported in human cells (Rodriguez et al., 2012). In addition, PDS triggered a dramatic reduction in the intensity of telomere FISH signals corresponding to the G-rich telomere strand (Figures 2A and S1E). In these images, the same exposure time was used for image acquisition of untreated and PDS-treated cells, to enable comparison of the telomeric signal intensity between the two samples. In contrast, in Figure 2B the exposure time was increased when acquiring images of PDS-treated samples (but not in untreated controls) in order to compensate for the reduced telomeric FISH signal and to enable quantification of fragile telomeres. G4 stabilization significantly enhanced the telomere fragility characteristic of *Brca2*-deleted MEFs (Figure 2B), suggesting that persistent G4 structures contribute to the telomere replication defect intrinsic to cells lacking BRCA2.

We next monitored the viability of *Brca2*-deleted MEFs grown in the presence of PDS or poly (ADP-ribose) polymerase 1 (PARP1) inhibitor olaparib. Even though PDS was moderately toxic to BRCA2-proficient MEFs, we detected a more prominent dose-dependent reduction in the viability of Cre-treated *Brca2*^*F/-*^ MEFs exposed to this compound or olaparib (Figure 2C). The same specific elimination by PDS was observed for BRCA2-deficient V-C8 hamster cells (Kraakman-van der Zwet et al., 2002; Figure S2A) and *Brca2*^*−/−*^ mouse mammary tumor-derived cells (Figure S2B).

The tumor suppressor BRCA1 plays a key role in HR by promoting end resection, which enables loading of the RAD51 recombinase and initiation of HR-mediated repair. This activity of BRCA1 is antagonized by 53BP1, which protects broken DNA ends and channels their repair into non-homologous end joining (NHEJ) (Bouwman et al., 2010, Bunting et al., 2010). To address whether NHEJ deficiency also sensitizes cells to G4 stabilizing agents, similarly to HR ablation, we tested whether *Brca1* or 53BP1 loss confers sensitivity to PDS. Only viability of *Brca1*-deleted cells was affected by exposure to PDS (Figures 2D and 2E), suggesting that G4 stabilization is specifically toxic to HR-, but not to NHEJ-compromised cells. A similar HR-specific effect was observed in response to olaparib (Figures 2D and 2E).

### G4-Interacting Compounds Specifically Kill HR-Deficient Human Cells

**Figure 3:**
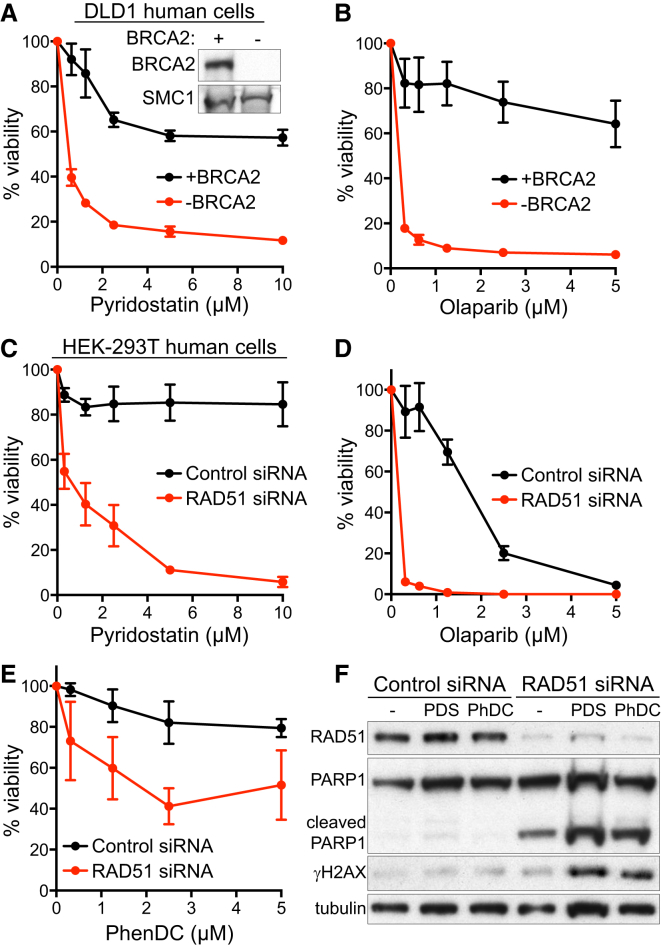
Effect of PDS on BRCA2- or RAD51-Deficient Human Cell Viability (A and B) Dose-dependent viability assays of DLD1 cells, BRCA2 proficient (+BRCA2) or deficient (−BRCA2), treated with indicated concentrations of PDS (A) or olaparib (B). (C–E) Dose-dependent viability assays of HEK293T cells transfected with control or RAD51 siRNA treated with indicated concentrations of PDS (C), olaparib (D), or PhenDC (E). Graphs shown are representative of at least two independent experiments, each performed in triplicate. Error bars represent SD of triplicate values obtained from a single experiment. (F) Whole-cell extracts prepared after 4 days of treatment with 2 μM PDS or PhenDC (PhDC) were immunoblotted as indicated. Tubulin was used as a loading control. See also Figure S2.

To investigate whether PDS-induced G4 stabilization affects viability of human cells lacking BRCA2, we used a matched pair of BRCA2-proficient and deficient DLD1 colorectal adenocarcinoma cell lines (Hucl et al., 2008). Exposure of BRCA2-deficient DLD1 cells to PDS led to a marked decrease in viability compared to BRCA2-proficient cells within 3 days (Figure S2C), which became more pronounced after six days of treatment (Figure 3A). The PARP1 inhibitor olaparib was used as a control in these experiments based on its ability to preferentially kill BRCA2-deficient cells (Figure 3B). Importantly, PDS toxicity to cells lacking BRCA2 was recapitulated in clonogenic assays in which cells were exposed to the drug for only 24 hr (Figure S2D).

BRCA2 plays a central role in HR repair by recruiting RAD51 to the sites of DSBs ssDNA present at stalled replication forks to initiate strand-invasion reactions. We therefore investigated whether RAD51 deficiency sensitized cells to G4-interacting compounds, similarly to loss of BRCA2. Indeed, exposure to PDS caused a substantial drop in cell viability of HEK293T cells lacking RAD51 compared to control cells (Figures 3C and S2C). Olaparib reduced the viability of RAD51-depleted cells; however, it also exhibited toxicity against control cells (Figure 3D). Moreover, RAD51 depletion sensitized HEK293T cells to the G4 ligand PhenDC (Figure 3E; Piazza et al., 2010). In western blot analyses (Figure 3F), PDS and PhenDC both induced apoptosis specifically in RAD51-deficient cells, detected by cleaved PARP1 and γH2AX expression, a well-established marker for DNA damage that is also induced by apoptosis (Rogakou et al., 2000). Thus, treatment with G4-interacting agents elicits DNA damage leading to specific killing of cells lacking BRCA2 or RAD51. While PhenDC drastically reduced viability of *Brca1*^*−/−*^ mouse tumor-derived cells (Figure S2E), its toxicity against BRCA2-deficient V-C8 cells was rather modest (Figure S2A).

### PDS Enhances DNA Damage Levels in HR-Compromised Cells

**Figure 4:**
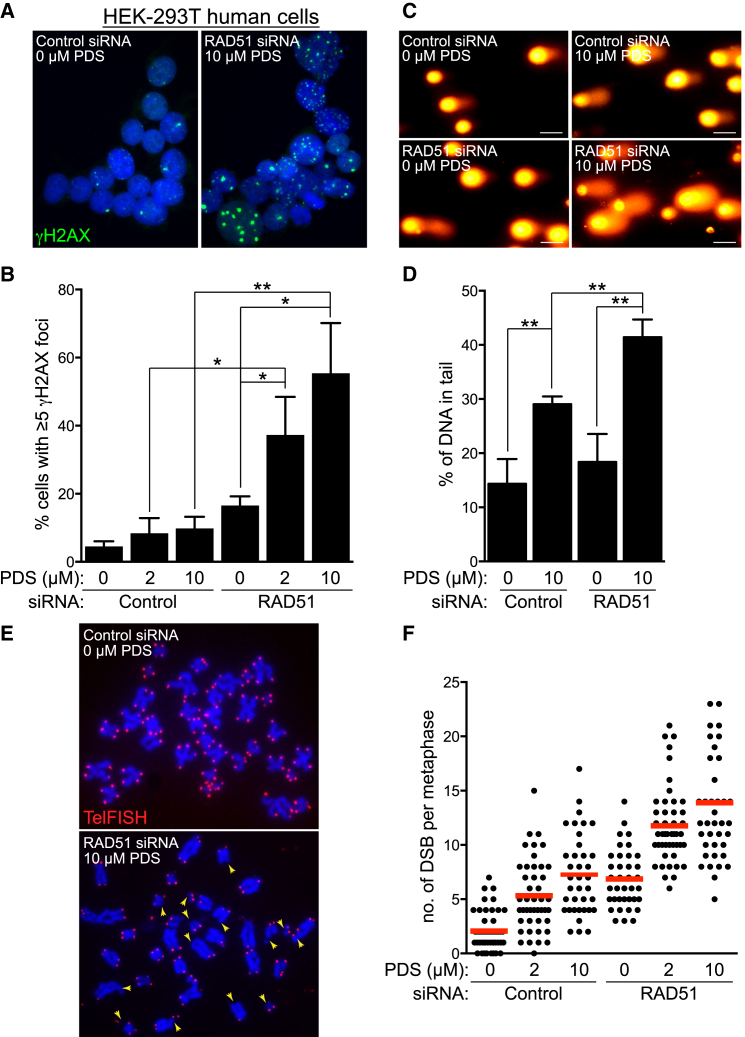
Elevated Levels of DNA Damage in RAD51-Deficient Human Cells Treated with PDS (A) Representative images of HEK293T cells transfected with control or RAD51 siRNA and treated with PDS for 4 days before processing for immunofluorescence staining with anti-γH2AX antibody (green). DNA was counterstained with DAPI (blue). (B) Quantification of the frequency of cells with ≥5 γH2AX foci treated as in (A); n = 3; error bars, SD. p values were calculated using an unpaired two-tailed t test (^∗^p ≤ 0.05; ^∗∗^p ≤ 0.01). (C) Representative images of cells treated as in (A) processed for comet assays. Scale bar, 50 μm. (D) Quantification of tail moment using comet assays of cells treated as in (A); n = 3; error bars, SD. p values were calculated using an unpaired two-tailed t test (^∗^p ≤ 0.05). (E) Representative images of FISH analysis of metaphase chromosome spreads of cells treated as in (A) with a Cy3-conjugated telomeric probe (red). DNA was counterstained with DAPI (blue). Arrowheads point to chromatid/chromosome breaks. (F) Quantification of mean DSB frequencies (red bars) in cells treated as in (A). Approximately 40 metaphases were analyzed for each sample. See also Figure S3.

We next focused on understanding the mechanism underlying the impaired viability of RAD51-deficient cells in the presence of PDS. Quantification of γH2AX foci as detected by immunofluorescence staining (Figures 4A and S3A) revealed a significant increase in the frequency of HR-deficient cells containing γH2AX foci in response to PDS (Figure 4B). On average, 16.5% of untreated RAD51-depleted cells exhibited five or more γH2AX foci, which escalated to 37.3% and 55.4% following treatment with 2 or 10 μM PDS, respectively. In control cells, the focal γH2AX accumulation upon PDS treatment was not statistically significant (from 4.5% to 8.2% and 9.7%). Alkaline comet assays, in which the percentage of tail DNA relative to total DNA was indicative of the levels of DNA damage present in an individual cell (Figure 4C), confirmed that PDS-triggered DNA damage was significantly augmented in HR-deficient compared to HR-proficient cells (Figure 4D). In agreement with this, PDS elicited increased numbers of DBSs per metaphase in control cells, and RAD51 depletion further enhanced this effect (Figures 4E, 4F, and S3B). In these images we used telomeric FISH probes that helped define individual chromosomes. Given the reduced intensity of the FISH signal for the telomeric G-rich strand in PDS-treated samples, we increased acquisition time for these images, as described for Figure 2B. The average number of breaks detected in this assay reflects break accumulation in mitosis, while cells with higher levels of DNA damage most likely arrest during G2/M transition. Consistently, PDS treatment and RAD51 depletion caused a decrease in the mitotic index (Figure S3C). Taken together, these data supported the concept that G4 stabilization triggers DNA damage, with lethal consequences in cells with compromised capacity for HR-mediated repair.

### Acute Replication Stress Induced by PDS in Cells Lacking RAD51 or BRCA2

**Figure 5:**
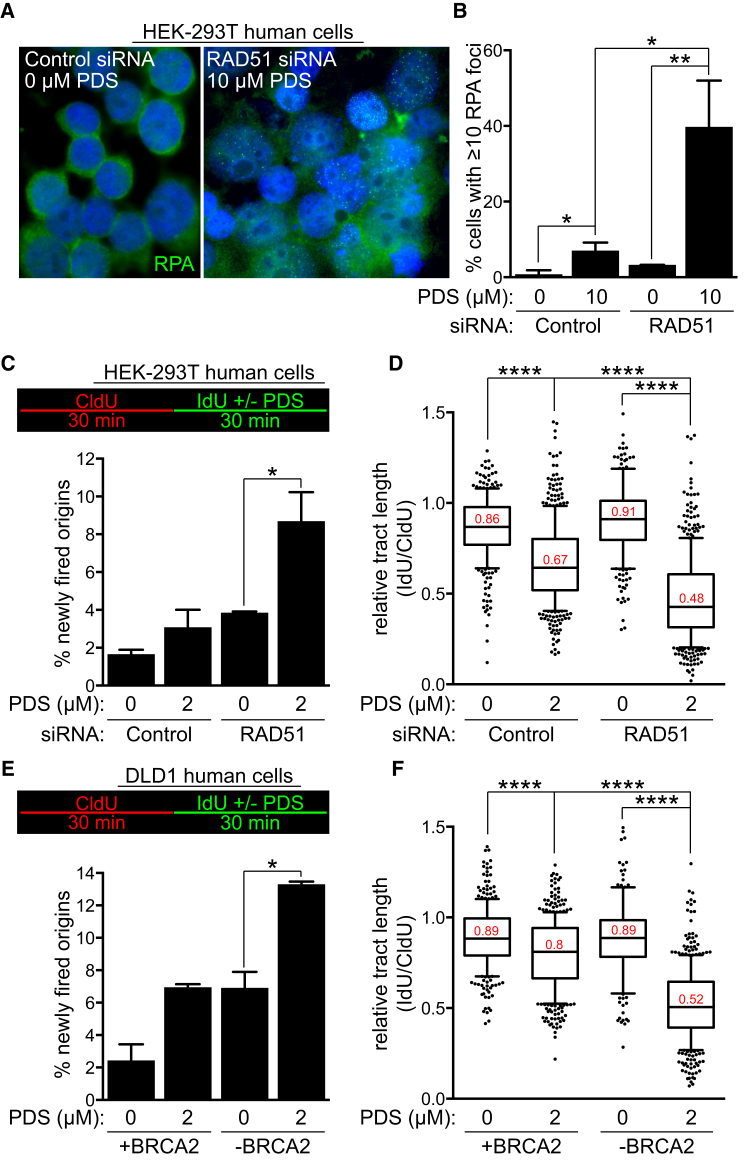
PDS Exacerbates the Replication Defect of RAD51- and BRCA2-Deficient Human Cells. (A) Representative images of HEK293T cells transfected with control or RAD51 siRNA and treated with PDS for 4 days before processing for immunofluorescence staining with anti-RPA antibody (green). DNA was counterstained with DAPI (blue). (B) Quantification of the frequency of cells with ≥10 RPA foci treated as in (A); n = 3; error bars, SD. p values were calculated using an unpaired two-tailed t test (^∗^p ≤ 0.05; ^∗∗^p ≤ 0.01). (C) HEK293T cells transfected with control or RAD51 esiRNA were processed for DNA fiber analysis as outlined in the inset, followed by quantification of the frequency of newly fired origins (n = 2; error bars, SD). p values were calculated using an unpaired two-tailed t test (^∗^p ≤ 0.05). (D) Quantification of the relative replication tract length (IdU/CldU) in cells treated as in (C). Middle line represents median, and the box extends from the 25^th^ to 75^th^ percentiles. The whiskers mark the 10^th^ and 90^th^ percentiles. p values were calculated using a Mann-Whitney test (n = 2; ^∗∗∗∗^p < 0.0001). (E) DLD1 cells, BRCA2 proficient (+BRCA2) or deficient (−BRCA2), were processed for DNA fiber analysis as outlined in the inset, followed by quantification of the frequency of newly fired origins (n = 2; error bars, SD). p values were calculated using an unpaired two-tailed t test (^∗^p ≤ 0.05). (F) Quantification of the relative replication tract length (IdU/CldU) in cells treated as in (E). Middle line represents median, and the box extends from the 25^th^ to 75^th^ percentiles. The whiskers mark the 10^th^ and 90^th^ percentiles. p values were calculated using a Mann-Whitney test (n = 2; ^∗∗∗∗^p < 0.0001). See also Figure S4.

PDS has been proposed to induce replication-dependent DNA damage (Rodriguez et al., 2012). This prompted us to monitor the assembly of replication protein A (RPA) subnuclear foci (Figures 5A and S4A) as a readout for genome-wide ssDNA accumulation. PDS induced an approximately 6-fold increase in the levels of RPA foci in control cells and approximately 12-fold increase in RAD51-deficient cells (Figure 5B). RPA accumulation on the chromatin, together with elevated frequency of origin firing and reduced replication rates, represents signatures of replicative stress (Zeman and Cimprich, 2014). To define the nature of this replication defect, we performed DNA fiber analyses in which replication tracks were labeled with consecutive 30 min pulses of CldU and IdU. Addition of PDS during the second pulse enabled us to evaluate the immediate effect of G4 stabilization on replication. Relative tract length was decreased significantly in PDS-treated cells compared to untreated cells, an effect that was more prominent in cells lacking RAD51 or BRCA2 expression (Figures 5D, 5F, S4B, and S4C). PDS may induce persistent G4s that reduce replication rate or cause DNA breakage that obstructs replication fork progression. Possibly as a compensatory mechanism, PDS treatment significantly increased the number of newly fired origins, detected as green tract only, specifically in RAD51- (Figure 5C) or BRCA2-deficient cells (Figure 5E). Notably, elevated origin firing was also detected in untreated HR-deficient cells. Thus, the replication stress endogenous to HR-compromised cells may be potentiated by chemical G4 stabilization to levels that become lethal. To test this possibility, we used aphidicolin as an alternative means to elicit replication stress (Figure S4D). Treatment with a nontoxic dose of aphidicolin led to sensitization of BRCA2-proficient cells to PDS. The synergy between the two compounds was not observed in BRCA2-deficient cells. This suggested that BRCA2 abrogation and aphidicolin treatment cause equivalent levels of replication stress and DNA damage, leading to comparable outcomes in the context of G4 stabilization by PDS.

### PDS Triggers Checkpoint Activation and G2/M Arrest in HR-Defective Cells

**Figure 6:**
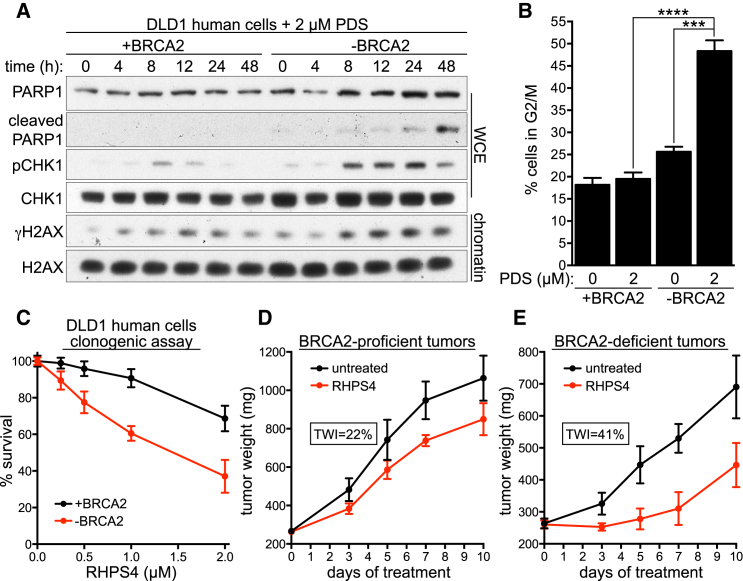
Effect of PDS on Viability of BRCA2-Deficient Cells and Tumors (A) DLD1 cells, BRCA2 proficient (+BRCA2) or deficient (−BRCA2), were incubated with 2 μM PDS. Whole-cell extracts (WCE) or chromatin fractions prepared at indicated time points were immunoblotted as shown. (B) Cells treated as in (A) were processed for FACS analyses of DNA content after 48 hr. Quantification of the percentage of cells in G2/M is shown (n = 3; error bars, SD). p values were calculated using an unpaired two-tailed t test (^∗∗∗^p ≤ 0.001; ^∗∗∗∗^p ≤ 0.0001). (C) Clonogenic survival assays of DLD1 cells, BRCA2 proficient (+BRCA2) or deficient (−BRCA2), exposed to the indicated concentrations of RHPS4 for 24 hr. Error bars represent SD of triplicate values obtained from a single experiment. (D and E) Mean tumor weights in untreated and RHPS4-treated mice injected with BRCA2-proficient (+BRCA2; D) or deficient (−BRCA2; E) DLD1 cells (n = 8; error bars, SD). Tumor weight inhibition (TWI) was calculated at the time point of maximum effect. See also Figures S5 and S6.

Given the profound antiproliferative effect of PDS in BRCA2- or RAD51-deficient cells, we examined its impact on the DNA damage response (DDR). In cells lacking BRCA2 or RAD51 expression, continuous PDS treatment for 4 days elicited a robust phosphorylation of KAP1 (Ser824), CHK1 (Ser314/345), and RPA (Ser4/8), indicative of ATM/ATR checkpoint activation, as well as PARP1 cleavage, a marker for apoptosis (Figures S5A and S5B). To establish whether DDR preceded apoptosis onset, we monitored the response to PDS over a 48 hr interval. In BRCA2-deficient cells, PDS triggered H2AX and CHK1 phosphorylation after 8 hr of treatment, whereas PARP1 cleavage was initiated between 24 hr and 48 hr (Figure 6A). RAD51-depleted HEK293T cells similarly exhibited γH2AX activation prior to PARP1 cleavage (Figure S5C). These results indicate that PDS-induced DDRs are provoked prior to apoptosis in cells lacking BRCA2 or RAD51. Accordingly, BRCA2- and RAD51-deficient cells accumulated in G2/M after PDS treatment (Figures 6B and S6A). A decrease in S-phase cells further reflected the effect of PDS on cell-cycle progression and checkpoint activation specifically in HR-deficient cells (Figures S6A and S6B). PDS induces replication-associated DSBs, although transcription-related DNA damage may accumulate in stages of the cell cycle other than S phase (Rodriguez et al., 2012). To address whether PDS causes damage in noncycling cells, G0/G1 arrest was induced by serum starvation in the presence or absence of PDS. Arrested cells lacked the ability to incorporate the thymidine analog EdU, in contrast to cells released into the cell cycle by serum addition to the media (Figure S6C). Quantification of γH2AX-positive cells demonstrated that PDS treatment for 48 hr did not induce DNA damage in noncycling cells, while a marked increase in the percentage of cells expressing γH2AX was detected in the subset of cycling cells treated with PDS (Figure S6C).

### In Vivo Responses of BRCA2-Deficient Tumors to G4 Ligands

Regardless of the effective suppression of HR-deficient cell viability and survival by PDS-mediated G4 stabilization (Figures 3A and S2D), the efficacy of PDS could not be established in vivo due to its toxicity predicted by in vitro studies (Rodriguez et al., 2012). Instead, we tested in our cellular models a previously reported G4-stabilizing drug, RHPS4 (Gavathiotis et al., 2003, Gowan et al., 2001, Heald et al., 2002), with well-characterized pharmacological features (Leonetti et al., 2008, Salvati et al., 2007). RHPS4 markedly diminished survival of BRCA2-deficient DLD1 cells relative to BRCA2-proficient cells (Figure 6C). To test its efficacy in vivo, DLD1 cells were injected into mice and allowed to form palpable tumors. In line with previous publications reporting the antitumor effect of RHPS4 (Leonetti et al., 2008, Salvati et al., 2007), this drug repressed growth of BRCA2-proficient tumors as assessed by tumor weight inhibition (TWI) (22%, Figure 6D). Importantly, the growth inhibitory effect of RHPS4 was almost twice as pronounced in BRCA2-deficient tumors (TWI = 41%, Figure 6E). RHPS4 treatment elicited a marked delay in tumor regrowth (approximately 7 days in BRCA2-deficient compared to 4 days in BRCA2-proficient tumors). Thus, our conclusions based on cellular models can be translated in vivo and support the concept that G4-stabilizing compounds identify a class of drugs, which may facilitate future development of novel therapeutic strategies for targeting BRCA2-deficient tumors.

### PDS Kills Olaparib-Resistant Tumor-Derived Cells

**Figure 7:**
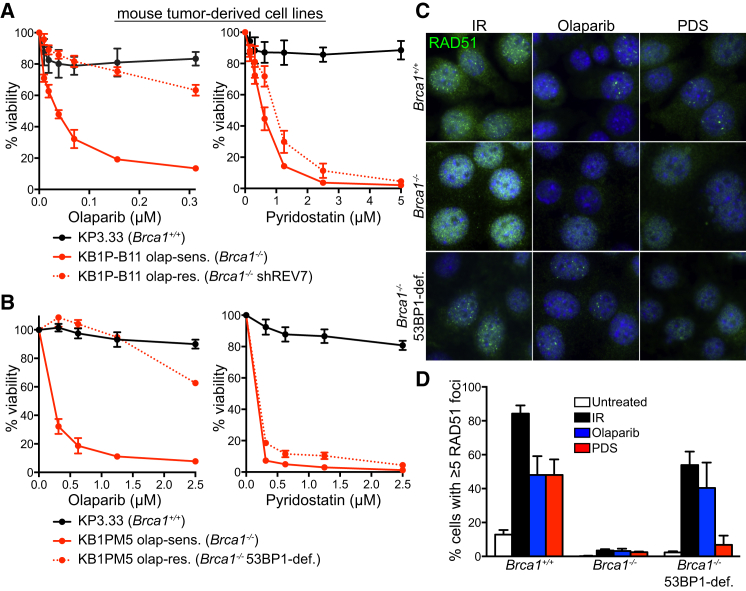
Olaparib-Resistant *Brca1*-Deleted Tumor Cells Exhibit PDS Sensitivity (A and B) Dose-dependent viability assays of mouse mammary tumor-derived cell lines deficient in REV7 (A) or 53BP1 (B) treated with indicated concentrations of PDS or olaparib. Graphs shown are representative of at least two independent experiments, each performed in triplicate. Error bars represent SD of triplicate values obtained from a single experiment. (C) Representative images of cells described in (A) incubated with 0.5 μM olaparib (OLAP), PDS for 40 hr, or irradiated with 10 Gy of IR followed by 1 hr recovery and processed for immunofluorescence staining with anti-RAD51 antibody (green). DNA was counterstained with DAPI (blue). (D) Quantification of the frequency of cells with ≥5 RAD51 foci in cells treated as in (C); n = 2; error bars, SD; >200 nuclei were analyzed for each condition per replica. See also Figure S7.

Treatment of BRCA-deficient tumors poses a major challenge in the clinic due to the rapid emergence of drug resistance. To test the potential of PDS to eliminate *Brca1*-deficient mouse tumor-derived cells refractory to olaparib, we used two *Brca1*^*−/−*^ cellular mouse models, in which olaparib resistance was mediated by concomitant loss of REV7 (Figure 7A; Xu et al., 2015) or 53BP1 (Figure 7B; Jaspers et al., 2013). Cells carrying intact *Brca1* (*Brca1*^+/+^) showed no sensitivity to PDS or olaparib, while cells established from a *Brca1*^−/−^ tumor were sensitive to both drugs, as determined in viability and clonogenic assays (Figures 7A, 7B, S7A, and S7B). Strikingly, olaparib-resistant *Brca1*-deficient cells lacking REV7 or 53BP1 expression (*Brca1*^−/−^ shREV7; *Brca1*^−/−^ 53BP1-deficient) were hypersensitive to PDS (Figures 7A, 7B, S7A, and S7B). These effects were recapitulated in human cells, in which 53BP1 and BRCA1 were depleted using siRNA (Figure S7C). Our results, therefore, strongly suggest that BRCA1-deficient cells, including those resistant to PARP inhibitors, can be targeted by treatment with G4-stabilizing compounds.

HR restoration in *Brca1*-deleted cells and tumors is driven by 53BP1 loss, which enables survival (Bouwman et al., 2010, Bunting et al., 2010). Moreover, ionizing radiation (IR)-induced RAD51 foci assemble in olaparib-resistant *Brca1*^−/−^, 53BP1-deficient cells (albeit not at the same level as in *Brca1*^+/+^ cells), but not in olaparib-sensitive *Brca1*^−/−^ tumor-derived cells (Jaspers et al., 2013). Our data (Figures 7C and 7D) demonstrate that olaparib treatment itself triggers RAD51 foci in wild-type and olaparib-resistant, but not olaparib-sensitive, cells, thereby providing a direct correlation between olaparib-induced HR reactivation and its impact on cell survival. PDS treatment induced RAD51 foci in *Brca1*^+/+^ cells, similarly to olaparib (Figures 7C and 7D). However, RAD51 foci were absent in both olaparib-sensitive and olaparib-resistant cells upon treatment with PDS (Figures 7C and 7D), suggesting that failure to reactivate HR repair contributes to the toxicity of this compound in *Brca1*^−/−^, 53BP1-deficient cells. To gain further insight into the mechanism of RAD51 foci suppression, we evaluated the levels of chromatin-associated RPA, indicative of end resection activity. In the chromatin fraction of PDS-treated cells, less RPA was detected than in cells exposed to olaparib or IR (Figure S7D). Thus, impaired HR reactivation upon PDS treatment in a *Brca1*^−/−^, 53BP1-deficient background is likely caused by defects in end resection.

## Discussion

The ability of G-rich DNA to adopt G4 secondary structures in vitro was reported over 50 years ago (Gellert et al., 1962). Although G4s are thought to positively regulate key cellular processes, they can also obstruct replication-fork progression, leading to genomic instability (Tarsounas and Tijsterman, 2013). In this study, we establish that effective replication of G4 structures requires HR activities. G4s represent potent replication barriers, and HR provides a well-characterized mechanism for replication-fork restart and repair of replication-associated DSBs. Yet, the potential requirement for HR in G4 stability has not been investigated, with the notable exception of *Saccharomyces cerevisiae pif1* mutants, in which attempts to restart forks stalled in the vicinity of G4 structures generated recombination intermediates. This suggested a role for HR in fork restart when Pif1 activity is abrogated (Ribeyre et al., 2009).

### HR Is Required for Effective Replication of Genomic Regions with G4-Forming Potential

HR factors have previously been implicated in telomere maintenance (Tacconi and Tarsounas, 2015). In the present work, we used a plasmid-based replication assay in human cells to show that replication of telomeric repeats is ineffective when key HR activities are abrogated. Two lines of evidence established the HR requirement for replication of the G-rich telomeric strand. First, telomere fragility triggered by HR gene deletion was specific to the G-rich telomeric strand, which possesses G4-forming potential. Second, disruption of the G4-forming telomeric repeats through G-to-C substitutions rescued its replication defect in HR-deficient cells.

We propose that HR promotes replication in the presence of obstructive G4 structures by restarting stalled forks and/or by repairing replication-associated DSBs within telomeres, rather than contributing to telomeric G4 dissolution per se. The latter process is likely mediated by the shelterin component TRF1, which recruits BLM helicase to telomeres to unwind G4 structures (Zimmermann et al., 2014). The concept that HR and shelterin provide distinct mechanisms for telomere replication is supported by the synthetic lethality observed between *Brca2* and *Trf1* gene deletions in immortalized MEFs, accompanied by additive levels of telomere fragility (Badie et al., 2010). Inhibition of BLM expression with shRNA in *Brca2*-deleted cells similarly induced cell-cycle arrest (J.Z. and M.T., unpublished data), further arguing that independent mechanisms act during telomere replication to dismantle G4s and to repair the DNA damage induced by persistent G4 structures.

Importantly, G4 stabilization by PDS reduced viability of mouse, human, and hamster cells lacking BRCA1, BRCA2, or RAD51. It exacerbated telomere fragility and DNA damage levels in HR-deficient cells. Conceivably, unresolved G4s presenting intrachromosomally or within telomeres are converted to DSBs, eliciting in turn checkpoint activation, cell-cycle arrest, and/or specific elimination of HR-compromised cells by apoptotic mechanisms.

The efficacy of PDS in cell killing was previously attributed to its genome-wide toxicity, suggested by the accumulation of DNA damage marker γH2AX at genomic sites with computationally inferred G4-forming sequences (Rodriguez et al., 2012). It is conceivable that the same sites may be prone to breakage in HR-deficient cells treated with PDS. Our mitotic DSB quantification illustrates the additive effect of PDS on the levels of DNA damage triggered by HR abrogation itself. A conundrum posed by this quantification was that PDS induced approximately fifteen DSBs per metaphase in cells lacking RAD51, yet in silico predictions suggested that more than 300,000 genomic sites can adopt G4 configurations (Huppert and Balasubramanian, 2005). This discrepancy could be explained by the multitude of mechanisms known to maintain genome integrity by dismantling G4s formed during genome replication (Tarsounas and Tijsterman, 2013). While most genomic G4s are dissolved by alternative mechanisms, our data suggest that a subset triggers fork stalling and DSBs, which are particularly toxic in HR-deficient cells lacking a key pathway of fork restart and break repair. G4-induced DNA damage may be repaired by error-prone mechanisms in the absence of HR, which seems insufficient for the survival of these cells. Moreover, checkpoint activation prevented entry of cells with elevated DSB levels into mitosis, which further justifies the lower number of mitotic DSBs detected in our assay.

### Implications for Cancer Therapies

The work presented here demonstrates that the G4-stabilizing drug RHPS4 limits the growth of BRCA2-deficient tumors grafted in mice. The well-characterized ability of RHPS4 to trigger telomere dysfunction may contribute to its toxicity to BRCA2-deficient cells (Salvati et al., 2007). Therefore, we propose that the anticancer potential of the G4-stabilizing drug RHPS4 can be exploited in the clinic for specific targeting of BRCA2-deficient tumors. This tumor subset is likely to benefit most from this novel class of anticancer drugs. Furthermore, these results open a favorable prospective for future clinical development of PDS into a drug-like compound, with a more robust anticipated antitumor activity than RHPS4 in models for BRCA2 inactivation.

Mutations in HR genes such as *BRCA1*, *BRCA2*, or *RAD51C* predispose individuals to breast and ovarian cancers. Tumors carrying HR gene deletions are vulnerable to drugs that either introduce replication-associated DNA damage (e.g., platinum drugs) or inhibit DNA repair pathways other than HR (e.g., PARP1 inhibitors, such as olaparib). In both cases, excessive DNA-damage accumulation triggers cell death. Here, we propose that G4-binding compounds identify a novel class of molecules that can be used to target BRCA deficiency. They act by stabilizing secondary structures in genomic regions with high G-rich content, thus reducing replication fork speed and inducing RPA foci indicative of ssDNA accumulation. *BRCA* gene abrogation is associated with the same responses (Carlos et al., 2013). In the absence of HR, G4-interacting compounds are likely to elevate the endogenous replication stress to levels that become lethal due to excessive DNA-damage accumulation.

One well-documented caveat of targeted drug treatments, such as olaparib, is that tumors rapidly acquire resistance through mechanisms that include activation of P-glycoprotein drug efflux transporter, genetic *Brca1/2* re-activation, and loss of 53BP1/REV7 (Bouwman and Jonkers, 2014, Jaspers et al., 2013, Xu et al., 2015). In this work, we establish that G4-stabilizing compounds are profoundly toxic to BRCA-defective cells, including those resistant to PARP inhibitors. In particular, the striking cytotoxicity of PDS is due to the combined replication failure induced by this drug and the DNA repair defect associated with HR abrogation. Therefore, pharmacological G4 stabilization could be exploited in future therapeutic modalities targeting this difficult to treat tumor subset. Olaparib-resistant cells fail to reactivate HR in response to PDS, which may account for the lethality induced by this G4-stabilizing compound. We therefore anticipate that further clinical development of G4-stabilizing compounds will enhance their ability to selectively eliminate HR-compromised tumors, including those that have acquired resistance to existing therapies.

## Experimental Procedures

For detailed descriptions of these and additional procedures, see Supplemental Experimental Procedures.

### Cell Lines, Culture Conditions, and In Vivo Experiments

HEK293T, H1299, and DLD1 cells were cultured under conventional growth conditions. In vivo experiments were performed as previously described (Salvati et al., 2007). All animal procedures were in compliance with the national and international directives (D.L. March 4, 2014, no. 26; directive 2010/63/EU of the European Parliament and of the council; Guide for the Care and Use of Laboratory Animals, United States National Research Council, 2011).

### Plasmid-Based Replication Assay

Plasmid-based replication assays were performed as previously described (Sarkies et al., 2010, Szüts et al., 2008) with modifications listed in Supplemental Experimental Procedures.

### RNAi

DLD1 and HEK293T cells were transfected with 40 nM siRNA using Dharmafect 1 (Dharmacon) according to manufacturer’s instructions.

### Cell Viability Assays

Cell viability was determined by incubation with 10 μg/ml of resazurin for 2 hr. Fluorescence was measured at 590 nm using a plate reader (POLARstar, Omega one). Cell viability was expressed relative to untreated cells of the same cell line, thus accounting for any differences in viability caused by HR deficiency. Graphs shown are representative of at least two independent experiments, each performed in triplicate. Error bars represent SD of triplicate values obtained from a single experiment.

### FACS Analysis

Cells were harvested by trypsinization, washed in cold PBS, and fixed in ice-cold 70% ethanol overnight at 4°C. Following two washes in PBS, cells were incubated with 20 μg/ml propidium iodide and 10 μg/ml RNase A (Sigma) in PBS. At least 10,000 cells were analyzed by flow cytometry (Becton Dickinson). Data were processed using CellQuest (Becton Dickinson) and ModFit LT software.

### Alkaline Single-Cell Gel Electrophoresis Comet Assay

The comet assay was performed as previously described (Singh et al., 1988). Tail measurement was performed using the Komet 5.5 image analysis software.

### Immunofluorescence

Cells were subjected to immunofluorescence staining as described (Tarsounas et al., 2004).

### Preparation of Metaphase Spreads and Telomere FISH

Metaphase spread preparation and telomeric FISH were performed as previously described (Badie et al., 2015).

### Chromosome Orientation FISH and IF-FISH

For CO-FISH, cells were plated at 50%–60% confluency and treated with 10 μM bromodeoxyuridine (BrdU) for 20 hr. Colcemid (0.2 μg/ml) was added to the cells 4–6 hr before metaphases were processed for CO-FISH as previously described (Bailey et al., 2001).

For IF-FISH, metaphases were spun onto coverslips using a cytospin apparatus (Cytospin 4, Fisher) and subjected to immunofluorescence staining as described (Tarsounas et al., 2004). Samples were fixed again in 4% paraformaldehyde in PBS, and FISH was performed as described (Tarsounas et al., 2004) using 15 μg/ml Cy3-conjugated (CCCTAA)_6_-PNA telomeric probe (Applied Biosystems).

### DNA Fiber Assay

DNA fiber assays were performed as described previously (Jackson and Pombo, 1998).

### Immunoblotting

SDS-PAGE and immunoblotting were performed as previously described (Badie et al., 2015). See Supplemental Experimental Procedures for antibodies used in this study.

## Supplemental Information

Document S1. Supplemental Experimental Procedures and Figures S1–S7

Document S2. Article plus Supplemental Information
