# Supplementary material for: Targeting BRCA1 and BRCA2 Deficiencies with G-Quadruplex-Interacting Compounds
Source: Mol Cell. 2016 Feb 4;61(3):449–60. doi: 10.1016/j.molcel.2015.12.004 (PMC4747901; doi:10.1016/j.molcel.2015.12.004)
Supplement: Document S1. Supplemental Experimental Procedures and Figures S1–S7 [file mmc1.pdf]

Molecular Cell

Supplemental Information

Targeting BRCA1 and BRCA2 Deficiencies with G-Quadruplex-Interacting Compounds

Jutta Zimmer, Eliana M. C. Tacconi, Cecilia Folio, Sophie Badie, Manuela Porru, Kerstin Klare, Manuela Tumiati, Enni Markkanen, Swagata Halder, Anderson Ryan, Stephen P. Jackson, Kristijan Ramadan, Sergey G. Kuznetsov, Annamaria Biroccio, Julian E. Sale, and Madalena Tarsounas

Figure S1 (related to Figures 1 and 2)

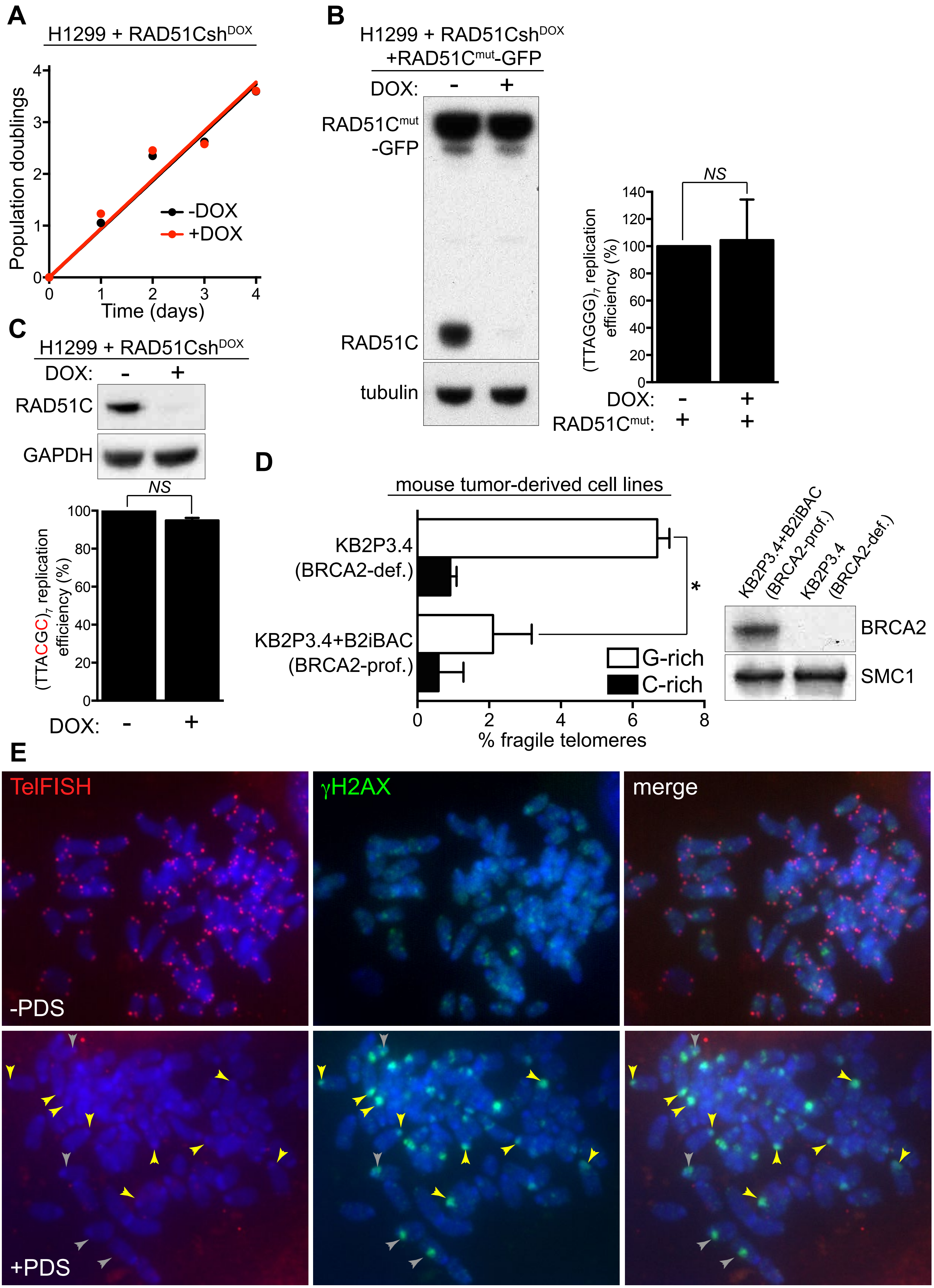

Figure S1 (related to Figures 1 and 2). (A) Proliferation curves of human H1299 cells expressing a doxycycline (DOX)-inducible RAD51C shRNA grown in the presence or absence of DOX. (B) Target specificity of the RAD51C shRNA and replication efficiency of mutated telomere sequences. H1299 cells expressing a DOX-inducible RAD51C shRNA were transfected with an shRNA-resistant RAD51C-GFP expression vector. Cell extracts prepared from cells grown in the presence or absence of DOX were immunoblotted as indicated. Tubulin was used as a loading control. Replication efficiency of a plasmid containing $(TTAGGG)_7$ was determined relative to the empty vector (*n*=2; error bars, SD). *P* values were calculated using an unpaired two-tailed *t*-test. NS, $P > 0.05$. (C) Cell extracts prepared from human H1299 cells expressing a DOX-inducible RAD51C shRNA grown in the presence or absence of DOX were immunoblotted as indicated. GAPDH was used as a loading control. Replication efficiency of a plasmid containing the $(TTACGC)_7$ array, in which G to C substitutions abolished the G4-forming potential of the telomeric repeats, was determined relative to the empty vector (*n*=2; error bars, SD). *P* values were calculated using an one-sample *t*-test. NS, $P > 0.05$. (D) Quantification of fragile telomeres on metaphase chromosome spreads prepared from a *Brca2*$^{-/-}$ mouse mammary tumor-derived cell line and BRCA2-reconstituted control cell line. Approximately 1000 telomeres were scored per sample (*n*=2; error bars, SD). *P* values were calculated using an unpaired two-tailed *t*-test. *, $P < 0.05$. Cell extracts were immunoblotted as indicated. SMC1 was used as a loading control. KB2P3.4, *Brca2*$^{-/-}$ mouse tumor-derived cell line; KB2P3.4+B2iBAC, *Brca2*$^{-/-}$ mouse tumor-derived cell line complemented with full-length BRCA2. (E) *p53*$^{-/-}$ MEFs grown for 48 h in the presence or absence of 5 μM PDS were arrested in mitosis with colcemid and mitotic chromosomes were spread using the cytospin method. Preparations were fixed and stained with an anti-γH2AX monoclonal antibody (green). Telomeres were visualized with a Cy3-conjugated $(CCCTAA)_6$-PNA probe (red), using identical exposure conditions for the two chromosome preparations. DNA was counter-stained with DAPI (blue).

Figure S2 (related to Figures 2 and 3)

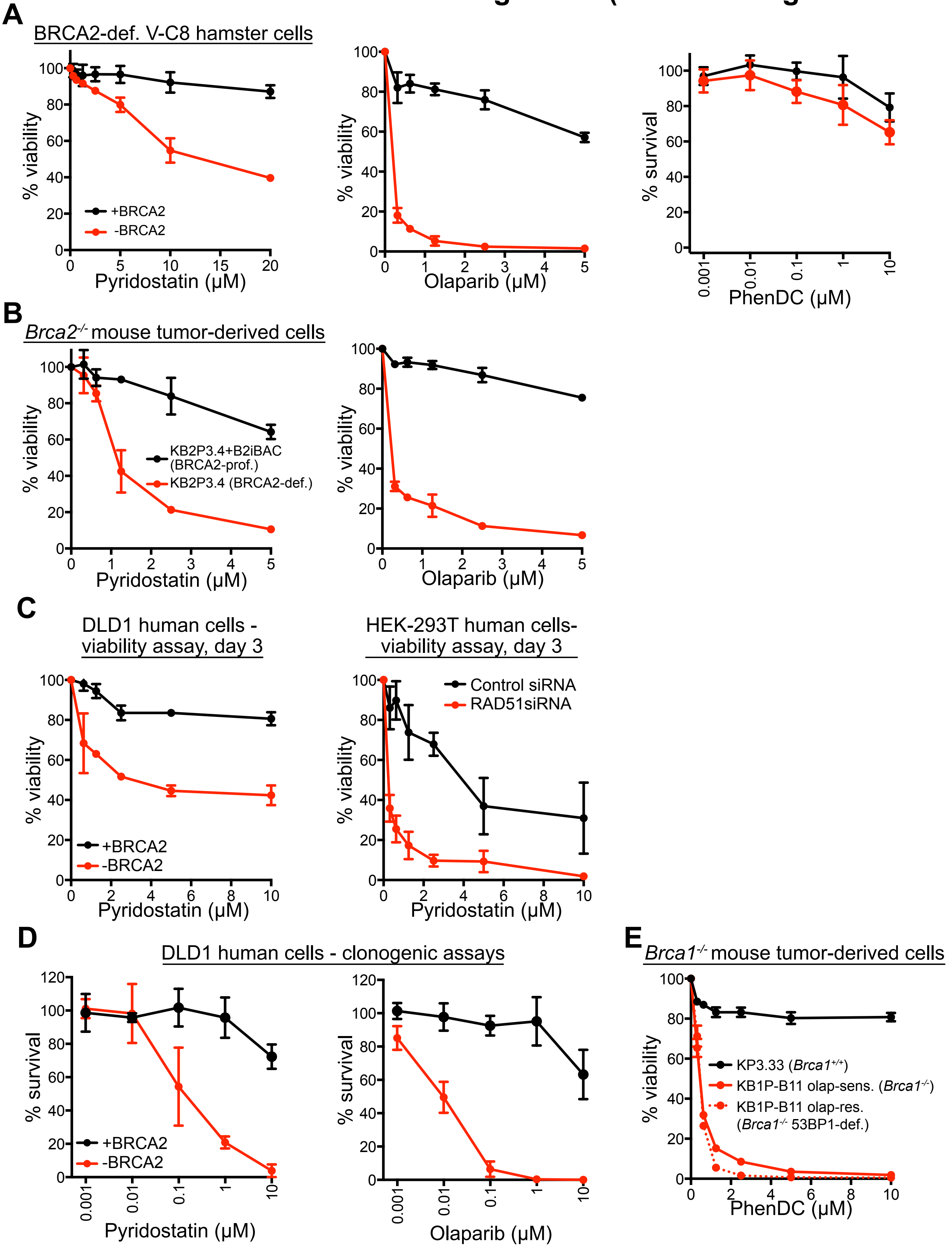

Figure S2 (related to Figures 2 and 3). (A) Dose-dependent viability and survival assays of BRCA2-deficient (-BRCA2) and -reconstituted (+BRCA2) hamster V-C8 cell lines. (B) Dose-dependent viability assays of *Brca2*$^{-/-}$ mouse mammary tumor-derived cell lines. (C) Dose-dependent viability assays of human cells treated with PDS for three days. Viability assays performed in the same cells after six days of treatment are shown in Figures 3A and 3C. (D) Clonogenic survival assays of human DLD1 cells treated with the indicated concentrations of PDS or olaparib for 24 h. Following removal of the drugs, cells were incubated in fresh media for 10-14 days before colony staining. (E) Dose-dependent viability assays of *Brca1*$^{-/-}$ mouse mammary tumor-derived cell lines. Graphs shown are representative of at least two independent experiments, each performed in triplicate. Error bars represent SD of triplicate values obtained from a single experiment.

Figure S3 (related to Figure 4)

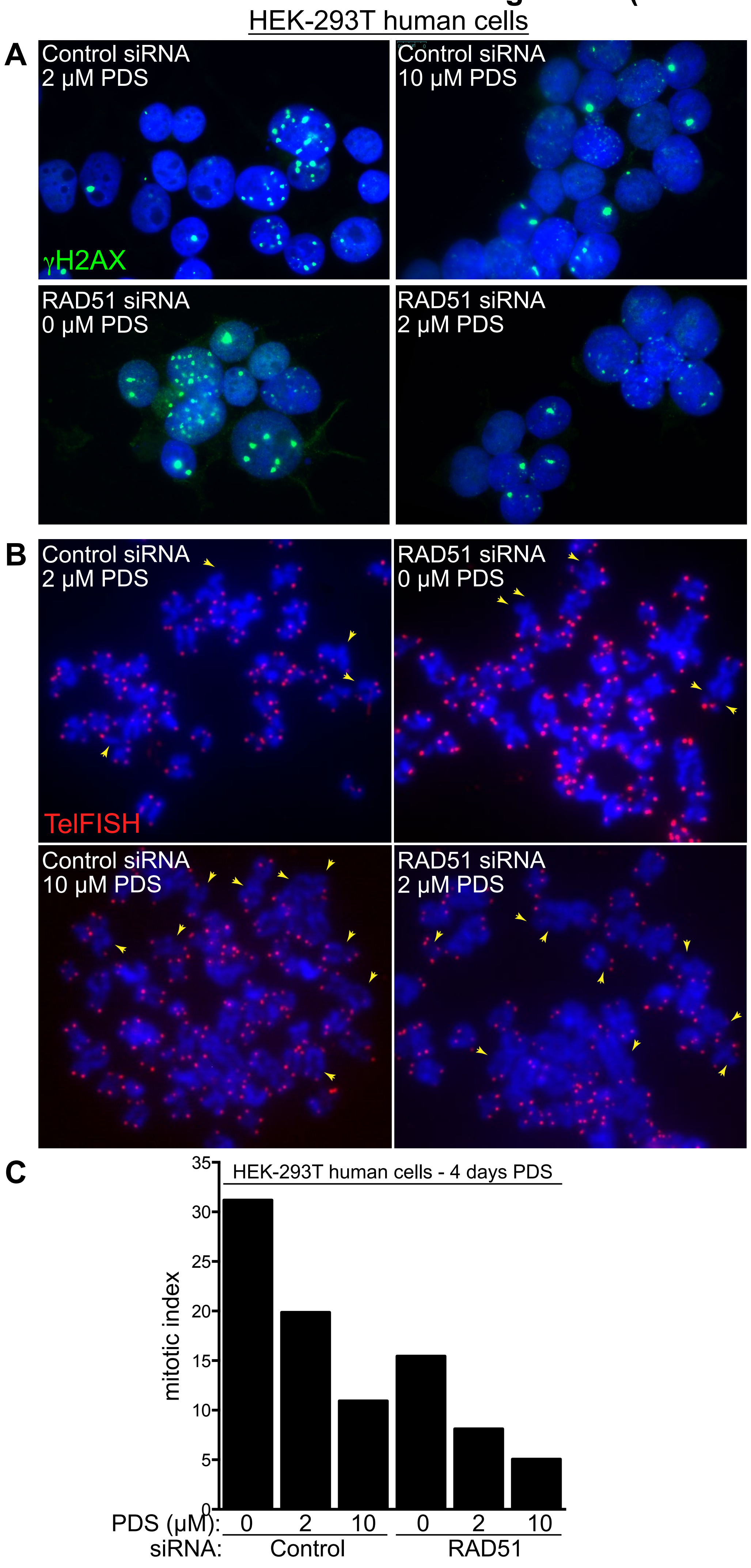

Figure S3 (related to Figure 4). (A and B) Additional representative images for the quantifications shown in Figures 4B (A) and 4D (B). (C) Human HEK-293T cells transfected with control or RAD51 siRNAs were treated with PDS for four days. Metaphase chromosome spreads were prepared by mitotic shake-off after colcemid treatment. DNA was stained with DAPI for quantification of mitotic nuclei. Mitotic index is expressed as % of total number of cells.

Figure S4 (related to Figure 5)

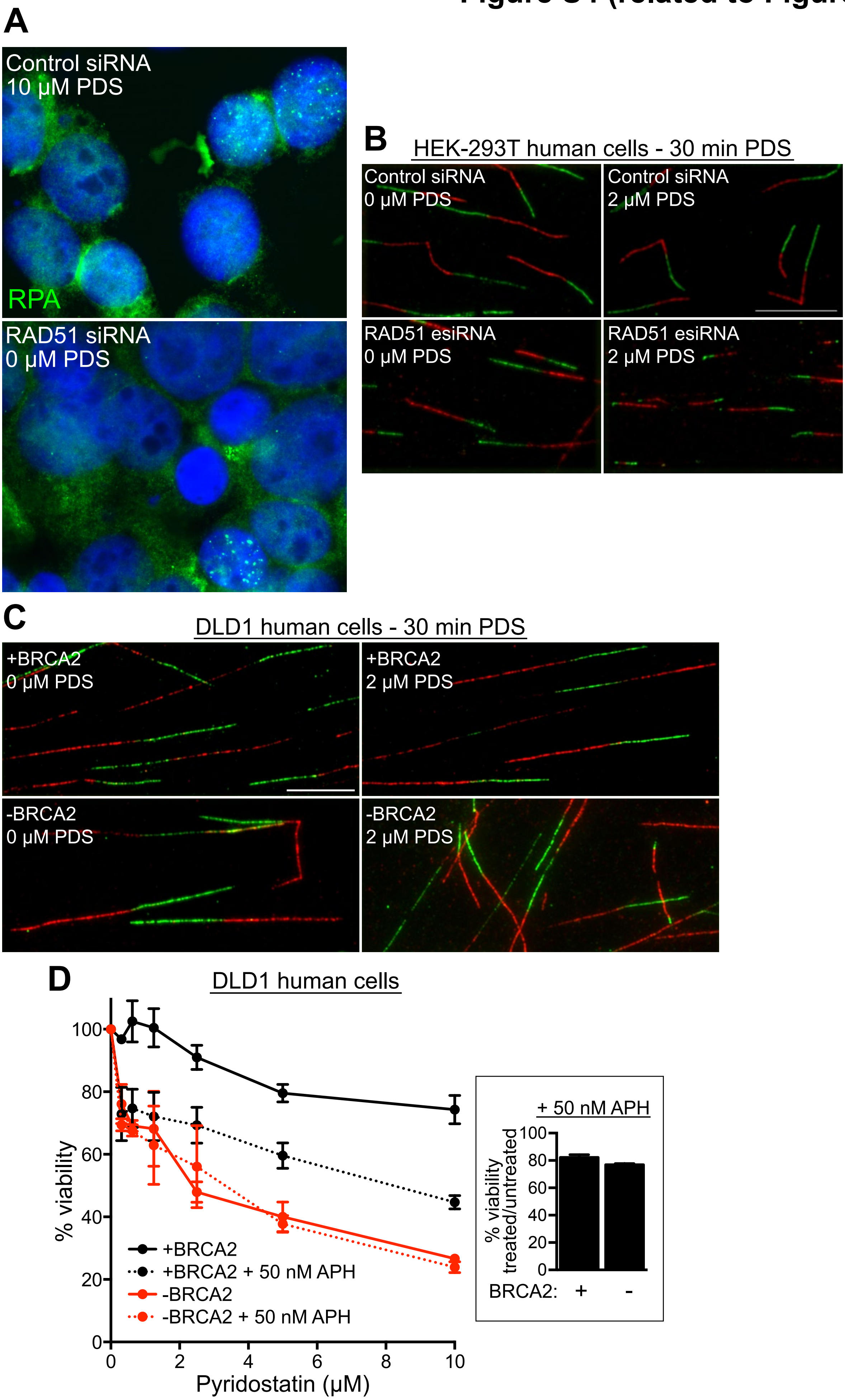

Figure S4 (related to Figure 5). (A) Additional representative images for the quantifications shown in Figure 5B. (B and C) Representative images for DNA fibers assays shown in Figures 5D (B) and 5F (C). Scale bar, 10 µm. (D) Dose-dependent viability assays of human BRCA2-proficient (+BRCA2) or -deficient (-BRCA2) DLD1 cells treated with PDS at the indicated concentrations in the presence or absence of 50 nM aphidicolin (APH). The effect of 50 nM APH alone is shown in the inset. Graphs shown are representative of three independent experiments, each performed in triplicate. Error bars represent SD of triplicate values obtained from a single experiment.

Figure S5 (related to Figure 6)

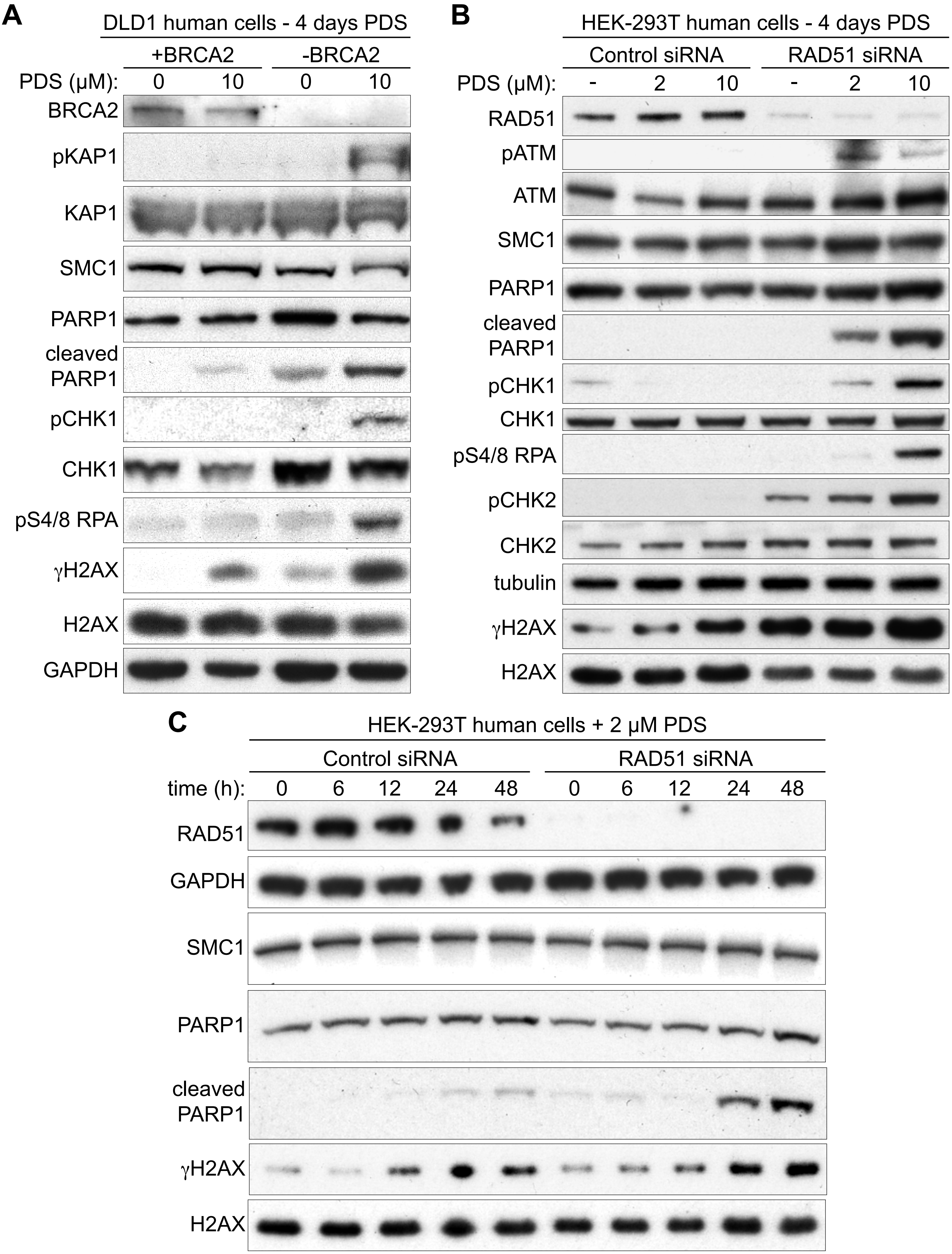

Figure S5 (related to Figure 6). (A) Cell extracts prepared from human DLD1 cells grown in the presence or absence PDS for four days were immunoblotted as indicated. SMC1 and GAPDH were used as loading controls. (B) Cell extracts of human HEK-293T human cells transfected with control or RAD51 siRNA were prepared after incubation with PDS for four days and immunoblotted as indicated. SMC1 and tubulin were used as loading controls. (C) Cell extracts of human HEK-293T cells transfected with control or RAD51 esiRNA were prepared at indicated time points after PDS addition and immu-unoblotted as shown. SMC1 and H2AX were used as loading controls.

Figure S6 (related to Figure 6)

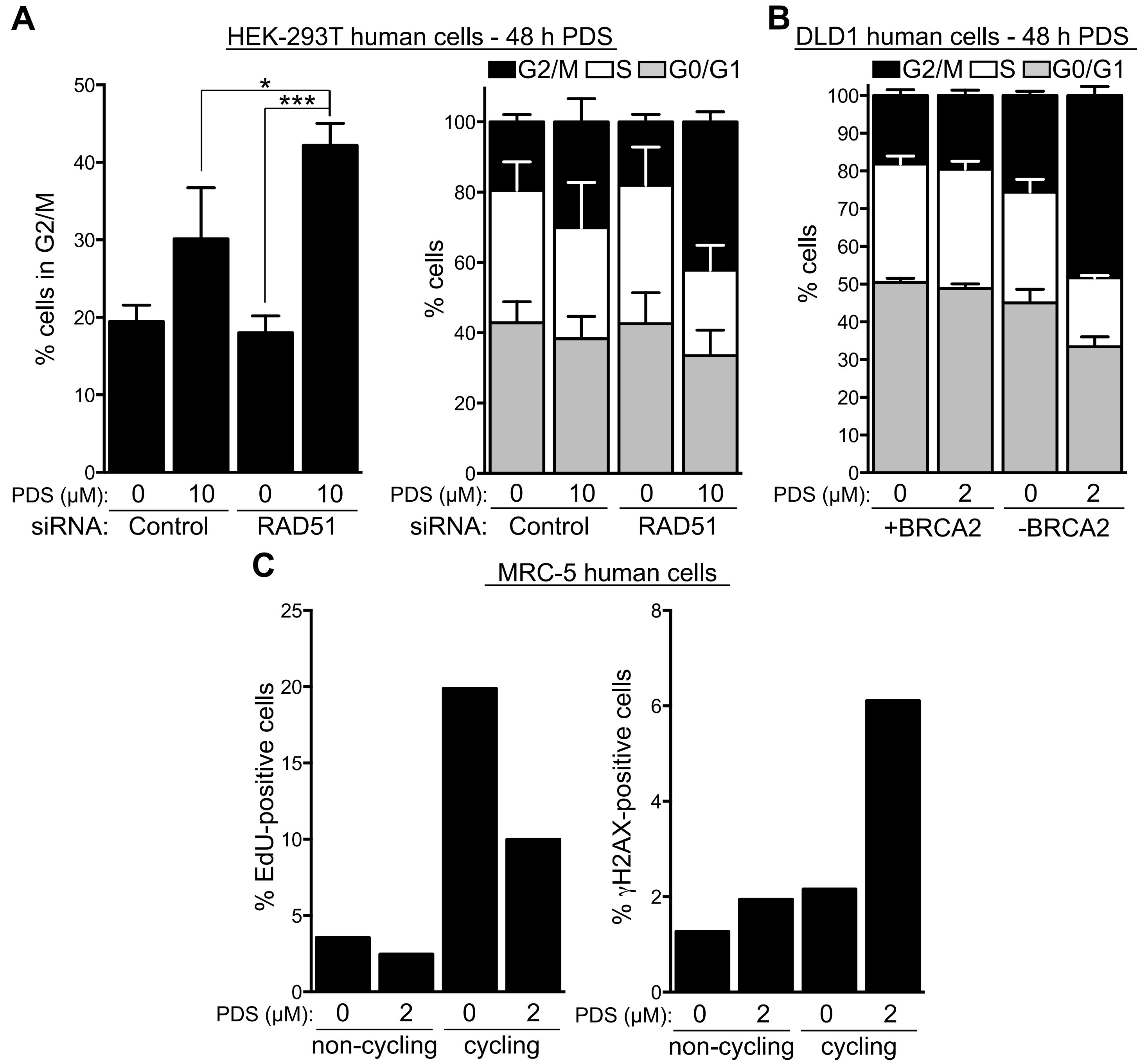

Figure S6 (related to Figure 6). (A) Cell cycle profiles of human HEK-293T cells transfected with control or RAD51 siRNA and treated with PDS for 48 h. Quantification of the percentage of cells in G2/M is also shown (*n*=3; error bars, SD). *P* values were calculated using an unpaired two-tailed *t*-test. *, $P \leq 0.05$; ***, $P \leq 0.001$. (B) Cell cycle profiles of human DLD1 cells, BRCA2-proficient (+BRCA2) and -deficient (-BRCA2), treated with PDS for 48 h (*n*=3; error bars, SD). Quantification of the percentage of cells in G2/M is shown in Figure 6B. (C) Human MRC-5 cells were arrested in G0 (non-cycling) by serum starvation or were allowed to re-enter the cell cycle (cycling) in the presence or absence of PDS. Replicating cells were labelled with EdU and DNA damage was detected with a FITC-conjugated γH2AX antibody. Cells were analysed by flow cytometry. Graphs are representative of two independent experiments.

Figure S7 (related to Figure 7)

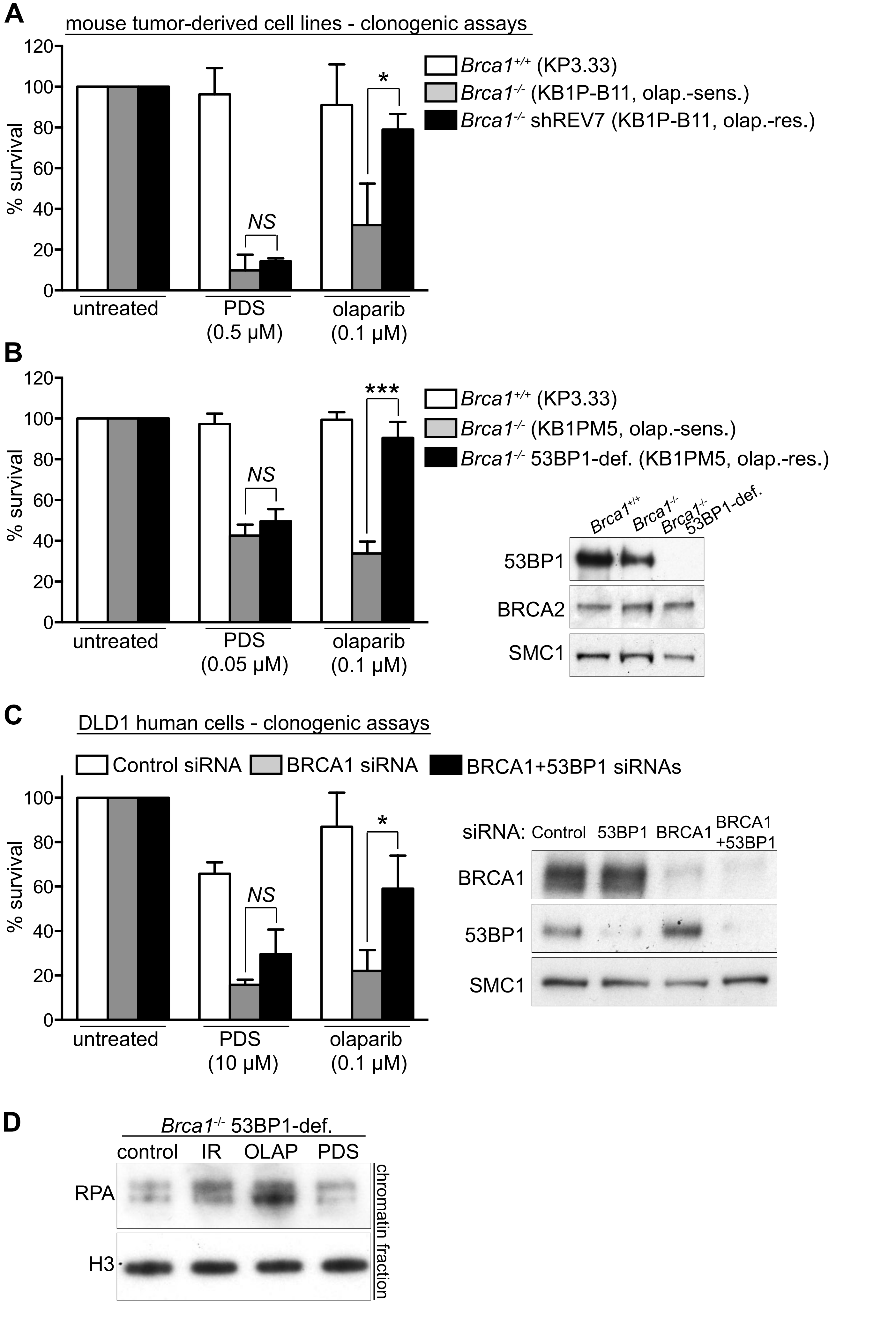

Figure S7 (related to Figure 7). (A and B) Colony survival assays of mouse mammary tumor-derived cell lines deficient in REV7 (A) or 53BP1 (B) . PDS or olaparib were added for 24 h. Graphs shown are representative of at least two independent experiments, each performed in triplicate. Error bars represent SD of triplicate values obtained from a single experiment. (B) Western blot analysis of cell extracts prepared from mouse mammary tumor-derived cell lines of indicated genotype. SMC1 was used as a loading control. (C) Human DLD1 cells were transfected with control, BRCA1 and/or 53BP1 siRNAs 24 h prior to seeding for colony survival assays performed as in (A). Graphs shown are representative of two independent experiments, each performed in triplicate. Error bars represent SD of triplicate values obtained from a single experiment. Cell extracts prepared 24 h after transfection were immunoblotted as indicated. SMC1 was used as a loading control. (D) Western blot analysis of chromatin-bound fractions of *Brca1*$^{-/-}$ 53BP1-deficient cells treated with 0.5 μM olaparib (OLAP) or PDS for 40 h, or irradiated with 10 Gy of ionizing radiation (IR), followed by recovery for 1 h. H3 was used as a loading control.

Supplemental Experimental Procedures

Cell lines and culture conditions

Human embryonic kidney HEK-293T cells, human non-small cell lung carcinoma H1299 cells and primary human fibroblasts MRC-5 (all from American Type Culture Collection), colorectal adenocarcinoma DLD1 cells (parental and *BRCA2*-mutated, Horizon Discovery; (Hucl et al., 2008)), as well as BRCA2-mutated hamster cells transduced with empty vector or BRCA2 (V-C8 and V-C8+BRCA2, respectively; (Kraakman-van der Zwet et al., 2002)) were cultivated in monolayers in DMEM medium (Sigma) supplemented with 10% fetal bovine serum (Life Technologies), penicillin and streptomycin (Sigma). H1299 cells expressing doxycycline (DOX)-inducible shRNAs were established using the 'all-in-one' system (Wiederschain et al., 2009). shRNAs targeting BRCA2 (GGG AAA CAC UCA GAU UAA A) or RAD51C (GAG AAU GUC UCA CAA AUA A) were cloned into $pLKO^{TetOn}$ and constructs were introduced into H1299 cells using lentiviral infection. Pooled cells showed efficient BRCA2 or RAD51C knockdown after eight days in the presence of 2 µg/ml DOX in DMEM medium supplemented with 10% tetracycline free fetal bovine serum (Clontech). A RAD51C shRNA-resistant H1299 cell line was generated by introducing the silent point mutations: 246T>C, 247C>T, 249C>A, 252A>T in a RAD51C-GFP expression construct in pEGFP-C1 vector. This construct and empty vector were introduced into H1299 cells expressing the DOX-inducible RAD51C shRNA using Lipofectamine™ 2000 (Life Technologies), following the manufacturer's protocol. To optimize the expression levels of RAD51C-GFP, single-cell clones were obtained by serial dilutions of the cell populations transfected with either RAD51C-GFP-expressing vector or empty vector control. GFP expression was assessed using an inverted microscope (DMI6000B; Leica) and a fluorescence imaging workstation.

Brca1$^{F/-}$, *Brca2*$^{F/-}$ and *Rad51c*$^{F/F}$ primary MEFs (Bouwman et al., 2010; Carlos et al., 2013; Kuznetsov et al., 2009) were isolated from day 13.5 embryos as previously described (Blasco et al., 1997), immortalized by overexpression of SV40 Large T (LT) antigen and cultivated in a low-oxygen (3%) incubator. For mitotic arrest, cells were treated with 0.2 µg/ml KaryoMAX® colcemid (Life Technologies) overnight. The following mouse mammary tumor-derived cell lines were used: *Brca1*$^{+/+}$ (KP3.33), *Brca1*$^{-/-}$ (KB1PM5, olaparib-sensitive) and *Brca1*$^{-/-}$ 53BP1-deficient (KB1PM5, olaparib-resistant; (Jaspers et al., 2013)); *Brca1*$^{-/-}$ (KB1P-B11, olaparib-sensitive), *Brca1*$^{-/-}$ shREV7 (KB1P-B11, olaparib-resistant; (Xu et al., 2015)); *Brca2*$^{-/-}$ (KB2P3.4, BRCA2-deficient), *Brca2*$^{-/-}$+B2iBAC (BRCA2-proficient; (Evers et al., 2010)). These cell lines were cultured at 37°C, 5% CO_2 and 3% O_2 in complete medium [DMEM/F-12, (Life Technologies) supplemented with 10% fetal bovine serum (Life Technologies), 1% penicillin-streptomycin (Sigma), 5 µg/ml insulin (Sigma), 5 ng/ml epidermal growth factor (Life Technologies), and 5 ng/ml cholera toxin (Sigma)].

MEF retroviral transduction

Retroviral transduction of cultured MEFs was performed as previously described (Palmero and Serrano, 2001). Briefly, HEK-293T packaging cells were grown to 70% confluency and transfected with pCL-Eco helper vector together with either pBabe alone, pBabe plus retroviral vector encoding 'Hit-and-run' Cre recombinase (Silver and Livingston, 2001) or shRNA against 53BP1 (Bouwman et al., 2010) using a standard calcium phosphate protocol. The medium was replaced 24 h after transfection. Recipient MEFs were plated and infected 24 h later with the retroviral supernatants produced by the HEK-293T cells. Additional infections were performed after 24 and 32 h. Twenty-four hours after the last infection, cells were incubated in fresh medium containing 3 µg/ml puromycin for 48 h.

In vivo experiment

CD-1 male nude (nu/nu) mice, 6 weeks old and weighing 26-28 g were purchased from Charles River Laboratories (Calco, Italy). All animal procedures were in compliance with the national and international directives (D.L. March 4, 2014, no. 26; directive 2010/63/EU of the European Parliament and of the council; Guide for the Care and Use of Laboratory Animals, United States National Research Council, 2011). Mice were injected intramuscularly into the hind leg muscles with $5x10^6$ DLD1 BRCA2-proficient or -deficient cells per mouse. When a tumor mass of about 250 mg was evident in BRCA2-proficient (three days after cell injection) and -deficient (six days after cell injection) xenografts, the treatment was initiated. RHPS4 (10 mg/kg) was administered intravenously for ten consecutive days. Tumors were measured three times a week in two dimensions by a caliper and tumor weight was calculated using the formula $a \times b^2/2$, where a and b are the long and short sizes of the tumor, respectively. Each experimental group included eight mice. Therapeutic efficacy of treatment

was assessed by percent tumor weight inhibition (TWI%) calculated as [1-(mean tumor weight of treated mice/ mean tumor weight of controls)]x 100).

Plasmid-based replication assay
The construct containing the telomere sequence $(TTAGGG)_7$ used in Figures 1 and S1B was generated as in Szüts et al. (2008). A plasmid containing the mutant $(TTACGC)_7$ sequence, which abrogates the G4-forming potential of the telomeric sequence (Figure S1C), was made using QuikChange II XL Site-Directed Mutagenesis Kit (Agilent Technologies) following the manufacturer's protocol. H1299 cells expressing DOX-inducible BRCA2 or RAD51C shRNAs were grown for eight days in the presence or absence of DOX and plated at 90% confluency. The following day, Lipofectamine™ 2000 (Life Technologies) was used to co-transfect 10 µg of $pQ1^{Amp}$ plasmid containing $(TTAGGG)_7$ or empty vector together with control $pQ2^{Kan}$ plasmid. Plasmid DNA was extracted 48 h after transfection using a simplified Hirt protocol. Briefly, cells were harvested by trypsinization, washed in PBS, resuspended in buffer P1 from a plasmid miniprep kit (Qiagen), lysed in buffer P2 and neutralized in buffer N3. Plasmid DNA was recovered from the resulting supernatant using glycogen and isopropanol precipitation. Dried pellets were dissolved in DpnI digest mix (containing 10 U DpnI) and incubated at 37°C for 30 min to degrade parental DNA. Plasmid DNA was recovered using isopropanol precipitation, dried and dissolved in 5 µl dH_2O. Recovered DNA was used to electroporate 20 µl E-shot electro-competent cells (Life Technologies) at 200 Ω, 0.25 µF and 1.8 kV in a Bio-Rad E. coli pulser™ (165-2104) and 500 µl SOC was added immediately. Cells were allowed to recover on ice for 5 min and subsequently incubated at 37°C for 1 h. Typically 200 µl cells were plated on kanamycin and 20 µl on ampicillin plates. Colony numbers were quantified and plasmid replication efficiency (%) was determined by normalizing the Amp^R colonies to the internal Kan^R control, followed by expressing the replication efficiency of $pQ1^{Amp}$ plasmid containing $(TTAGGG)_7$ relative to the replication efficiency of the empty vector.

RNAi
1.5×10^6 cells were transfected with 40 nM siRNA per plate by reverse transfection in 10-cm plates. After 24-h incubation, depletion was evident as determined by immunoblotting. When using RAD51 siRNA, cells were transfected again three and six days after the first transfection as above. The 53BP1 siRNA CAG GAC AGT CTT TCC ACG AAT, BRCA1 siRNA CAG CAG TTT ATT ACT CAC TAA and RAD51 siRNA CUU UGG CCC ACA ACC CAU with two-base deoxynucleotide overhangs were obtained from Dharmacon, RAD51 esiRNA from Sigma and AllStars negative control siRNA from Qiagen.

Clonogenic assays
Cells were plated at densities between 100 and 1,000 cells per well in 6-well plates and drug treatment was initiated after cells had adhered. Following 24-h incubation with the drug, fresh media without the drug were added for 6-14 days. Colonies were stained with 0.5% crystal violet (Sigma) in 50% methanol, 20% ethanol in dH_2O. Cell survival was expressed relative to untreated cells of the same cell line, thus accounting for any differences in viability caused by HR deficiency.

Proliferation assays
To determine population doublings, resazurin-based readouts of cell viability were taken after cells had adhered (day 0) and every 24 h for four days.

MRC-5 cell cycle arrest
MRC-5 cells were cultured in media containing 0.1% fetal bovine serum for four days to induce G0 arrest. To enable re-entry into the cell cycle, 10% fetal bovine serum was added to the media for 48 h.

EdU incorporation and gH2AX labeling
To label replicated DNA, cells were incubated with 10 µM EdU for 45 min and incorporated EdU was detected using the Click-iT EdU Alexa Fluor 647 Flow Cytometry Assay Kit (Molecular Probes) according to manufacturer's instructions. To stain for γH2AX, cells were incubated with an anti-γH2AX (Ser139) FITC conjugate (1:50, Millipore) for 1 h at room temperature. Cells were resuspended in PBS containing 20 µg/ml propidium iodide and 10 µg/ml RNase A before samples were processed using flow cytometry (BD FACSCalibur, BD Biosciences) and data was analyzed using FlowJo software.

Alkaline single-cell gel electrophoresis comet assay

2 x 10^5 cells were embedded in 1% low-melting agarose in PBS on a microscope slide. Subsequently, the cells were lysed in buffer containing 2.5 M NaCl, 100 mM EDTA, 10 mM Tris-HCl pH 10.5, 1% DMSO and 1% Triton X-100 for 1 h at 4°C. To denature the DNA, the slides were incubated in cold electrophoresis buffer (300 mM NaOH, 1 mM EDTA, 1% DMSO, pH >13) for 30 min in the dark. Following electrophoresis at 25 V and 300 mA for 25 min, the DNA was neutralized with 0.5 M Tris-HCl pH 8.0 and stained with SYBR Gold (Invitrogen).

Immunofluorescence (IF)

Cells were washed in PBS, swollen in hypotonic solution (85.5 mM NaCl and 5 mM $MgCl_2$) for 5 min, fixed with 4% paraformaldehyde for 10 min at room temperature (and with 100% ice-cold methanol for RPA) and permeabilized by adding 0.03% SDS to the fixative. After blocking with blocking buffer (1% goat serum, 0.3% BSA, 0.005% Triton X-100 in PBS), cells were incubated with primary antibody diluted in blocking buffer overnight at room temperature. Then, they were washed again and incubated with fluorochrome-conjugated secondary antibodies for 1 h at room temperature. Dried coverslips were mounted on microscope slides using the ProLong Antifade kit (Life Technologies) supplemented with 2 μg/ml DAPI.

Preparation of metaphase spreads

Mitotic cells were collected by mitotic shake-off and swollen in hypotonic buffer (0.03 M sodium acetate) at 37°C for 25 min. For telomere FISH, cells were fixed in a freshly prepared 3:1 mix of methanol:glacial acetic acid. Nuclear preparations were dropped onto slides pre-soaked in 45% acetic acid and left to dry overnight.

Telomeric FISH

Telomeric probe mix containing: 10 mM Tris pH 7.5, 2.175 mM $MgCl_2$, 0.08 mM citric acid, 7.2 mM Na_2HPO_4 pH7.0, 70% deionized formamide (Chemicon Int.), 0.5 μg/ml Cy3-conjugated PNA $(CCCTAA)_3$ telomeric probe (Applied Biosystems) and 0.25% blocking reagent (100 mM maleic acid and 50 mM NaCl pH7.5 (Roche) in dH_2O) was dropped onto each slide and sealed with a coverslip. Following denaturation on a hot plate for 3 min at 80°C, the slides were incubated at room temperature for 1.5 h in a dark humidified chamber. After washing twice in formamide (70% formamide (Fluka), 10 mM Tris, 0.1% BSA (Fluka)), three times in PBS and once in dH_2O, the slides were left to dry at room temperature. Slides were then mounted using ProLong Antifade (Life Technologies) supplemented with 2 μg/ml 4,6-diamidino-2-phenylindole (DAPI). Mitotic chromosomes were viewed with a Leica DMI6000B inverted microscope and fluorescence imaging workstation equipped with a HCX PL APO 100x/1.4-0.7 oil objective. Images were acquired using a Leica DFC350 FX R2 digital camera and LAS-AF software (Leica). Brightness levels and contrast adjustments were applied to the whole image using Photoshop CS3 (Adobe).

Telomere chromosome orientation FISH (CO-FISH)

After washing in PBS for 10 min, the slides were treated with 0.5 mg/ mL RNase A (Sigma Aldrich) in PBS for 10 min at 37°C. Following washes in PBS and 2 x saline-sodium citrate (SSC), the DNA was stained with 0.5 mg/ml Hoechst 33258 (Sigma Aldrich) in 2xSSC for 15 min. In order to introduce DNA breaks, slides were exposed to ultraviolet (UV; 365 nm) light for 25 min. Following PBS washes, the DNA was digested with 3 U/mL of Exonuclease III (Promega). The slides were washed again with PBS and then dehydrated with sequential washes of 70, 90 and 100% ethanol. When dried, the first telomeric probe mix (same as described for IF-FISH) containing 0.5 μg/ml Cy3-conjugated PNA $(TTAGGG)_3$ telomeric probe (Applied Biosystems) was added to the slides. DNA was denatured on a hot plate at 80°C for 3 min, then slides were incubated for 1.5 h at room temperature and washed twice with formamide wash, as described for IF-FISH. Next the slides were washed with PBS, dehydrated with ethanol and after air drying, were incubated with the second telomeric probe mix containing 0.5 μg/ml FITC-conjugated PNA $(CCCTAA)_3$ telomeric probe (Applied Biosystems) for 1.5 h at room temperature. Following washes with formamide wash and PBS and dehydration with ethanol, slides were dried and mounted using Vectashield (Vector Laboratories) supplemented with 2 μg/ml DAPI.

DNA fiber assay

DNA was labeled with 25 μM CldU and 250 μM IdU for 30 min each. The reaction was terminated by addition of ice-cold PBS. After cell lysis, DNA was spread on glass slides, fixed in methanol/acetic acid, denatured with HCl, blocked with 2% BSA and stained with anti-rat anti-CldU (1:500, Abcam) and mouse anti-IdU (1:100, Beckton Dickinson) antibodies. Anti-rat Cy3 (1:300, Jackson

ImmunoResearch) and anti-mouse Alexa 488 (1:300, Molecular Probes) were used as secondary antibodies. Images were acquired as described for FISH and analyzed using ImageJ software (National Healthcare Institute, USA).

Immunoblotting and cell fractionation

Cells were harvested by trypsinization, washed with cold PBS, re-suspended in SDS-PAGE loading buffer, sonicated and boiled at 70°C for 10 min to prepare whole cell extracts. Fractionation of human cells was performed as described in Rodrigue et al. (2006). Chromatin fractions of mouse cells were prepared as described in Mendez and Stillman (2000). Equal amounts of protein (50-100 µg) were analysed by gel electrophoresis followed by Western blotting. NuPAGE-Novex 10% Bis-Tris and 3-8% Tris-Acetate gels (Life Technologies) were run according to manufacturer's instructions.

Antibodies

The following antibodies were used for immunoblotting: rabbit polyclonal antisera raised against 53BP1 (NB100-304, Novus), phosphorylated ATM Ser1981 (10H11, Cell Signaling), ATM (MAT3-4G10/8, Sigma-Aldrich), phosphorylated CHK1 Ser317&Ser345 (2344&2341, Cell Signaling), phosphorylated CHK2 Thr68 (Cell Signaling), H2AX (DR1016, Calbiochem), H3 (H3C, a gift from A. Verreault, University of Montreal), phosphorylated KAP1 Ser824 (A300-767A, Bethyl Laboratories), KAP1 (A300-274A, Bethyl Laboratories), cleaved PARP1 Asp214 (9541, Cell Signaling), PARP1 (46D11, Cell Signaling), RAD51 (H92, Santa Cruz), phosphorylated RPA32 Ser4&Ser8 (A300-245A, Bethyl Laboratories), SMC1 (BL308, Bethyl Laboratories), mouse monoclonal antibodies raised against RAD51C (2H11, Cancer Research UK Monoclonal Antibody Service), BRCA2 (OP95, Calbiochem), CHK1 (sc-8408, Santa Cruz), CHK2 (clone 7, Merck Millipore), GAPDH (6C5, Novus Biologicals), α-tubulin (TAT1, Cancer Research UK Monoclonal Antibody Service), phosphorylated histone H2AX Ser139 (clone JBW301, Merck Millipore), a rat monoclonal antibody raised against RPA2 (a gift from H. P. Nasheuer, NUI Galway) and a sheep polyclonal antibody raised against mouse BRCA2 ((Min et al., 2012); a gift from H. Lee, Seoul National University). Additional antibodies used for immunofluorescence detection were: rabbit polyclonal antiserum raised against RAD51 (H92, Santa Cruz), rabbit polyclonal antiserum raised against RPA (SWE34, a gift from Steve West, Cancer Research UK Clare Hall Laboratories) and mouse monoclonal antibody raised against phosphorylated histone H2AX Ser139 (clone JBW301, Merck Millipore).

References

Blasco, M.A., Lee, H.W., Hande, P.M., Samper, E., Lansdorp, P.M., DePinho, R.A., and Greider, C.W. (1997). Telomere shortening and tumor formation by mouse cells lacking telomerase RNA. Cell *91*, 25-34.

Bouwman, P., Aly, A., Escandell, J.M., Pieterse, M., Bartkova, J., van der Gulden, H., Hiddingh, S., Thanasoula, M., Kulkarni, A., Yang, Q*., et al.* (2010). 53BP1 loss rescues BRCA1 deficiency and is associated with triple-negative and BRCA-mutated breast cancers. Nat Struct Mol Biol *17*, 688-695.

Carlos, A.R., Escandell, J.M., Kotsantis, P., Suwaki, N., Bouwman, P., Badie, S., Folio, C., Benitez, J., Gomez-Lopez, G., Pisano, D.G*., et al.* (2013). ARF triggers senescence in Brca2-deficient cells by altering the spectrum of p53 transcriptional targets. Nature communications *4*, 2697.

Evers, B., Schut, E., van der Burg, E., Braumuller, T.M., Egan, D.A., Holstege, H., Edser, P., Adams, D.J., Wade-Martins, R., Bouwman, P*., et al.* (2010). A high-throughput pharmaceutical screen identifies compounds with specific toxicity against BRCA2-deficient tumors. Clinical cancer research : an official journal of the American Association for Cancer Research *16*, 99-108.

Hucl, T., Rago, C., Gallmeier, E., Brody, J.R., Gorospe, M., and Kern, S.E. (2008). A syngeneic variance library for functional annotation of human variation: application to BRCA2. Cancer Res *68*, 5023-5030.

Jaspers, J.E., Kersbergen, A., Boon, U., Sol, W., van Deemter, L., Zander, S.A., Drost, R., Wientjens, E., Ji, J., Aly, A*., et al.* (2013). Loss of 53BP1 causes PARP inhibitor resistance in Brca1-mutated mouse mammary tumors. Cancer discovery *3*, 68-81.

Kraakman-van der Zwet, M., Overkamp, W.J.I., van Lange, R.E.E., Essers, J., van Duijn-Goedhart, A., Wiggers, I., Swaminathan, S., van Buul, P.P.W., Errami, A., Tan, R.T.L*., et al.* (2002). BRCA2 (XRCC11) deficiency results in radioresistant DNA synthesis and a higher frequency of spontaneous deletions. Mol. Cell Biol. *22*, 669-679.

Kuznetsov, S., Haines, D.C., Martin, B.K., and Sharan, S.K. (2009). Loss of RAD51C leads to embryonic lethality and modulation of Trp53-dependent tumorigenesis in mice. Cancer Res. *69*, 863-872.

Mendez, J., and Stillman, B. (2000). Chromatin association of human origin recognition complex, cdc6, and minichromosome maintenance proteins during th ecell cycle: assembly of prereplication complexes in late mitosis. Mol. Cell Biol. *20*, 8602-8612.

Min, J., Choi, E.S., Hwang, K., Kim, J., Sampath, S., Venkitaraman, A.R., and Lee, H. (2012). The breast cancer susceptibility gene BRCA2 is required for the maintenance of telomere homeostasis. J. Biol. Chem. *287*, 5091-5101.

Palmero, I., and Serrano, M. (2001). Induction of senescence by oncogenic Ras Methods in Enzymology *333*, 247-256.

Rodrigue, A., Lafrance, M., Gauthier, M.-C., McDonald, D., Hendzel, M., West, S.C., Jasin, M., and Masson, J.-Y. (2006). Interplay between human DNA repair proteins at a unique double-strand break in vivo The EMBO journal *25*, 222-231.

Silver, D.P., and Livingston, D.M. (2001). Self-excising retroviral vectors encoding the Cre recombinase overcome Cre-mediated cellular toxicity. Mol. Cell *8*, 233-243.

Szüts, D., Marcus, A.P., Himoto, M., Iwai, S., and Sale, J.E. (2008). REV1 restrains DNA polymerase zeta to ensure frame fidelity during translesion synthesis of UV photoproducts in vivo. Nucleic Acids Res. *36*, 6767-6780.

Wiederschain, D., Wee, S., Chen, L., Loo, A., Yang, G., Huang, A., Chen, Y., Caponigro, G., Yao, Y.M., Lengauer, C.*, et al.* (2009). Single-vector inducible lentiviral RNAi system for oncology target validation. Cell Cycle *8*, 498-504.

Xu, G., Chapman, J.R., Brandsma, I., Yuan, J., Mistrik, M., Bouwman, P., Bartkova, J., Gogola, E., Warmerdam, D., Barazas, M.*, et al.* (2015). REV7 counteracts DNA double-strand break resection and affects PARP inhibition. Nature *521*, 541-544.
