# Supplementary material for: Targeting BRCA1 and BRCA2 Deficiencies with G-Quadruplex-Interacting Compounds
Source: Mol Cell. 2016 Feb 4;61(3):449–60. doi: 10.1016/j.molcel.2015.12.004 (PMC4747901; doi:10.1016/j.molcel.2015.12.004)
Supplement: Document S2. Article plus Supplemental Information [file mmc2.pdf]

Molecular Cell

Article

Targeting BRCA1 and BRCA2 Deficiencies with G-Quadruplex-Interacting Compounds

Graphical Abstract

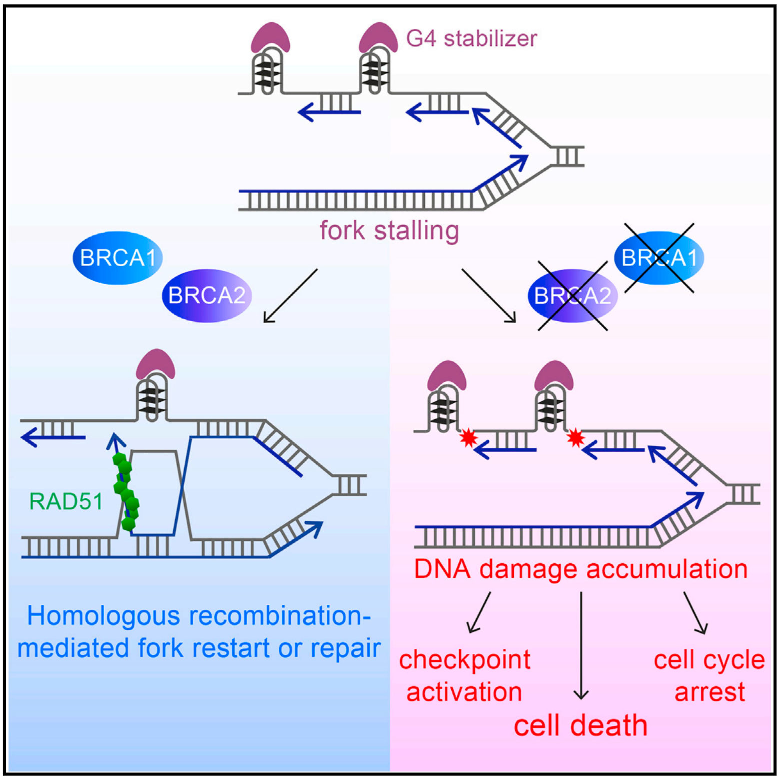

Authors

Jutta Zimmer, Eliana M.C. Tacconi, Cecilia Folio, ..., Annamaria Biroccio, Julian E. Sale, Madalena Tarsounas

Correspondence

madalena.tarsounas@oncology.ox.ac.uk

In Brief

Zimmer et al. discovered that homologous recombination activities of BRCA1 and BRCA2 facilitate replication of genomic regions with G-quadruplex-forming potential, including telomeres, and suppress genomic instability stemming from inefficient replication of these sites. G4-stabilizing compounds are toxic to BRCA1- and BRCA2-deficient cells, highlighting their therapeutic potential in targeting BRCA deficiency.

Highlights

- G4 formation on the G-rich strand drives telomere fragility in HR-deficient cells
- G4-stabilizing compounds reduce viability of cells lacking BRCA1, BRCA2, or RAD51
- G4 toxicity stems from excessive replication stress and DNA damage accumulation
- Olaparib-resistant, BRCA-defective cells are sensitive to G4-stabilizing compounds

Zimmer et al., 2016, Molecular Cell *61*, 449–460
February 4, 2016 ©2016 The Authors
http://dx.doi.org/10.1016/j.molcel.2015.12.004

Molecular Cell
Article

Targeting BRCA1 and BRCA2 Deficiencies with G-Quadruplex-Interacting Compounds

Jutta Zimmer,[1] Eliana M.C. Tacconi,[1] Cecilia Folio,[1] Sophie Badie,[1] Manuela Porru,[2] Kerstin Klare,[1,10] Manuela Tumiati,[3] Enni Markkanen,[4,11] Swagata Halder,[5] Anderson Ryan,[6] Stephen P. Jackson,[7,8] Kristijan Ramadan,[5] Sergey G. Kuznetsov,[3] Annamaria Biroccio,[2] Julian E. Sale,[9] and Madalena Tarsounas[1,*]
[1]Genome Stability and Tumourigenesis Group, CRUK/MRC Oxford Institute for Radiation Oncology, Department of Oncology, University of Oxford, Old Road Campus Research Building, Oxford OX3 7DQ, UK
[2]Area of Translational Research, Regina Elena National Cancer Institute, 00144 Rome, Italy
[3]Institute for Molecular Medicine Finland (FIMM), University of Helsinki, P.O. Box 20, FIN-00014 Helsinki, Finland
[4]Biochemistry and Regulation of DNA Repair Group, CRUK/MRC Oxford Institute for Radiation Oncology, Department of Oncology, University of Oxford, Old Road Campus Research Building, Oxford OX3 7DQ, UK
[5]DNA Damage and Repair Group, CRUK/MRC Oxford Institute for Radiation Oncology, Department of Oncology, University of Oxford, Old Road Campus Research Building, Oxford OX3 7DQ, UK
[6]Lung Cancer Translational Science Research Group, CRUK/MRC Oxford Institute for Radiation Oncology, Department of Oncology, University of Oxford, Old Road Campus Research Building, Oxford OX3 7DQ, UK
[7]The Gurdon Institute, CRUK Laboratories, University of Cambridge, Tennis Court Road, Cambridge CB2 1QN, UK
[8]The Sanger Institute, Hinxton, Cambridge CB10 1SA, UK
[9]Medical Research Council Laboratory of Molecular Biology, Francis Crick Avenue, Cambridge CB2 0QH, UK
[10]Present address: Department of Mechanistic Cell Biology, Max Planck Institute of Molecular Physiology, 44227 Dortmund, Germany
[11]Present address: Institute of Pharmacology and Toxicology, Vetsuisse Faculty, University of Zürich, 8006 Zürich, Switzerland
*Correspondence: madalena.tarsounas@oncology.ox.ac.uk
http://dx.doi.org/10.1016/j.molcel.2015.12.004
This is an open access article under the CC BY license (http://creativecommons.org/licenses/by/4.0/).

SUMMARY

G-quadruplex (G4)-forming genomic sequences, including telomeres, represent natural replication fork barriers. Stalled replication forks can be stabilized and restarted by homologous recombination (HR), which also repairs DNA double-strand breaks (DSBs) arising at collapsed forks. We have previously shown that HR facilitates telomere replication. Here, we demonstrate that the replication efficiency of guanine-rich (G-rich) telomeric repeats is decreased significantly in cells lacking HR. Treatment with the G4-stabilizing compound pyridostatin (PDS) increases telomere fragility in BRCA2-deficient cells, suggesting that G4 formation drives telomere instability. Remarkably, PDS reduces proliferation of HR-defective cells by inducing DSB accumulation, checkpoint activation, and deregulated G2/M progression and by enhancing the replication defect intrinsic to HR deficiency. PDS toxicity extends to HR-defective cells that have acquired olaparib resistance through loss of 53BP1 or REV7. Altogether, these results highlight the therapeutic potential of G4-stabilizing drugs to selectively eliminate HR-compromised cells and tumors, including those resistant to PARP inhibition.

INTRODUCTION

Genomic instability is a hallmark of cancer caused by failure of normal DNA replication and/or repair mechanisms (Halazonetis et al., 2008; Negrini et al., 2010). During replication, the enzymatic activities of DNA polymerases, helicases, and nucleases act in concert to assemble the active replication fork and to achieve high-fidelity duplication of the genome. Damaged DNA, secondary DNA structures, and DNA-protein complexes obstruct progression of replication forks, leading to fork stalling or, in more severe cases, to irreversible fork collapse and DNA breakage. Several mechanisms have evolved to overcome barriers to replication-fork movement, one of which exploits the HR DNA repair machinery. HR factors act to stabilize stalled replication forks by preventing their nucleolytic degradation (Hashimoto et al., 2010; Schlacher et al., 2011) to restart arrested forks (Lambert et al., 2010) and to repair double-strand breaks (DSBs) arising from disintegrated forks (Aze et al., 2013).

The tumor suppressor BRCA2 is a key component of the HR pathway of DSB repair. BRCA2 promotes recombination reactions by loading the RAD51 recombinase onto single-stranded DNA (ssDNA) in concert with the family of proteins known as the RAD51 paralogs, of which RAD51C is a member (Suwaki et al., 2011). RAD51-coated ssDNA invades an intact, homologous duplex DNA molecule, most commonly a sister chromatid, which becomes the template for accurate DSB repair.

In vitro, G-rich ssDNA can adopt secondary structures known as G4s under physiological-like conditions (Lipps and Rhodes, 2009). G4s consist of stacks of two or more G-quartets formed by four guanines via Hoogsteen base pairing stabilized by a monovalent cation. While in silico analyses have identified more than 300,000 sites with G4-forming potential in the human genome (Huppert and Balasubramanian, 2005), more recent G4-seq approaches enabled detection of more than 700,000 G4 structures genome-wide (Chambers et al., 2015). The first in vitro visualization of a G4 structure was based on diffraction

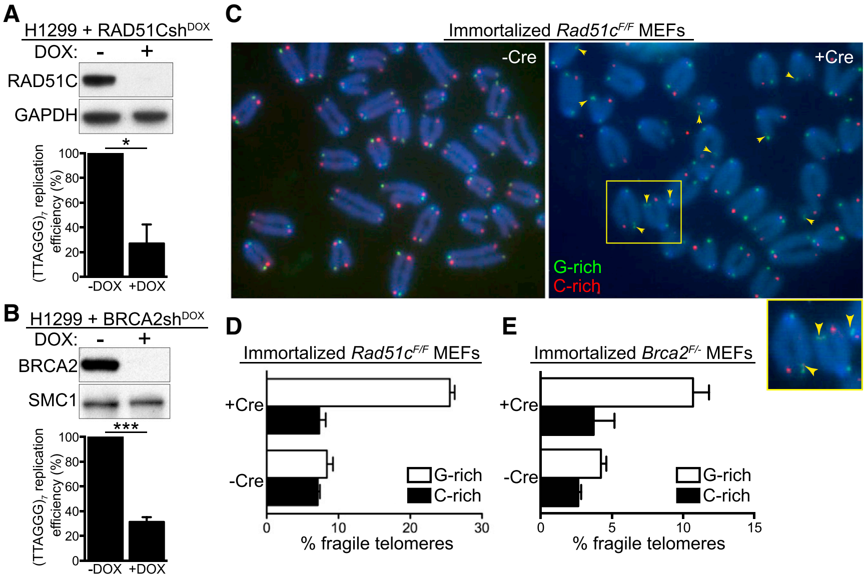

Figure 1. RAD51C and BRCA2 Prevent Lagging-Strand Telomere Fragility

(A and B) Replication efficiency of a plasmid containing $(TTAGGG)_7$ in H1299 cells expressing doxycycline (DOX)-inducible RAD51C (A) or BRCA2 (B) shRNAs is shown relative to the replication efficiency of the empty vector (n = 3 for RAD51Csh^{DOX}; n = 4 for BRCA2sh^{DOX}; error bars, SEM). p values were calculated using a one-sample t test (*$p \leq 0.05$ and ***$p \leq 0.001$). Cell extracts prepared at the time of plasmid transfection were immunoblotted as indicated. GAPDH and SMC1 were used as loading controls.

(C) CO-FISH detection of lagging (G-rich, green) and leading (C-rich, red) telomeric strands in immortalized *Rad51c*$^{F/F}$ MEFs treated with Cre (+Cre) and control (−Cre) retroviruses. Enlarged inset shows the area marked with the yellow rectangle. Arrows mark lagging-strand fragile telomeres.

(D and E) Quantification of fragile telomeres in immortalized *Rad51c*$^{F/F}$ (D) and *Brca2*$^{F/-}$ (E) MEFs. Approximately 1,000 telomeres were scored per condition per replica (n = 2; error bars, SD). See also Figure S1.

patterns of a guanylic acid solution (Gellert et al., 1962), while evidence that G4s assemble in vivo initially came from immunostaining of *Stylonychia* macronuclei with antibodies raised against G4 structures with telomere sequences (Schaffitzel et al., 2001). This study demonstrated that telomeres adopt a G4 configuration in vivo. G4 structures have been subsequently detected with several other structure-specific antibodies (Biffi et al., 2013; Henderson et al., 2014; Schaffitzel et al., 2001) and interacting small molecules (Lam et al., 2013; Müller et al., 2010; Rodriguez et al., 2012). Importantly, telomeric G-rich DNA sequences have a high propensity to adopt G4 configurations (Parkinson et al., 2002). Telomeres, repetitive DNA sequences bound by the protein complex shelterin, protect chromosome ends from degradation and fusion. Telomeric G4s can interfere with telomere replication, leading to fragile, shorter telomeres. Supporting this concept, treatment with G4-stabilizing compounds induces telomere dysfunction (Gomez et al., 2006; Rodriguez et al., 2008; Salvati et al., 2007; Tahara et al., 2006).

During DNA replication, G4s are thought to assemble spontaneously on G-rich ssDNA displaced during fork movement. Due to their thermodynamic stability, G4s cause uncoupling of replisome components and fork stalling, which have the potential to trigger genomic instability. Helicases such as FANCJ, PIF1, RECQ, BLM, and WRN, the chromatin remodeler ATRX, and the REV1 translesion polymerase act to dismantle G4s in vitro. Several lines of evidence support a similar function in vivo for these factors, essential to preserve genome stability during DNA replication (Murat and Balasubramanian, 2014). Conversely, G4 configurations can be stabilized by specific ligands that exhibit higher binding specificity for G4s over duplex DNA, with the G4-interacting compound PDS being one example (Chambers et al., 2015). In mammalian cells, G4 stabilization by PDS results in dissociation of shelterin components from telomeres (Rodriguez et al., 2008). More recently, PDS was demonstrated to trigger replication- and transcription-associated DNA damage at genomic sites with predicted G4-forming potential (Lam et al., 2013; Rodriguez et al., 2012). These findings highlight the deleterious consequences of persistent G4s for telomere and genome integrity.

HR factors, including BRCA2 and RAD51, are required to facilitate telomere replication and to prevent telomere shortening (Badie et al., 2010). It remained unclear, however, whether assembly of telomeric G4s could contribute to the telomere replication defect of HR-deficient cells. In this work, we demonstrate that telomere fragility in cells lacking HR repair is enhanced by PDS treatment. Importantly, G4-stabilizing compounds, including PDS, decrease the viability of BRCA1-, BRCA2-, or RAD51-deficient cells, which is associated with elevated levels of DNA damage and replication stress. We suggest that in the context of HR deficiency, persistent G4 structures exacerbate the cell-intrinsic challenges that arise during replication of regions with G4-forming potential, thus eliciting checkpoint activation, G2/M cell-cycle arrest, and cell death. This work is therefore highly relevant to the search for treatments that selectively kill tumor cells whose capacity for HR-mediated repair has been compromised.

RESULTS

BRCA2 and RAD51C Are Required for G-Rich Strand Telomere Replication

Abrogation of key HR activities elicits telomere fragility (Badie et al., 2010) suggestive of a role for HR in telomere replication. To further investigate this concept, we used a plasmid-based replication assay (Szüts et al., 2008) in H1299 cells harboring inducible small hairpin RNA (shRNA) against RAD51C or BRCA2. Doxycycline addition induced efficient depletion of both proteins, as determined by western blotting (Figures 1A and 1B). The replication efficiency of a plasmid containing an array of seven telomeric repeats $(TTAGGG)_7$ was significantly lower in RAD51C- or BRCA2-deficient cells compared to control

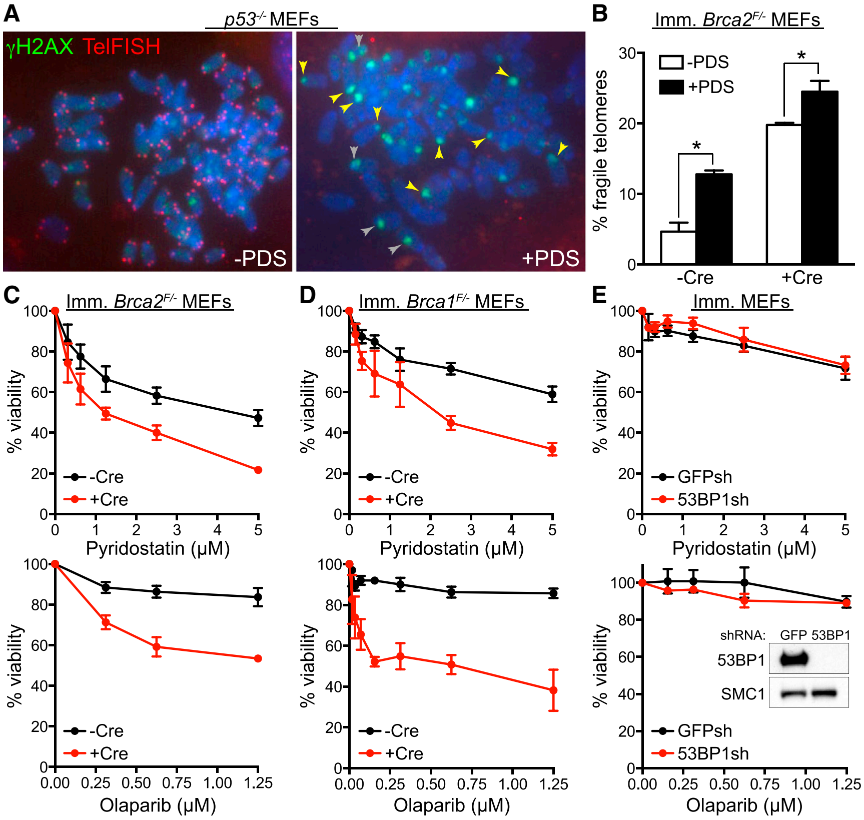

Figure 2. Effect of the G4-Interacting Compound PDS on Telomere Fragility and Viability of *Brca*-Deficient MEFs

(A) Mitotic chromosome spreads of *p53*$^{-/-}$ MEFs grown in the presence (+PDS) or absence (−PDS) of 5 μM PDS for 48 hr. Preparations were fixed and stained with anti-γH2AX monoclonal antibody (green). Telomeres were visualized with a Cy3-conjugated $(CCCTAA)_6$-PNA probe (red), using identical exposure conditions for untreated and PDS-treated cells. DNA was counterstained with DAPI (blue).

(B) Quantification of fragile telomeres visualized by FISH on metaphase chromosomes from *Brca2*$^{F/-}$ MEFs treated with Cre (+Cre) and control (−Cre) retroviruses incubated with 5 μM PDS for 40 hr (n = 2; > 1,500 long-arm telomeres were scored per condition per replica; error bars, SD). p values were calculated using an unpaired two-tailed t test (*p ≤ 0.05).

(C) Dose-dependent viability assays of *Brca2*$^{F/-}$ MEFs treated with Cre (+Cre) and control (−Cre) retroviruses exposed to PDS or olaparib at the indicated concentrations.

(D) Dose-dependent viability assays of *Brca1*$^{F/-}$ MEFs treated as in (C).

(E) Dose-dependent viability assays of immortalized (imm.) MEFs treated as in (C) with retroviruses encoding shRNA against GFP or 53BP1 (Bouwman et al., 2010). Cell extracts were immunoblotted as indicated. SMC1 was used as a loading control. See also Figures S1 and S2. Graphs shown are representative of at least two independent experiments, each performed in triplicate. Error bars represent SD of triplicate values obtained from a single experiment.

cells (Figures 1A and 1B). RAD51C inhibition did not affect cell proliferation rate (Figure S1A, available online). Full-length human RAD51C rescued the telomere replication defect completely, indicating specificity of the shRNA for its target (Figure S1B). Importantly, replication of a plasmid containing a $(TTACGC)_7$ sequence, with two G-to-C substitutions in the telomere repeat, which abrogate the G4-forming potential of the sequence, was not affected by loss of RAD51C expression (Figure S1C). Collectively, these data suggest that assembly of G4 secondary structures on the telomere-containing plasmid underlines its inefficient replication in BRCA2- or RAD51C-depleted cells.

We previously reported that *Brca2* or *Rad51c* deletion in mouse embryonic fibroblasts (MEFs) leads to increased levels of multiple telomeric fluorescence in situ hybridization (FISH) signals (Badie et al., 2010), indicative of telomere fragility. To examine the specificity of the fragile telomere phenotype to the leading or lagging-strand template, chromosome orientation FISH (CO-FISH) assays were performed in immortalized *Brca2*$^{F/-}$ or *Rad51c*$^{F/F}$ MEFs, in which gene deletion was induced with "hit-and-run" Cre recombinase. The telomeric G-rich strand showed a clear propensity for FISH signal fragmentation (Figure 1C, green). Quantification of fragmented telomeric CO-FISH signals further demonstrated the bias toward fragility of the G-rich telomeric strand in Cre-treated *Brca2*$^{F/-}$ and *Rad51c*$^{F/F}$ MEFs (Figures 1D and 1E) as well as in a *Brca2*$^{-/-}$ mouse mammary tumor-derived cell line (Evers et al., 2010; Figure S1D).

G4 Structure Stabilization Exacerbates the Telomere Fragility in *Brca2*-Deleted MEFs

Telomere fragility indicates a telomere replication defect (Martínez et al., 2009; Sfeir et al., 2009), which is thought to stem from the potential of telomere DNA sequences to adopt G4 secondary structures known to obstruct replication fork progression. To test whether telomere fragility in HR-deficient cells was linked to G4 formation, we used the G4 ligand PDS (Rodriguez et al., 2008, 2012) to treat *p53*$^{-/-}$ MEFs, which are known to proliferate in the presence of DNA damage, followed by immunofluorescence combined with telomere FISH (IF-FISH) detection. Exposure to PDS led to accumulation of nuclear foci of the Ser139-phosphorylated form of histone H2AX (γH2AX, Figure 2A), a well-established DSB marker. A subset of these foci colocalized with chromosome ends (Figure 2A, yellow arrowheads), while others localized intrachromosomally (Figure 2A, gray arrowheads). A similar PDS effect has been reported in human cells (Rodriguez et al., 2012). In addition, PDS triggered a dramatic reduction in the intensity of telomere FISH signals corresponding to the G-rich telomere strand (Figures 2A and S1E). In these images, the same exposure time was used for image

acquisition of untreated and PDS-treated cells, to enable comparison of the telomeric signal intensity between the two samples. In contrast, in Figure 2B the exposure time was increased when acquiring images of PDS-treated samples (but not in untreated controls) in order to compensate for the reduced telomeric FISH signal and to enable quantification of fragile telomeres. G4 stabilization significantly enhanced the telomere fragility characteristic of *Brca2*-deleted MEFs (Figure 2B), suggesting that persistent G4 structures contribute to the telomere replication defect intrinsic to cells lacking BRCA2.

We next monitored the viability of *Brca2*-deleted MEFs grown in the presence of PDS or poly (ADP-ribose) polymerase 1 (PARP1) inhibitor olaparib. Even though PDS was moderately toxic to BRCA2-proficient MEFs, we detected a more prominent dose-dependent reduction in the viability of Cre-treated $Brca2^{F/-}$ MEFs exposed to this compound or olaparib (Figure 2C). The same specific elimination by PDS was observed for BRCA2-deficient V-C8 hamster cells (Kraakman-van der Zwet et al., 2002; Figure S2A) and $Brca2^{-/-}$ mouse mammary tumor-derived cells (Figure S2B).

The tumor suppressor BRCA1 plays a key role in HR by promoting end resection, which enables loading of the RAD51 recombinase and initiation of HR-mediated repair. This activity of BRCA1 is antagonized by 53BP1, which protects broken DNA ends and channels their repair into non-homologous end joining (NHEJ) (Bouwman et al., 2010; Bunting et al., 2010). To address whether NHEJ deficiency also sensitizes cells to G4 stabilizing agents, similarly to HR ablation, we tested whether *Brca1* or 53BP1 loss confers sensitivity to PDS. Only viability of *Brca1*-deleted cells was affected by exposure to PDS (Figures 2D and 2E), suggesting that G4 stabilization is specifically toxic to HR-, but not to NHEJ-compromised cells. A similar HR-specific effect was observed in response to olaparib (Figures 2D and 2E).

G4-Interacting Compounds Specifically Kill HR-Deficient Human Cells

To investigate whether PDS-induced G4 stabilization affects viability of human cells lacking BRCA2, we used a matched pair of BRCA2-proficient and deficient DLD1 colorectal adenocarcinoma cell lines (Hucl et al., 2008). Exposure of BRCA2-deficient DLD1 cells to PDS led to a marked decrease in viability compared to BRCA2-proficient cells within 3 days (Figure S2C), which became more pronounced after six days of treatment (Figure 3A). The PARP1 inhibitor olaparib was used as a control in these experiments based on its ability to preferentially kill BRCA2-deficient cells (Figure 3B). Importantly, PDS toxicity to cells lacking BRCA2 was recapitulated in clonogenic assays in which cells were exposed to the drug for only 24 hr (Figure S2D).

BRCA2 plays a central role in HR repair by recruiting RAD51 to the sites of DSBs ssDNA present at stalled replication forks to initiate strand-invasion reactions. We therefore investigated whether RAD51 deficiency sensitized cells to G4-interacting compounds, similarly to loss of BRCA2. Indeed, exposure to PDS caused a substantial drop in cell viability of HEK293T cells lacking RAD51 compared to control cells (Figures 3C and S2C). Olaparib reduced the viability of RAD51-depleted cells; however,

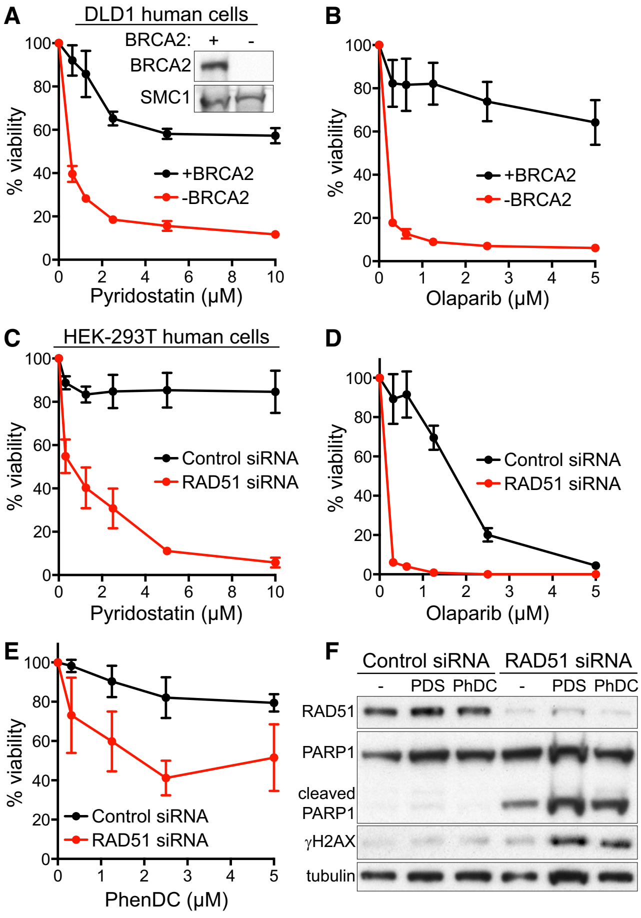

Figure 3. Effect of PDS on BRCA2- or RAD51-Deficient Human Cell Viability

(A and B) Dose-dependent viability assays of DLD1 cells, BRCA2 proficient (+BRCA2) or deficient (−BRCA2), treated with indicated concentrations of PDS (A) or olaparib (B).

(C–E) Dose-dependent viability assays of HEK293T cells transfected with control or RAD51 siRNA treated with indicated concentrations of PDS (C), olaparib (D), or PhenDC (E). Graphs shown are representative of at least two independent experiments, each performed in triplicate. Error bars represent SD of triplicate values obtained from a single experiment.

(F) Whole-cell extracts prepared after 4 days of treatment with 2 μM PDS or PhenDC (PhDC) were immunoblotted as indicated. Tubulin was used as a loading control. See also Figure S2.

it also exhibited toxicity against control cells (Figure 3D). Moreover, RAD51 depletion sensitized HEK293T cells to the G4 ligand PhenDC (Figure 3E; Piazza et al., 2010). In western blot analyses (Figure 3F), PDS and PhenDC both induced apoptosis specifically in RAD51-deficient cells, detected by cleaved PARP1 and γH2AX expression, a well-established marker for DNA damage that is also induced by apoptosis (Rogakou et al., 2000). Thus, treatment with G4-interacting agents elicits DNA damage leading to specific killing of cells lacking BRCA2 or RAD51. While PhenDC drastically reduced viability of $Brca1^{-/-}$ mouse tumor-derived cells (Figure S2E), its toxicity against BRCA2-deficient V-C8 cells was rather modest (Figure S2A).

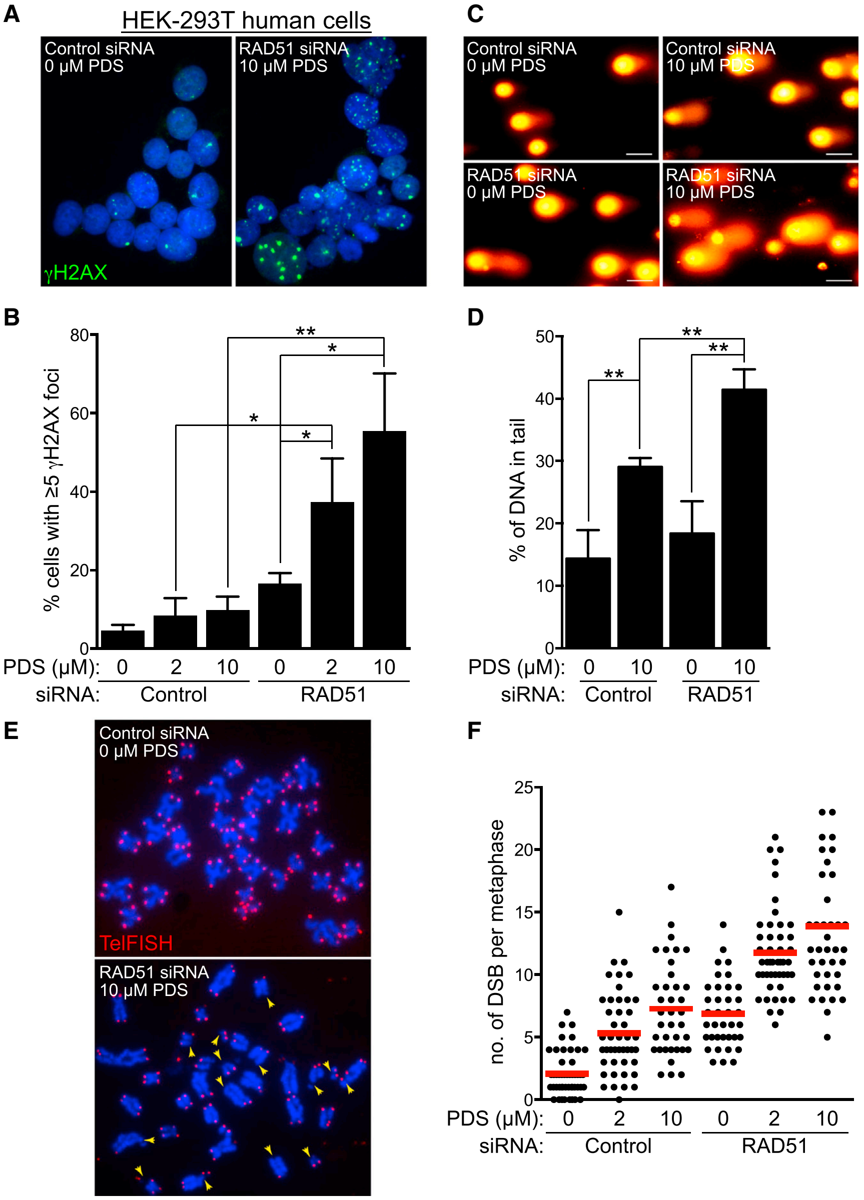

Figure 4. Elevated Levels of DNA Damage in RAD51-Deficient Human Cells Treated with PDS

(A) Representative images of HEK293T cells transfected with control or RAD51 siRNA and treated with PDS for 4 days before processing for immunofluorescence staining with anti-γH2AX antibody (green). DNA was counterstained with DAPI (blue).

(B) Quantification of the frequency of cells with ≥ 5 γH2AX foci treated as in (A); n = 3; error bars, SD. p values were calculated using an unpaired two-tailed t test (*$p \leq 0.05$; **$p \leq 0.01$).

(C) Representative images of cells treated as in (A) processed for comet assays. Scale bar, 50 μm.

(D) Quantification of tail moment using comet assays of cells treated as in (A); n = 3; error bars, SD. p values were calculated using an unpaired two-tailed t test (*$p \leq 0.05$).

(E) Representative images of FISH analysis of metaphase chromosome spreads of cells treated as in (A) with a Cy3-conjugated telomeric probe (red). DNA was counterstained with DAPI (blue). Arrowheads point to chromatid/chromosome breaks.

(F) Quantification of mean DSB frequencies (red bars) in cells treated as in (A). Approximately 40 metaphases were analyzed for each sample. See also Figure S3.

PDS Enhances DNA Damage Levels in HR-Compromised Cells

We next focused on understanding the mechanism underlying the impaired viability of RAD51-deficient cells in the presence of PDS. Quantification of γH2AX foci as detected by immunofluorescence staining (Figures 4A and S3A) revealed a significant increase in the frequency of HR-deficient cells containing γH2AX foci in response to PDS (Figure 4B). On average, 16.5% of untreated RAD51-depleted cells exhibited five or more γH2AX foci, which escalated to 37.3% and 55.4% following treatment with 2 or 10 μM PDS, respectively. In control cells, the focal γH2AX accumulation upon PDS treatment was not statistically significant (from 4.5% to 8.2% and 9.7%). Alkaline comet assays, in which the percentage of tail DNA relative to total DNA was indicative of the levels of DNA damage present in an individual cell (Figure 4C), confirmed that PDS-triggered DNA damage was significantly augmented in HR-deficient compared to HR-proficient cells (Figure 4D). In agreement with this, PDS elicited increased numbers of DBSs per metaphase in control cells, and RAD51 depletion further enhanced this effect (Figures 4E, 4F, and S3B). In these images we used telomeric FISH probes that helped define individual chromosomes. Given the reduced intensity of the FISH signal for the telomeric G-rich strand in PDS-treated samples, we increased acquisition time for these images, as described for Figure 2B. The average number of breaks detected in this assay reflects break accumulation in mitosis, while cells with higher levels of DNA damage most likely arrest during G2/M transition. Consistently, PDS treatment and RAD51 depletion caused a decrease in the mitotic index (Figure S3C). Taken together, these data supported the concept that G4 stabilization triggers DNA damage, with lethal consequences in cells with compromised capacity for HR-mediated repair.

Acute Replication Stress Induced by PDS in Cells Lacking RAD51 or BRCA2

PDS has been proposed to induce replication-dependent DNA damage (Rodriguez et al., 2012). This prompted us to monitor the assembly of replication protein A (RPA) subnuclear foci

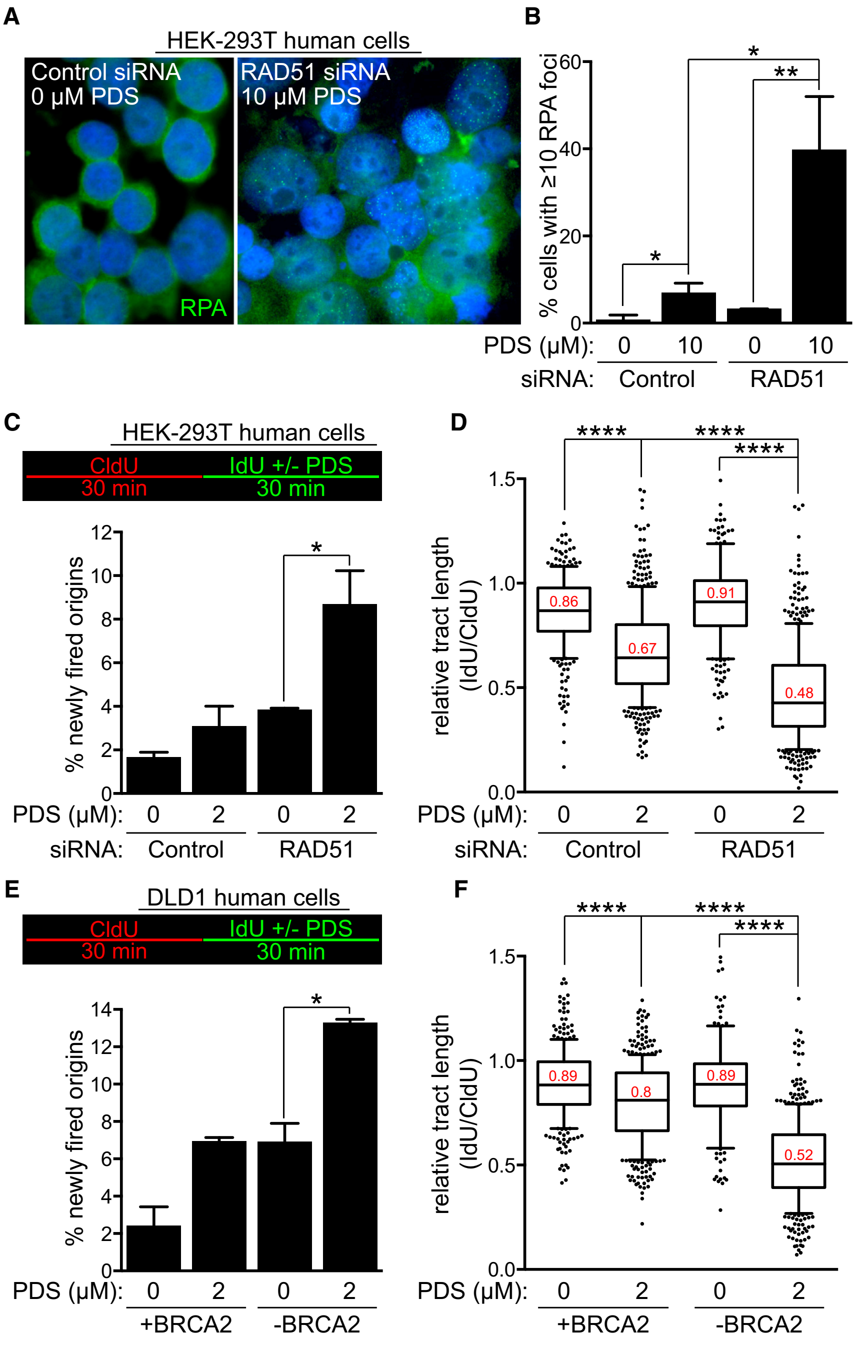

Figure 5. PDS Exacerbates the Replication Defect of RAD51- and BRCA2-Deficient Human Cells.

(A) Representative images of HEK293T cells transfected with control or RAD51 siRNA and treated with PDS for 4 days before processing for immunofluorescence staining with anti-RPA antibody (green). DNA was counterstained with DAPI (blue).

(B) Quantification of the frequency of cells with ≥10 RPA foci treated as in (A); n = 3; error bars, SD. p values were calculated using an unpaired two-tailed t test (*p ≤ 0.05; **p ≤ 0.01).

(C) HEK293T cells transfected with control or RAD51 esiRNA were processed for DNA fiber analysis as outlined in the inset, followed by quantification of the frequency of newly fired origins (n = 2; error bars, SD). p values were calculated using an unpaired two-tailed t test (*p ≤ 0.05).

(D) Quantification of the relative replication tract length (IdU/CldU) in cells treated as in (C). Middle line represents median, and the box extends from the 25th to 75th percentiles. The whiskers mark the 10th and 90th percentiles. p values were calculated using a Mann-Whitney test (n = 2; ****p < 0.0001).

(E) DLD1 cells, BRCA2 proficient (+BRCA2) or deficient (−BRCA2), were processed for DNA fiber analysis as outlined in the inset, followed by quantification of the frequency of newly fired origins (n = 2; error bars, SD). p values were calculated using an unpaired two-tailed t test (*p ≤ 0.05).

(F) Quantification of the relative replication tract length (IdU/CldU) in cells treated as in (E). Middle line represents median, and the box extends from the 25th to 75th percentiles. The whiskers mark the 10th and 90th percentiles. p values were calculated using a Mann-Whitney test (n = 2; ****p < 0.0001).

See also Figure S4.

(Figures 5A and S4A) as a readout for genome-wide ssDNA accumulation. PDS induced an approximately 6-fold increase in the levels of RPA foci in control cells and approximately 12-fold increase in RAD51-deficient cells (Figure 5B). RPA accumulation on the chromatin, together with elevated frequency of origin firing and reduced replication rates, represents signatures of replicative stress (Zeman and Cimprich, 2014). To define the nature of this replication defect, we performed DNA fiber analyses in which replication tracks were labeled with consecutive 30 min pulses of CldU and IdU. Addition of PDS during the second pulse enabled us to evaluate the immediate effect of G4 stabilization on replication. Relative tract length was decreased significantly in PDS-treated cells compared to untreated cells, an effect that was more prominent in cells lacking RAD51 or BRCA2 expression (Figures 5D, 5F, S4B, and S4C). PDS may induce persistent G4s that reduce replication rate or cause DNA breakage that obstructs replication fork progression. Possibly as a compensatory mechanism, PDS treatment significantly increased the number of newly fired origins, detected as green tract only, specifically in RAD51- (Figure 5C) or BRCA2-deficient cells (Figure 5E). Notably, elevated origin firing was also detected in untreated HR-deficient cells. Thus, the replication stress endogenous to HR-compromised cells may be potentiated by chemical G4 stabilization to levels that become lethal. To test this possibility, we used aphidicolin as an alternative means to elicit replication stress (Figure S4D). Treatment with a nontoxic

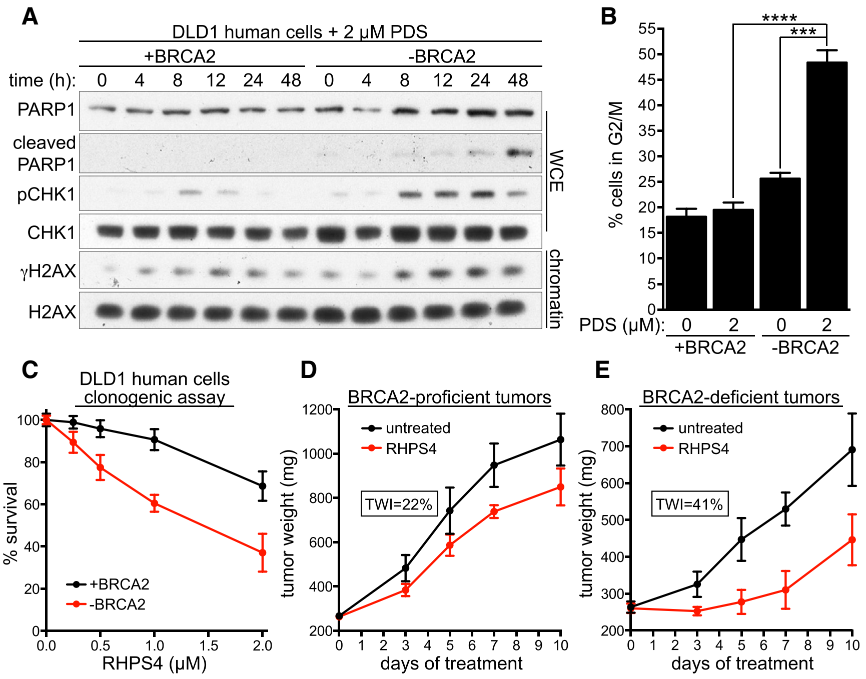

Figure 6. Effect of PDS on Viability of BRCA2-Deficient Cells and Tumors

(A) DLD1 cells, BRCA2 proficient (+BRCA2) or deficient (−BRCA2), were incubated with 2 μM PDS. Whole-cell extracts (WCE) or chromatin fractions prepared at indicated time points were immunoblotted as shown.

(B) Cells treated as in (A) were processed for FACS analyses of DNA content after 48 hr. Quantification of the percentage of cells in G2/M is shown (n = 3; error bars, SD). p values were calculated using an unpaired two-tailed t test (***p ≤ 0.001; ****p ≤ 0.0001).

(C) Clonogenic survival assays of DLD1 cells, BRCA2 proficient (+BRCA2) or deficient (−BRCA2), exposed to the indicated concentrations of RHPS4 for 24 hr. Error bars represent SD of triplicate values obtained from a single experiment.

(D and E) Mean tumor weights in untreated and RHPS4-treated mice injected with BRCA2-proficient (+BRCA2; D) or deficient (−BRCA2; E) DLD1 cells (n = 8; error bars, SD). Tumor weight inhibition (TWI) was calculated at the time point of maximum effect. See also Figures S5 and S6.

dose of aphidicolin led to sensitization of BRCA2-proficient cells to PDS. The synergy between the two compounds was not observed in BRCA2-deficient cells. This suggested that BRCA2 abrogation and aphidicolin treatment cause equivalent levels of replication stress and DNA damage, leading to comparable outcomes in the context of G4 stabilization by PDS.

PDS Triggers Checkpoint Activation and G2/M Arrest in HR-Defective Cells

Given the profound antiproliferative effect of PDS in BRCA2- or RAD51-deficient cells, we examined its impact on the DNA damage response (DDR). In cells lacking BRCA2 or RAD51 expression, continuous PDS treatment for 4 days elicited a robust phosphorylation of KAP1 (Ser824), CHK1 (Ser314/345), and RPA (Ser4/8), indicative of ATM/ATR checkpoint activation, as well as PARP1 cleavage, a marker for apoptosis (Figures S5A and S5B). To establish whether DDR preceded apoptosis onset, we monitored the response to PDS over a 48 hr interval. In BRCA2-deficient cells, PDS triggered H2AX and CHK1 phosphorylation after 8 hr of treatment, whereas PARP1 cleavage was initiated between 24 hr and 48 hr (Figure 6A). RAD51-depleted HEK293T cells similarly exhibited γH2AX activation prior to PARP1 cleavage (Figure S5C). These results indicate that PDS-induced DDRs are provoked prior to apoptosis in cells lacking BRCA2 or RAD51. Accordingly, BRCA2- and RAD51-deficient cells accumulated in G2/M after PDS treatment (Figures 6B and S6A). A decrease in S-phase cells further reflected the effect of PDS on cell-cycle progression and checkpoint activation specifically in HR-deficient cells (Figures S6A and S6B). PDS induces replication-associated DSBs, although transcription-related DNA damage may accumulate in stages of the cell cycle other than S phase (Rodriguez et al., 2012). To address whether PDS causes damage in noncycling cells, G0/G1 arrest was induced by serum starvation in the presence or absence of PDS. Arrested cells lacked the ability to incorporate the thymidine analog EdU, in contrast to cells released into the cell cycle by serum addition to the media (Figure S6C). Quantification of γH2AX-positive cells demonstrated that PDS treatment for 48 hr did not induce DNA damage in noncycling cells, while a marked increase in the percentage of cells expressing γH2AX was detected in the subset of cycling cells treated with PDS (Figure S6C).

In Vivo Responses of BRCA2-Deficient Tumors to G4 Ligands

Regardless of the effective suppression of HR-deficient cell viability and survival by PDS-mediated G4 stabilization (Figures 3A and S2D), the efficacy of PDS could not be established in vivo due to its toxicity predicted by in vitro studies (Rodriguez et al., 2012). Instead, we tested in our cellular models a previously reported G4-stabilizing drug, RHPS4 (Gavathiotis et al., 2003; Gowan et al., 2001; Heald et al., 2002), with well-characterized pharmacological features (Leonetti et al., 2008; Salvati et al., 2007). RHPS4 markedly diminished survival of BRCA2-deficient DLD1 cells relative to BRCA2-proficient cells (Figure 6C). To test its efficacy in vivo, DLD1 cells were injected into mice and allowed to form palpable tumors. In line with previous publications reporting the antitumor effect of RHPS4 (Leonetti et al., 2008; Salvati et al., 2007), this drug repressed growth of BRCA2-proficient tumors as assessed by tumor weight inhibition (TWI) (22%, Figure 6D). Importantly, the growth inhibitory effect of RHPS4 was almost twice as pronounced in BRCA2-deficient tumors (TWI = 41%, Figure 6E). RHPS4 treatment elicited a marked delay in tumor regrowth (approximately 7 days in BRCA2-deficient compared to 4 days in BRCA2-proficient tumors). Thus, our conclusions based on cellular models can be translated

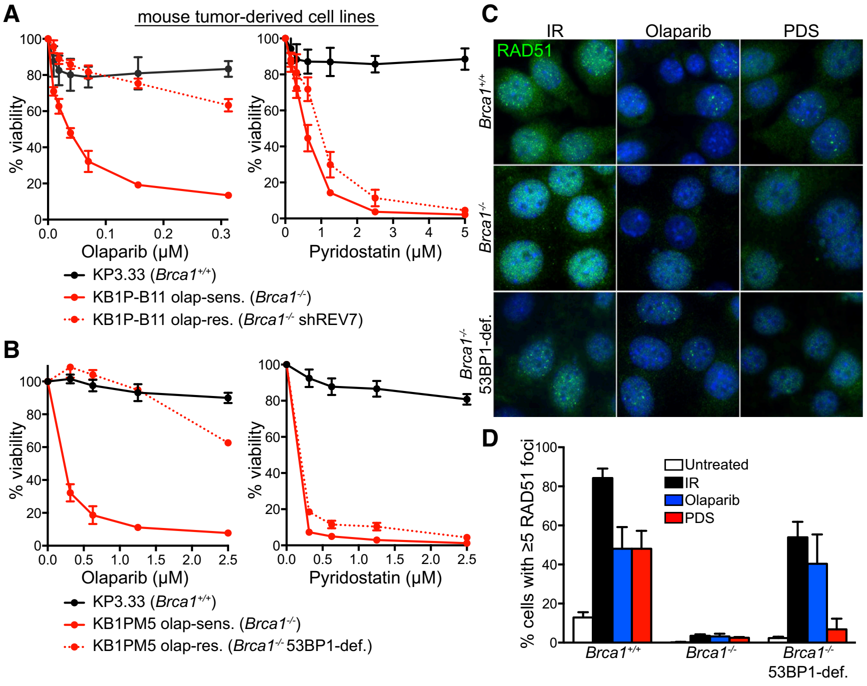

Figure 7. Olaparib-Resistant *Brca1*-Deleted Tumor Cells Exhibit PDS Sensitivity

(A and B) Dose-dependent viability assays of mouse mammary tumor-derived cell lines deficient in REV7 (A) or 53BP1 (B) treated with indicated concentrations of PDS or olaparib. Graphs shown are representative of at least two independent experiments, each performed in triplicate. Error bars represent SD of triplicate values obtained from a single experiment.

(C) Representative images of cells described in (A) incubated with 0.5 μM olaparib (OLAP), PDS for 40 hr, or irradiated with 10 Gy of IR followed by 1 hr recovery and processed for immunofluorescence staining with anti-RAD51 antibody (green). DNA was counterstained with DAPI (blue).

(D) Quantification of the frequency of cells with ≥5 RAD51 foci in cells treated as in (C); n = 2; error bars, SD; >200 nuclei were analyzed for each condition per replica. See also Figure S7.

in vivo and support the concept that G4-stabilizing compounds identify a class of drugs, which may facilitate future development of novel therapeutic strategies for targeting BRCA2-deficient tumors.

PDS Kills Olaparib-Resistant Tumor-Derived Cells

Treatment of BRCA-deficient tumors poses a major challenge in the clinic due to the rapid emergence of drug resistance. To test the potential of PDS to eliminate *Brca1*-deficient mouse tumor-derived cells refractory to olaparib, we used two *Brca1*$^{-/-}$ cellular mouse models, in which olaparib resistance was mediated by concomitant loss of REV7 (Figure 7A; Xu et al., 2015) or 53BP1 (Figure 7B; Jaspers et al., 2013). Cells carrying intact *Brca1* (*Brca1*$^{+/+}$) showed no sensitivity to PDS or olaparib, while cells established from a *Brca1*$^{-/-}$ tumor were sensitive to both drugs, as determined in viability and clonogenic assays (Figures 7A, 7B, S7A, and S7B). Strikingly, olaparib-resistant *Brca1*-deficient cells lacking REV7 or 53BP1 expression (*Brca1*$^{-/-}$ shREV7; *Brca1*$^{-/-}$ 53BP1-deficient) were hypersensitive to PDS (Figures 7A, 7B, S7A, and S7B). These effects were recapitulated in human cells, in which 53BP1 and BRCA1 were depleted using siRNA (Figure S7C). Our results, therefore, strongly suggest that BRCA1-deficient cells, including those resistant to PARP inhibitors, can be targeted by treatment with G4-stabilizing compounds.

HR restoration in *Brca1*-deleted cells and tumors is driven by 53BP1 loss, which enables survival (Bouwman et al., 2010; Bunting et al., 2010). Moreover, ionizing radiation (IR)-induced RAD51 foci assemble in olaparib-resistant *Brca1*$^{-/-}$, 53BP1-deficient cells (albeit not at the same level as in *Brca1*$^{+/+}$ cells), but not in olaparib-sensitive *Brca1*$^{-/-}$ tumor-derived cells (Jaspers et al., 2013). Our data (Figures 7C and 7D) demonstrate that olaparib treatment itself triggers RAD51 foci in wild-type and olaparib-resistant, but not olaparib-sensitive, cells, thereby providing a direct correlation between olaparib-induced HR reactivation and its impact on cell survival. PDS treatment induced RAD51 foci in *Brca1*$^{+/+}$ cells, similarly to olaparib (Figures 7C and 7D). However, RAD51 foci were absent in both olaparib-sensitive and olaparib-resistant cells upon treatment with PDS (Figures 7C and 7D), suggesting that failure to reactivate HR repair contributes to the toxicity of this compound in *Brca1*$^{-/-}$, 53BP1-deficient cells. To gain further insight into the mechanism of RAD51 foci suppression, we evaluated the levels of chromatin-associated RPA, indicative of end resection activity. In the chromatin fraction of PDS-treated cells, less RPA was detected than in cells exposed to olaparib or IR (Figure S7D). Thus, impaired HR reactivation upon PDS treatment in a *Brca1*$^{-/-}$, 53BP1-deficient background is likely caused by defects in end resection.

DISCUSSION

The ability of G-rich DNA to adopt G4 secondary structures in vitro was reported over 50 years ago (Gellert et al., 1962). Although G4s are thought to positively regulate key cellular processes, they can also obstruct replication-fork progression, leading to genomic instability (Tarsounas and Tijsterman, 2013). In this study, we establish that effective replication of G4 structures requires HR activities. G4s represent potent replication barriers, and HR provides a well-characterized mechanism for replication-fork restart and repair of replication-associated DSBs. Yet, the potential requirement for HR in G4 stability has not been investigated, with the notable exception of *Saccharomyces cerevisiae pif1* mutants, in which attempts to restart forks stalled in the vicinity of G4 structures generated recombination intermediates. This suggested a role for HR in fork restart when Pif1 activity is abrogated (Ribeyre et al., 2009).

HR Is Required for Effective Replication of Genomic Regions with G4-Forming Potential

HR factors have previously been implicated in telomere maintenance (Tacconi and Tarsounas, 2015). In the present work, we used a plasmid-based replication assay in human cells to show that replication of telomeric repeats is ineffective when key HR activities are abrogated. Two lines of evidence established the HR requirement for replication of the G-rich telomeric strand. First, telomere fragility triggered by HR gene deletion was specific to the G-rich telomeric strand, which possesses G4-forming potential. Second, disruption of the G4-forming telomeric repeats through G-to-C substitutions rescued its replication defect in HR-deficient cells.

We propose that HR promotes replication in the presence of obstructive G4 structures by restarting stalled forks and/or by repairing replication-associated DSBs within telomeres, rather than contributing to telomeric G4 dissolution per se. The latter process is likely mediated by the shelterin component TRF1, which recruits BLM helicase to telomeres to unwind G4 structures (Zimmermann et al., 2014). The concept that HR and shelterin provide distinct mechanisms for telomere replication is supported by the synthetic lethality observed between *Brca2* and *Trf1* gene deletions in immortalized MEFs, accompanied by additive levels of telomere fragility (Badie et al., 2010). Inhibition of BLM expression with shRNA in *Brca2*-deleted cells similarly induced cell-cycle arrest (J.Z. and M.T., unpublished data), further arguing that independent mechanisms act during telomere replication to dismantle G4s and to repair the DNA damage induced by persistent G4 structures.

Importantly, G4 stabilization by PDS reduced viability of mouse, human, and hamster cells lacking BRCA1, BRCA2, or RAD51. It exacerbated telomere fragility and DNA damage levels in HR-deficient cells. Conceivably, unresolved G4s presenting intrachromosomally or within telomeres are converted to DSBs, eliciting in turn checkpoint activation, cell-cycle arrest, and/or specific elimination of HR-compromised cells by apoptotic mechanisms.

The efficacy of PDS in cell killing was previously attributed to its genome-wide toxicity, suggested by the accumulation of DNA damage marker γH2AX at genomic sites with computationally inferred G4-forming sequences (Rodriguez et al., 2012). It is conceivable that the same sites may be prone to breakage in HR-deficient cells treated with PDS. Our mitotic DSB quantification illustrates the additive effect of PDS on the levels of DNA damage triggered by HR abrogation itself. A conundrum posed by this quantification was that PDS induced approximately fifteen DSBs per metaphase in cells lacking RAD51, yet in silico predictions suggested that more than 300,000 genomic sites can adopt G4 configurations (Huppert and Balasubramanian, 2005). This discrepancy could be explained by the multitude of mechanisms known to maintain genome integrity by dismantling G4s formed during genome replication (Tarsounas and Tijsterman, 2013). While most genomic G4s are dissolved by alternative mechanisms, our data suggest that a subset triggers fork stalling and DSBs, which are particularly toxic in HR-deficient cells lacking a key pathway of fork restart and break repair. G4-induced DNA damage may be repaired by error-prone mechanisms in the absence of HR, which seems insufficient for the survival of these cells. Moreover, checkpoint activation prevented entry of cells with elevated DSB levels into mitosis, which further justifies the lower number of mitotic DSBs detected in our assay.

Implications for Cancer Therapies

The work presented here demonstrates that the G4-stabilizing drug RHPS4 limits the growth of BRCA2-deficient tumors grafted in mice. The well-characterized ability of RHPS4 to trigger telomere dysfunction may contribute to its toxicity to BRCA2-deficient cells (Salvati et al., 2007). Therefore, we propose that the anticancer potential of the G4-stabilizing drug RHPS4 can be exploited in the clinic for specific targeting of BRCA2-deficient tumors. This tumor subset is likely to benefit most from this novel class of anticancer drugs. Furthermore, these results open a favorable prospective for future clinical development of PDS into a drug-like compound, with a more robust anticipated antitumor activity than RHPS4 in models for BRCA2 inactivation.

Mutations in HR genes such as *BRCA1*, *BRCA2*, or *RAD51C* predispose individuals to breast and ovarian cancers. Tumors carrying HR gene deletions are vulnerable to drugs that either introduce replication-associated DNA damage (e.g., platinum drugs) or inhibit DNA repair pathways other than HR (e.g., PARP1 inhibitors, such as olaparib). In both cases, excessive DNA-damage accumulation triggers cell death. Here, we propose that G4-binding compounds identify a novel class of molecules that can be used to target BRCA deficiency. They act by stabilizing secondary structures in genomic regions with high G-rich content, thus reducing replication fork speed and inducing RPA foci indicative of ssDNA accumulation. *BRCA* gene abrogation is associated with the same responses (Carlos et al., 2013). In the absence of HR, G4-interacting compounds are likely to elevate the endogenous replication stress to levels that become lethal due to excessive DNA-damage accumulation.

One well-documented caveat of targeted drug treatments, such as olaparib, is that tumors rapidly acquire resistance through mechanisms that include activation of P-glycoprotein drug efflux transporter, genetic *Brca1/2* re-activation, and loss of 53BP1/REV7 (Bouwman and Jonkers, 2014; Jaspers et al., 2013; Xu et al., 2015). In this work, we establish that G4-stabilizing compounds are profoundly toxic to BRCA-defective cells, including those resistant to PARP inhibitors. In particular, the striking cytotoxicity of PDS is due to the combined replication failure induced by this drug and the DNA repair defect associated with HR abrogation. Therefore, pharmacological G4 stabilization could be exploited in future therapeutic modalities targeting this difficult to treat tumor subset. Olaparib-resistant cells fail to reactivate HR in response to PDS, which may account for the lethality induced by this G4-stabilizing compound. We therefore anticipate that further clinical development of G4-stabilizing compounds will enhance their ability to selectively eliminate HR-compromised tumors, including those that have acquired resistance to existing therapies.

EXPERIMENTAL PROCEDURES

For detailed descriptions of these and additional procedures, see Supplemental Experimental Procedures.

Cell Lines, Culture Conditions, and In Vivo Experiments

HEK293T, H1299, and DLD1 cells were cultured under conventional growth conditions. In vivo experiments were performed as previously described (Salvati et al., 2007). All animal procedures were in compliance with the national and international directives (D.L. March 4, 2014, no. 26; directive 2010/63/EU of the European Parliament and of the council; Guide for the Care and Use of Laboratory Animals, United States National Research Council, 2011).

Plasmid-Based Replication Assay

Plasmid-based replication assays were performed as previously described (Sarkies et al., 2010; Szüts et al., 2008) with modifications listed in Supplemental Experimental Procedures.

RNAi

DLD1 and HEK293T cells were transfected with 40 nM siRNA using Dharmafect 1 (Dharmacon) according to manufacturer's instructions.

Cell Viability Assays

Cell viability was determined by incubation with 10 μg/ml of resazurin for 2 hr. Fluorescence was measured at 590 nm using a plate reader (POLARstar, Omega one). Cell viability was expressed relative to untreated cells of the same cell line, thus accounting for any differences in viability caused by HR deficiency. Graphs shown are representative of at least two independent experiments, each performed in triplicate. Error bars represent SD of triplicate values obtained from a single experiment.

FACS Analysis

Cells were harvested by trypsinization, washed in cold PBS, and fixed in ice-cold 70% ethanol overnight at 4°C. Following two washes in PBS, cells were incubated with 20 μg/ml propidium iodide and 10 μg/ml RNase A (Sigma) in PBS. At least 10,000 cells were analyzed by flow cytometry (Becton Dickinson). Data were processed using CellQuest (Becton Dickinson) and ModFit LT software.

Alkaline Single-Cell Gel Electrophoresis Comet Assay

The comet assay was performed as previously described (Singh et al., 1988). Tail measurement was performed using the Komet 5.5 image analysis software.

Immunofluorescence

Cells were subjected to immunofluorescence staining as described (Tarsounas et al., 2004).

Preparation of Metaphase Spreads and Telomere FISH

Metaphase spread preparation and telomeric FISH were performed as previously described (Badie et al., 2015).

Chromosome Orientation FISH and IF-FISH

For CO-FISH, cells were plated at 50%–60% confluency and treated with 10 μM bromodeoxyuridine (BrdU) for 20 hr. Colcemid (0.2 μg/ml) was added to the cells 4–6 hr before metaphases were processed for CO-FISH as previously described (Bailey et al., 2001).

For IF-FISH, metaphases were spun onto coverslips using a cytospin apparatus (Cytospin 4, Fisher) and subjected to immunofluorescence staining as described (Tarsounas et al., 2004). Samples were fixed again in 4% paraformaldehyde in PBS, and FISH was performed as described (Tarsounas et al., 2004) using 15 μg/ml Cy3-conjugated $(CCCTAA)_6$-PNA telomeric probe (Applied Biosystems).

DNA Fiber Assay

DNA fiber assays were performed as described previously (Jackson and Pombo, 1998).

Immunoblotting

SDS-PAGE and immunoblotting were performed as previously described (Badie et al., 2015). See Supplemental Experimental Procedures for antibodies used in this study.

SUPPLEMENTAL INFORMATION

Supplemental Information includes Supplemental Experimental Procedures and seven figures and can be found with this article online at http://dx.doi.org/10.1016/j.molcel.2015.12.004.

ACKNOWLEDGMENTS

E.M.C.T. and C.F. contributed equally to this work. We are grateful to Peter Bouwman and Alexandra Duarte (NKI, Amsterdam), Sven Rottenberg (Vetsuisse, University of Bern), Marie-Paule Teulade-Fichou (Curie Institute, Paris), Steve West (CRUK Clare Hall Laboratories), Hyunsook Lee (Seoul National University), and Carmen D'Angelo (Regina Elena Cancer Institute, Italy) for valuable reagents and/or technical suggestions. Research in the S.P.J. lab is funded by Cancer Research UK program grant C6/A11224, the European Research Council, and the European Community Seventh Framework Program grant agreement number HEALTH-F2-2010-259893 (DDResponse). Core infrastructure funding was provided by Cancer Research UK grant C6946/A14492 and Wellcome Trust grant WT092096. S.P.J. receives salary from the University of Cambridge, supplemented by Cancer Research UK. Research in the K.R. lab is supported by Medical Research Council (MC_PC_12001/1), University of Oxford, and Swiss National Science Foundation (31003A_141197). Work in the A.B. lab is supported by Italian Association for Cancer Research (AIRC # #11567). Work in the J.E.S. lab is supported by a central grant to the Laboratory of Molecular Biology, Cambridge from the Medical Research Council (U105178808). J.Z. is supported by a Cancer Research UK D.Phil. Studentship and E.M.C.T. by a Medical Research Council D.Phil. Studentship. Work in the M.T. lab is supported by Cancer Research UK, Medical Research Council, University of Oxford, and EMBO Young Investigator Program.

Received: March 27, 2015
Revised: September 17, 2015
Accepted: November 2, 2015
Published: December 31, 2015

REFERENCES

Aze, A., Zhou, J.C., Costa, A., and Costanzo, V. (2013). DNA replication and homologous recombination factors: acting together to maintain genome stability. Chromosoma *122*, 401–413.

Badie, S., Escandell, J.M., Bouwman, P., Carlos, A.R., Thanasoula, M., Gallardo, M.M., Suram, A., Jaco, I., Benitez, J., Herbig, U., et al. (2010). BRCA2 acts as a RAD51 loader to facilitate telomere replication and capping. Nat. Struct. Mol. Biol. *17*, 1461–1469.

Badie, S., Carlos, A.R., Folio, C., Okamoto, K., Bouwman, P., Jonkers, J., and Tarsounas, M. (2015). BRCA1 and CtIP promote alternative non-homologous end-joining at uncapped telomeres. EMBO J. *34*, 828.

Bailey, S.M., Cornforth, M.N., Kurimasa, A., Chen, D.J., and Goodwin, E.H. (2001). Strand-specific postreplicative processing of mammalian telomeres. Science *293*, 2462–2465.

Biffi, G., Tannahill, D., McCafferty, J., and Balasubramanian, S. (2013). Quantitative visualization of DNA G-quadruplex structures in human cells. Nat. Chem. *5*, 182–186.

Bouwman, P., and Jonkers, J. (2014). Molecular pathways: how can BRCA-mutated tumors become resistant to PARP inhibitors? Clin. Cancer Res. *20*, 540–547.

Bouwman, P., Aly, A., Escandell, J.M., Pieterse, M., Bartkova, J., van der Gulden, H., Hiddingh, S., Thanasoula, M., Kulkarni, A., Yang, Q., et al. (2010). 53BP1 loss rescues BRCA1 deficiency and is associated with triple-negative and BRCA-mutated breast cancers. Nat. Struct. Mol. Biol. *17*, 688–695.

Bunting, S.F., Callén, E., Wong, N., Chen, H.T., Polato, F., Gunn, A., Bothmer, A., Feldhahn, N., Fernandez-Capetillo, O., Cao, L., et al. (2010). 53BP1 inhibits

homologous recombination in Brca1-deficient cells by blocking resection of DNA breaks. Cell *141*, 243–254.

Carlos, A.R., Escandell, J.M., Kotsantis, P., Suwaki, N., Bouwman, P., Badie, S., Folio, C., Benitez, J., Gomez-Lopez, G., Pisano, D.G., et al. (2013). ARF triggers senescence in Brca2-deficient cells by altering the spectrum of p53 transcriptional targets. Nat. Commun. *4*, 2697.

Chambers, V.S., Marsico, G., Boutell, J.M., Di Antonio, M., Smith, G.P., and Balasubramanian, S. (2015). High-throughput sequencing of DNA G-quadruplex structures in the human genome. Nat. Biotechnol. *33*, 877–881.

Evers, B., Schut, E., van der Burg, E., Braumuller, T.M., Egan, D.A., Holstege, H., Edser, P., Adams, D.J., Wade-Martins, R., Bouwman, P., and Jonkers, J. (2010). A high-throughput pharmaceutical screen identifies compounds with specific toxicity against BRCA2-deficient tumors. Clin. Cancer Res. *16*, 99–108.

Gavathiotis, E., Heald, R.A., Stevens, M.F., and Searle, M.S. (2003). Drug recognition and stabilisation of the parallel-stranded DNA quadruplex d(TTAGGGT)4 containing the human telomeric repeat. J. Mol. Biol. *334*, 25–36.

Gellert, M., Lipsett, M.N., and Davies, D.R. (1962). Helix formation by guanylic acid. Proc. Natl. Acad. Sci. USA *48*, 2013–2018.

Gomez, D., Wenner, T., Brassart, B., Douarre, C., O'Donohue, M.F., El Khoury, V., Shin-Ya, K., Morjani, H., Trentesaux, C., and Riou, J.F. (2006). Telomestatin-induced telomere uncapping is modulated by POT1 through G-overhang extension in HT1080 human tumor cells. J. Biol. Chem. *281*, 38721–38729.

Gowan, S.M., Heald, R., Stevens, M.F., and Kelland, L.R. (2001). Potent inhibition of telomerase by small-molecule pentacyclic acridines capable of interacting with G-quadruplexes. Mol. Pharmacol. *60*, 981–988.

Halazonetis, T.D., Gorgoulis, V.G., and Bartek, J. (2008). An oncogene-induced DNA damage model for cancer development. Science *319*, 1352–1355.

Hashimoto, Y., Ray Chaudhuri, A., Lopes, M., and Costanzo, V. (2010). Rad51 protects nascent DNA from Mre11-dependent degradation and promotes continuous DNA synthesis. Nat. Struct. Mol. Biol. *17*, 1305–1311.

Heald, R.A., Modi, C., Cookson, J.C., Hutchinson, I., Laughton, C.A., Gowan, S.M., Kelland, L.R., and Stevens, M.F. (2002). Antitumor polycyclic acridines. 8.(1) Synthesis and telomerase-inhibitory activity of methylated pentacyclic acridinium salts. J. Med. Chem. *45*, 590–597.

Henderson, A., Wu, Y., Huang, Y.C., Chavez, E.A., Platt, J., Johnson, F.B., Brosh, R.M.J., Jr., Sen, D., and Lansdorp, P.M. (2014). Detection of G-quadruplex DNA in mammalian cells. Nucleic Acids Res. *42*, 860–869.

Hucl, T., Rago, C., Gallmeier, E., Brody, J.R., Gorospe, M., and Kern, S.E. (2008). A syngeneic variance library for functional annotation of human variation: application to BRCA2. Cancer Res. *68*, 5023–5030.

Huppert, J.L., and Balasubramanian, S. (2005). Prevalence of quadruplexes in the human genome. Nucleic Acids Res. *33*, 2908–2916.

Jackson, D.A., and Pombo, A. (1998). Replicon clusters are stable units of chromosome structure: evidence that nuclear organization contributes to the efficient activation and propagation of S phase in human cells. J. Cell Biol. *140*, 1285–1295.

Jaspers, J.E., Kersbergen, A., Boon, U., Sol, W., van Deemter, L., Zander, S.A., Drost, R., Wientjens, E., Ji, J., Aly, A., et al. (2013). Loss of 53BP1 causes PARP inhibitor resistance in Brca1-mutated mouse mammary tumors. Cancer Discov. *3*, 68–81.

Kraakman-van der Zwet, M., Overkamp, W.J.I., van Lange, R.E.E., Essers, J., van Duijn-Goedhart, A., Wiggers, I., Swaminathan, S., van Buul, P.P.W., Errami, A., Tan, R.T.L., et al. (2002). Brca2 (XRCC11) deficiency results in radioresistant DNA synthesis and a higher frequency of spontaneous deletions. Mol. Cell. Biol. *22*, 669–679.

Lam, E.Y., Beraldi, D., Tannahill, D., and Balasubramanian, S. (2013). G-quadruplex structures are stable and detectable in human genomic DNA. Nat. Commun. *4*, 1796.

Lambert, S., Mizuno, K., Blaisonneau, J., Martineau, S., Chanet, R., Fréon, K., Murray, J.M., Carr, A.M., and Baldacci, G. (2010). Homologous recombination restarts blocked replication forks at the expense of genome rearrangements by template exchange. Mol. Cell *39*, 346–359.

Leonetti, C., Scarsella, M., Riggio, G., Rizzo, A., Salvati, E., D'Incalci, M., Staszewsky, L., Frapolli, R., Stevens, M.F., Stoppacciaro, A., et al. (2008). G-quadruplex ligand RHPS4 potentiates the antitumor activity of camptothecins in preclinical models of solid tumors. Clin. Cancer Res. *14*, 7284–7291.

Lipps, H.J., and Rhodes, D. (2009). G-quadruplex structures: in vivo evidence and function. Trends Cell Biol. *19*, 414–422.

Martínez, P., Thanasoula, M., Muñoz, P., Liao, C., Tejera, A., McNees, C., Flores, J.M., Fernández-Capetillo, O., Tarsounas, M., and Blasco, M.A. (2009). Increased telomere fragility and fusions resulting from TRF1 deficiency lead to degenerative pathologies and increased cancer in mice. Genes Dev. *23*, 2060–2075.

Müller, S., Kumari, S., Rodriguez, R., and Balasubramanian, S. (2010). Small-molecule-mediated G-quadruplex isolation from human cells. Nat. Chem. *2*, 1095–1098.

Murat, P., and Balasubramanian, S. (2014). Existence and consequences of G-quadruplex structures in DNA. Curr. Opin. Genet. Dev. *25*, 22–29.

Negrini, S., Gorgoulis, V.G., and Halazonetis, T.D. (2010). Genomic instability–an evolving hallmark of cancer. Nat. Rev. Mol. Cell Biol. *11*, 220–228.

Parkinson, G.N., Lee, M.P., and Neidle, S. (2002). Crystal structure of parallel quadruplexes from human telomeric DNA. Nature *417*, 876–880.

Piazza, A., Boulé, J.B., Lopes, J., Mingo, K., Largy, E., Teulade-Fichou, M.P., and Nicolas, A. (2010). Genetic instability triggered by G-quadruplex interacting Phen-DC compounds in Saccharomyces cerevisiae. Nucleic Acids Res. *38*, 4337–4348.

Ribeyre, C., Lopes, J., Boulé, J.B., Piazza, A., Guédin, A., Zakian, V.A., Mergny, J.L., and Nicolas, A. (2009). The yeast Pif1 helicase prevents genomic instability caused by G-quadruplex-forming CEB1 sequences in vivo. PLoS Genet. *5*, e1000475.

Rodriguez, R., Müller, S., Yeoman, J.A., Trentesaux, C., Riou, J.F., and Balasubramanian, S. (2008). A novel small molecule that alters shelterin integrity and triggers a DNA-damage response at telomeres. J. Am. Chem. Soc. *130*, 15758–15759.

Rodriguez, R., Miller, K.M., Forment, J.V., Bradshaw, C.R., Nikan, M., Britton, S., Oelschlaegel, T., Xhemalce, B., Balasubramanian, S., and Jackson, S.P. (2012). Small-molecule-induced DNA damage identifies alternative DNA structures in human genes. Nat. Chem. Biol. *8*, 301–310.

Rogakou, E.P., Nieves-Neira, W., Boon, C., Pommier, Y., and Bonner, W.M. (2000). Initiation of DNA fragmentation during apoptosis induces phosphorylation of H2AX histone at serine 139. J. Biol. Chem. *275*, 9390–9395.

Salvati, E., Leonetti, C., Rizzo, A., Scarsella, M., Mottolese, M., Galati, R., Sperduti, I., Stevens, M.F., D'Incalci, M., Blasco, M., et al. (2007). Telomere damage induced by the G-quadruplex ligand RHPS4 has an antitumor effect. J. Clin. Invest. *117*, 3236–3247.

Sarkies, P., Reams, C., Simpson, L.J., and Sale, J.E. (2010). Epigenetic instability due to defective replication of structured DNA. Mol. Cell *40*, 703–713.

Schaffitzel, C., Berger, I., Postberg, J., Hanes, J., Lipps, H.J., and Plückthun, A. (2001). In vitro generated antibodies specific for telomeric guanine-quadruplex DNA react with Stylonychia lemnae macronuclei. Proc. Natl. Acad. Sci. USA *98*, 8572–8577.

Schlacher, K., Christ, N., Siaud, N., Egashira, A., Wu, H., and Jasin, M. (2011). Double-strand break repair-independent role for BRCA2 in blocking stalled replication fork degradation by MRE11. Cell *145*, 529–542.

Sfeir, A., Kosiyatrakul, S.T., Hockemeyer, D., MacRae, S.L., Karlseder, J., Schildkraut, C.L., and de Lange, T. (2009). Mammalian telomeres resemble fragile sites and require TRF1 for efficient replication. Cell *138*, 90–103.

Singh, N.P., McCoy, M.T., Tice, R.R., and Schneider, E.L. (1988). A simple technique for quantitation of low levels of DNA damage in individual cells. Exp. Cell Res. *175*, 184–191.

Suwaki, N., Klare, K., and Tarsounas, M. (2011). RAD51 paralogs: roles in DNA damage signalling, recombinational repair and tumorigenesis. Semin. Cell Dev. Biol. *22*, 898–905.

Szüts, D., Marcus, A.P., Himoto, M., Iwai, S., and Sale, J.E. (2008). REV1 restrains DNA polymerase zeta to ensure frame fidelity during translesion synthesis of UV photoproducts in vivo. Nucleic Acids Res. *36*, 6767–6780.

Tacconi, E.M., and Tarsounas, M. (2015). How homologous recombination maintains telomere integrity. Chromosoma *124*, 119–130.

Tahara, H., Shin-Ya, K., Seimiya, H., Yamada, H., Tsuruo, T., and Ide, T. (2006). G-Quadruplex stabilization by telomestatin induces TRF2 protein dissociation from telomeres and anaphase bridge formation accompanied by loss of the 3′ telomeric overhang in cancer cells. Oncogene *25*, 1955–1966.

Tarsounas, M., and Tijsterman, M. (2013). Genomes and G-quadruplexes: for better or for worse. J. Mol. Biol. *425*, 4782–4789.

Tarsounas, M., Muñoz, P., Claas, A., Smiraldo, P.G., Pittman, D.L., Blasco, M.A., and West, S.C. (2004). Telomere maintenance requires the RAD51D recombination/repair protein. Cell *117*, 337–347.

Xu, G., Chapman, J.R., Brandsma, I., Yuan, J., Mistrik, M., Bouwman, P., Bartkova, J., Gogola, E., Warmerdam, D., Barazas, M., et al. (2015). REV7 counteracts DNA double-strand break resection and affects PARP inhibition. Nature *521*, 541–544.

Zeman, M.K., and Cimprich, K.A. (2014). Causes and consequences of replication stress. Nat. Cell Biol. *16*, 2–9.

Zimmermann, M., Kibe, T., Kabir, S., and de Lange, T. (2014). TRF1 negotiates TTAGGG repeat-associated replication problems by recruiting the BLM helicase and the TPP1/POT1 repressor of ATR signaling. Genes Dev. *28*, 2477–2491.

Molecular Cell

Supplemental Information

Targeting BRCA1 and BRCA2 Deficiencies with G-Quadruplex-Interacting Compounds

Jutta Zimmer, Eliana M. C. Tacconi, Cecilia Folio, Sophie Badie, Manuela Porru, Kerstin Klare, Manuela Tumiati, Enni Markkanen, Swagata Halder, Anderson Ryan, Stephen P. Jackson, Kristijan Ramadan, Sergey G. Kuznetsov, Annamaria Biroccio, Julian E. Sale, and Madalena Tarsounas

Figure S1 (related to Figures 1 and 2)

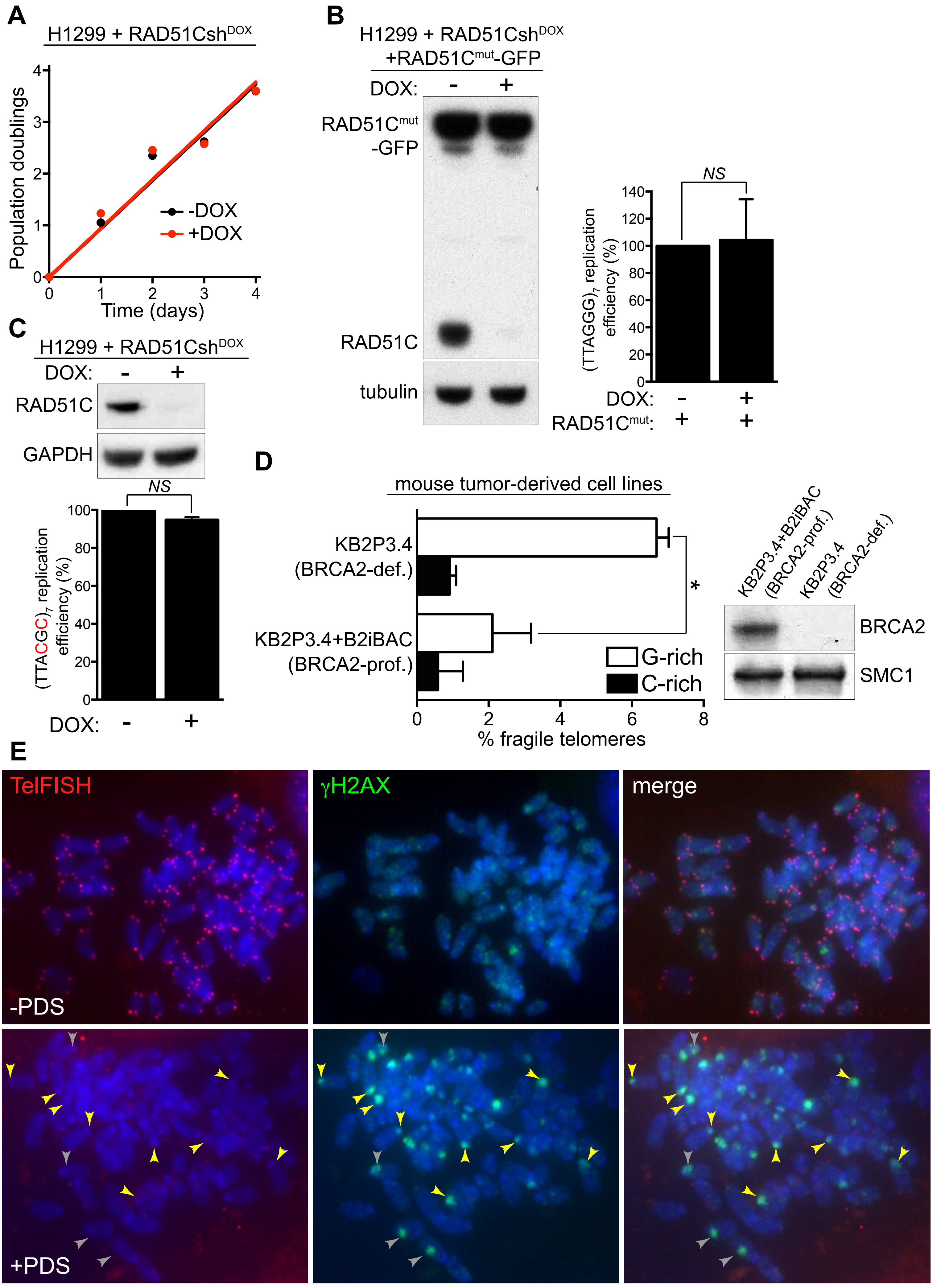

Figure S1 (related to Figures 1 and 2). (A) Proliferation curves of human H1299 cells expressing a doxycycline (DOX)-inducible RAD51C shRNA grown in the presence or absence of DOX. (B) Target specificity of the RAD51C shRNA and replication efficiency of mutated telomere sequences. H1299 cells expressing a DOX-inducible RAD51C shRNA were transfected with an shRNA-resistant RAD51C-GFP expression vector. Cell extracts prepared from cells grown in the presence or absence of DOX were immunoblotted as indicated. Tubulin was used as a loading control. Replication efficiency of a plasmid containing $(TTAGGG)_7$ was determined relative to the empty vector (*n*=2; error bars, SD). *P* values were calculated using an unpaired two-tailed *t*-test. NS, $P > 0.05$. (C) Cell extracts prepared from human H1299 cells expressing a DOX-inducible RAD51C shRNA grown in the presence or absence of DOX were immunoblotted as indicated. GAPDH was used as a loading control. Replication efficiency of a plasmid containing the $(TTACGC)_7$ array, in which G to C substitutions abolished the G4-forming potential of the telomeric repeats, was determined relative to the empty vector (*n*=2; error bars, SD). *P* values were calculated using an one-sample *t*-test. NS, $P > 0.05$. (D) Quantification of fragile telomeres on metaphase chromosome spreads prepared from a *Brca2*$^{-/-}$ mouse mammary tumor-derived cell line and BRCA2-reconstituted control cell line. Approximately 1000 telomeres were scored per sample (*n*=2; error bars, SD). *P* values were calculated using an unpaired two-tailed *t*-test. *, $P < 0.05$. Cell extracts were immunoblotted as indicated. SMC1 was used as a loading control. KB2P3.4, *Brca2*$^{-/-}$ mouse tumor-derived cell line; KB2P3.4+B2iBAC, *Brca2*$^{-/-}$ mouse tumor-derived cell line complemented with full-length BRCA2. (E) *p53*$^{-/-}$ MEFs grown for 48 h in the presence or absence of 5 μM PDS were arrested in mitosis with colcemid and mitotic chromosomes were spread using the cytospin method. Preparations were fixed and stained with an anti-γH2AX monoclonal antibody (green). Telomeres were visualized with a Cy3-conjugated $(CCCTAA)_6$-PNA probe (red), using identical exposure conditions for the two chromosome preparations. DNA was counter-stained with DAPI (blue).

Figure S2 (related to Figures 2 and 3)

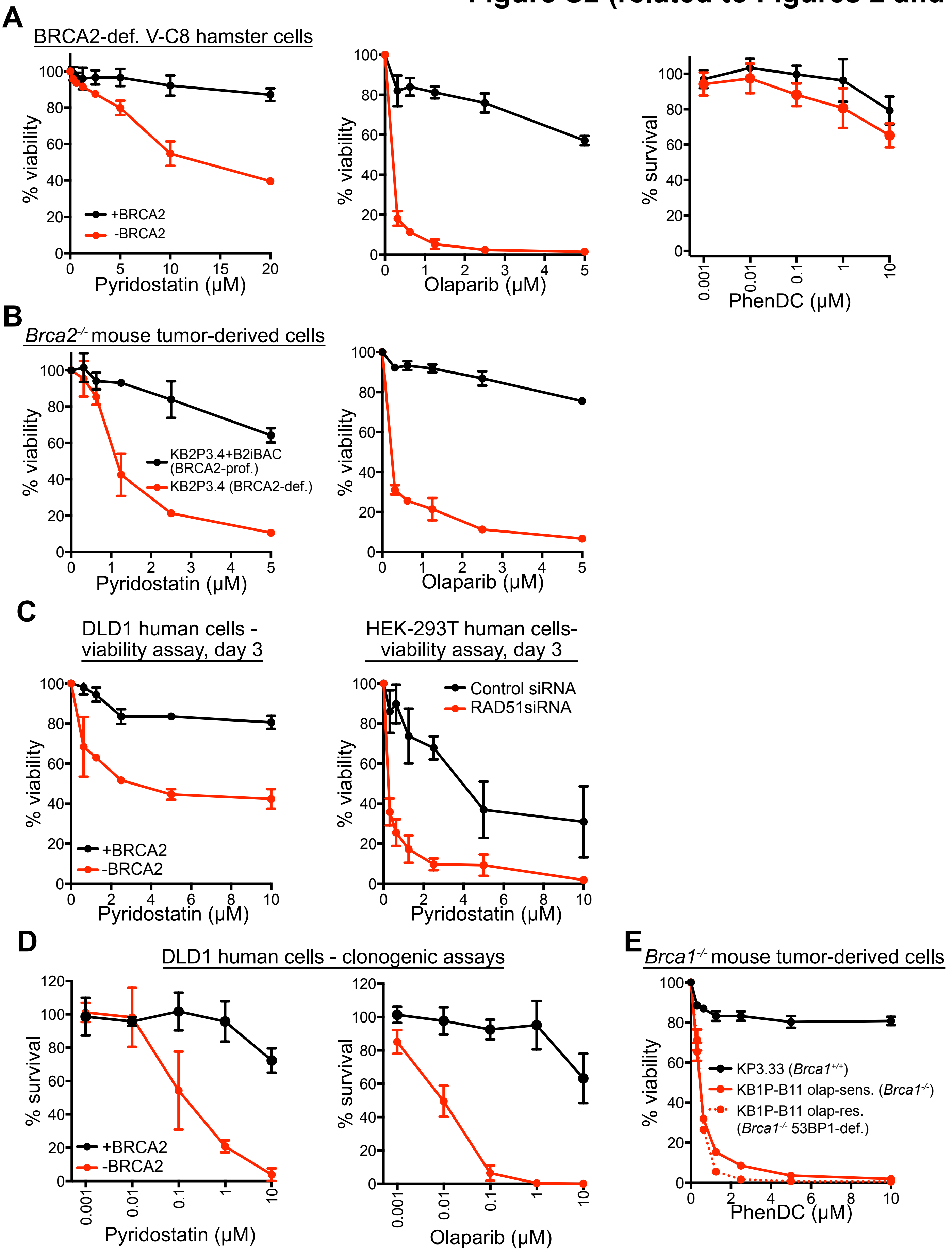

Figure S2 (related to Figures 2 and 3). (A) Dose-dependent viability and survival assays of BRCA2-deficient (-BRCA2) and -reconstituted (+BRCA2) hamster V-C8 cell lines. (B) Dose-dependent viability assays of *Brca2*$^{-/-}$ mouse mammary tumor-derived cell lines. (C) Dose-dependent viability assays of human cells treated with PDS for three days. Viability assays performed in the same cells after six days of treatment are shown in Figures 3A and 3C. (D) Clonogenic survival assays of human DLD1 cells treated with the indicated concentrations of PDS or olaparib for 24 h. Following removal of the drugs, cells were incubated in fresh media for 10-14 days before colony staining. (E) Dose-dependent viability assays of *Brca1*$^{-/-}$ mouse mammary tumor-derived cell lines. Graphs shown are representative of at least two independent experiments, each performed in triplicate. Error bars represent SD of triplicate values obtained from a single experiment.

Figure S3 (related to Figure 4)

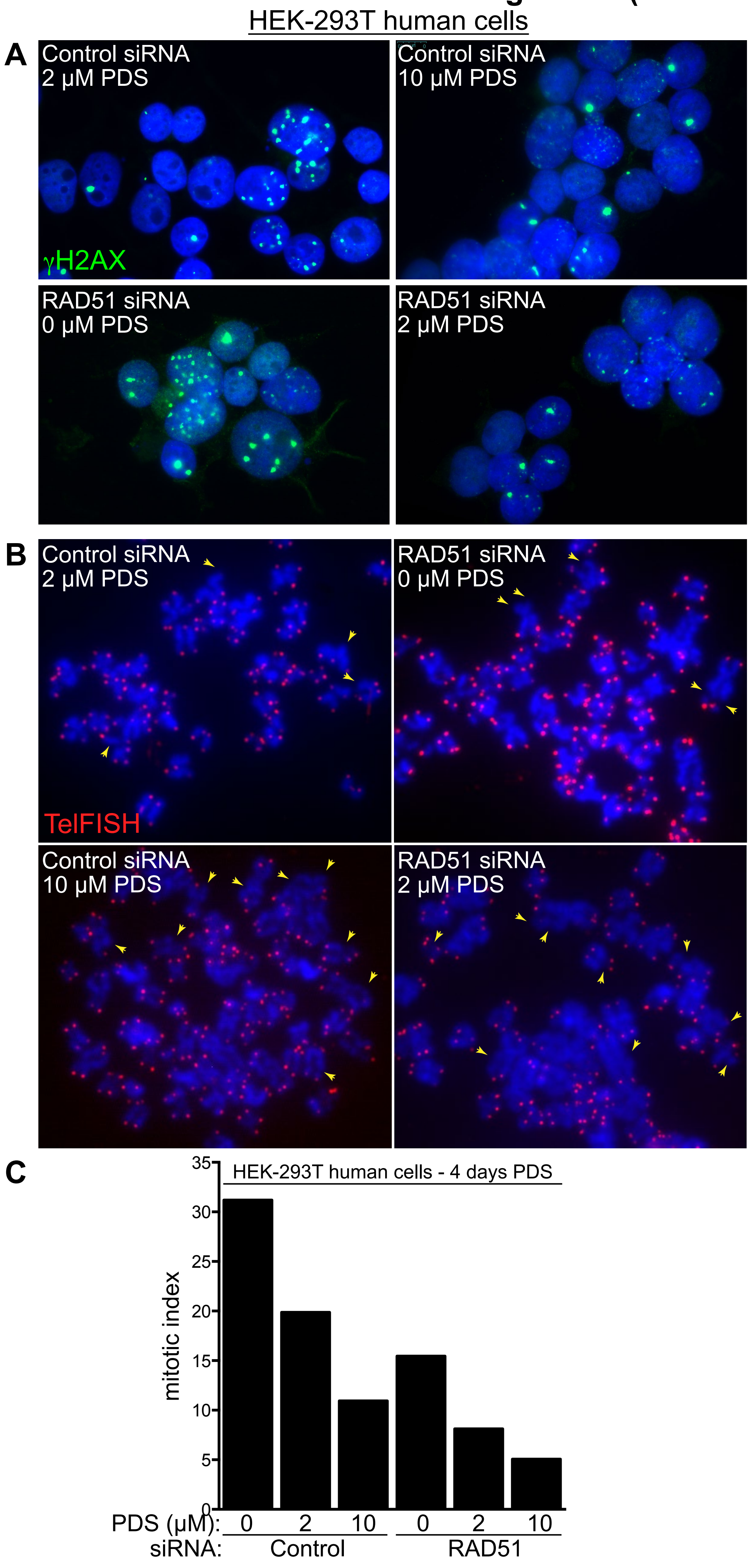

Figure S3 (related to Figure 4). (A and B) Additional representative images for the quantifications shown in Figures 4B (A) and 4D (B). (C) Human HEK-293T cells transfected with control or RAD51 siRNAs were treated with PDS for four days. Metaphase chromosome spreads were prepared by mitotic shake-off after colcemid treatment. DNA was stained with DAPI for quantification of mitotic nuclei. Mitotic index is expressed as % of total number of cells.

Figure S4 (related to Figure 5)

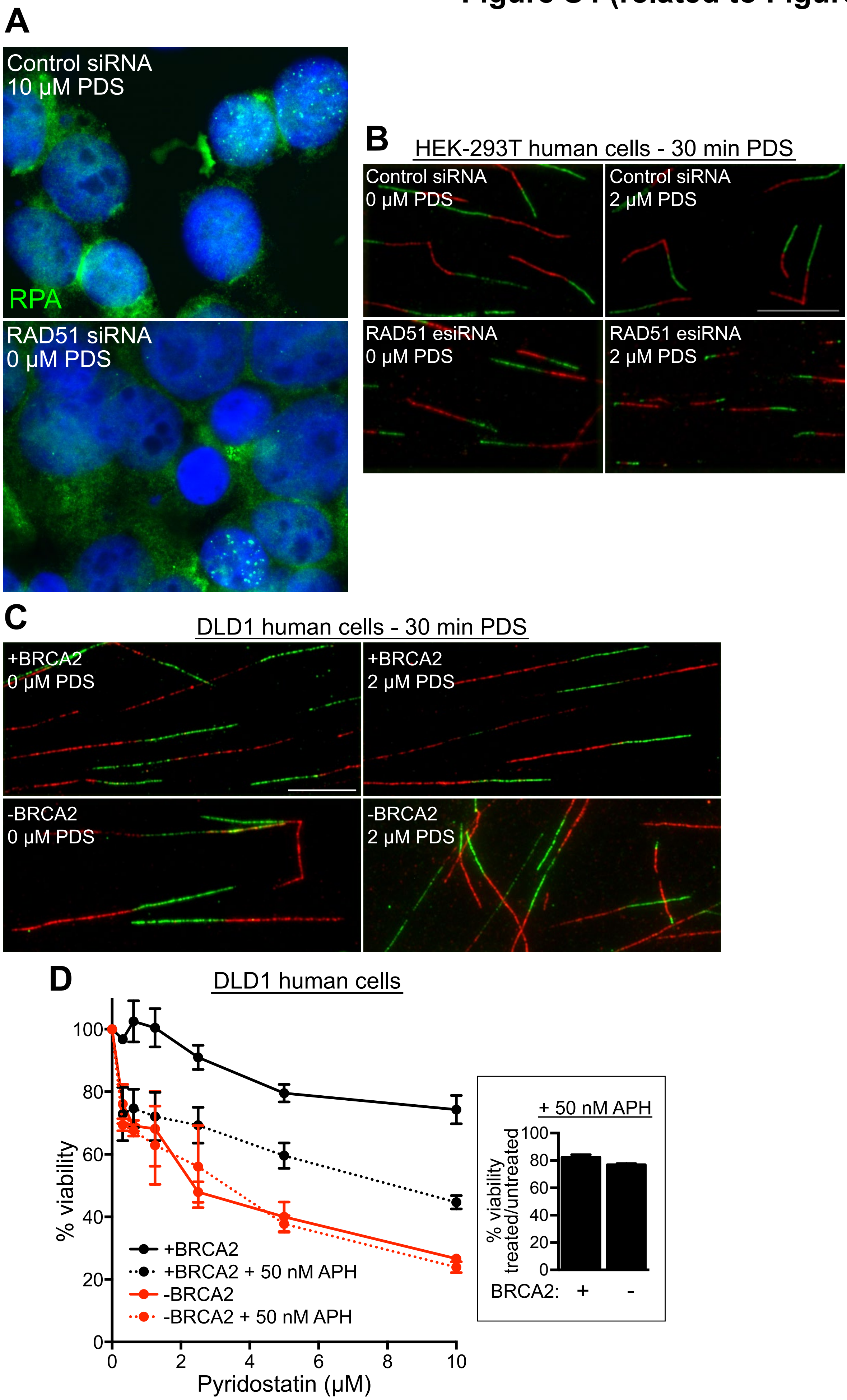

Figure S4 (related to Figure 5). (A) Additional representative images for the quantifications shown in Figure 5B. (B and C) Representative images for DNA fibers assays shown in Figures 5D (B) and 5F (C). Scale bar, 10 µm. (D) Dose-dependent viability assays of human BRCA2-proficient (+BRCA2) or -deficient (-BRCA2) DLD1 cells treated with PDS at the indicated concentrations in the presence or absence of 50 nM aphidicolin (APH). The effect of 50 nM APH alone is shown in the inset. Graphs shown are representative of three independent experiments, each performed in triplicate. Error bars represent SD of triplicate values obtained from a single experiment.

Figure S5 (related to Figure 6)

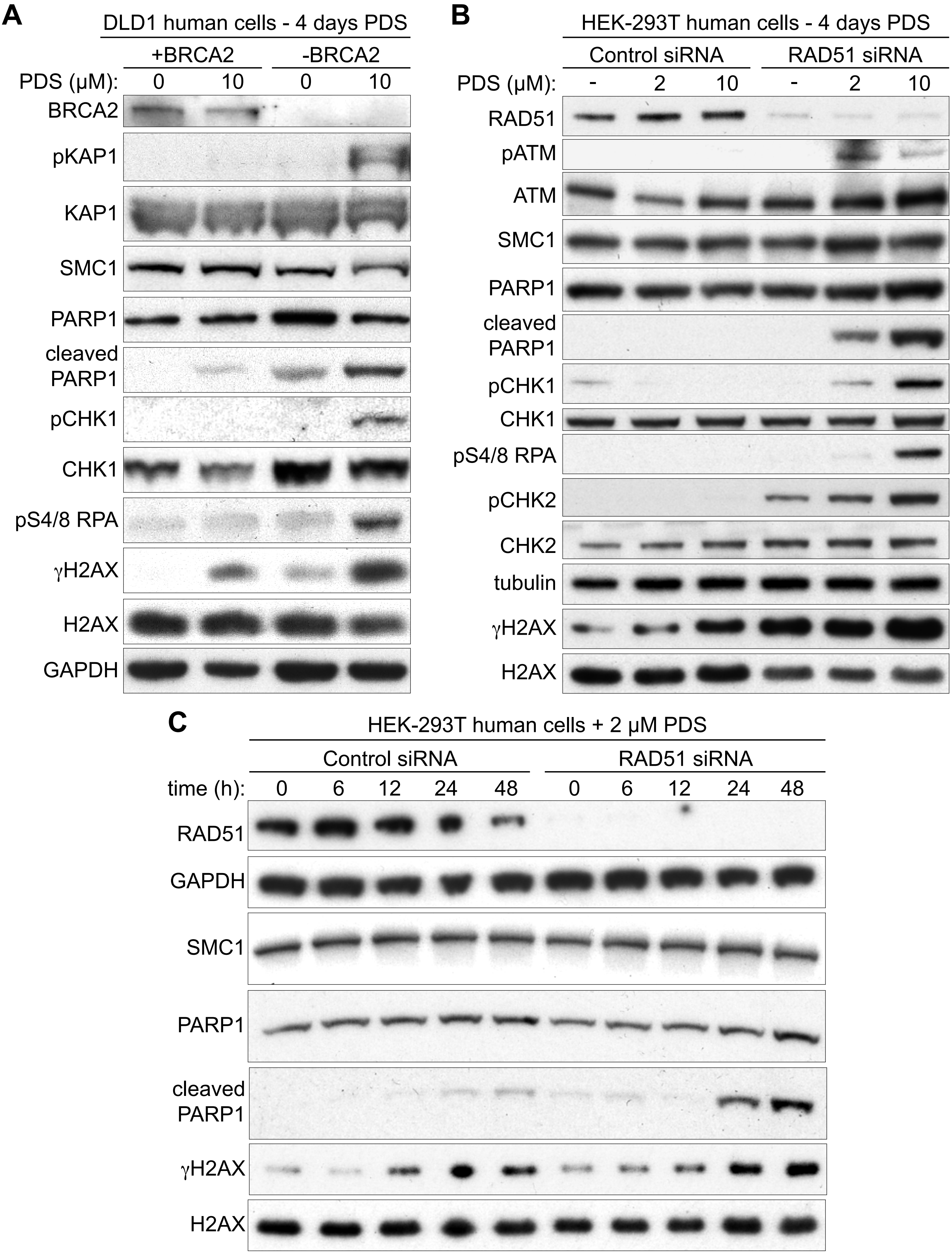

Figure S5 (related to Figure 6). (A) Cell extracts prepared from human DLD1 cells grown in the presence or absence PDS for four days were immunoblotted as indicated. SMC1 and GAPDH were used as loading controls. (B) Cell extracts of human HEK-293T human cells transfected with control or RAD51 siRNA were prepared after incubation with PDS for four days and immunoblotted as indicated. SMC1 and tubulin were used as loading controls. (C) Cell extracts of human HEK-293T cells transfected with control or RAD51 esiRNA were prepared at indicated time points after PDS addition and immuunoblotted as shown. SMC1 and H2AX were used as loading controls.

Figure S6 (related to Figure 6)

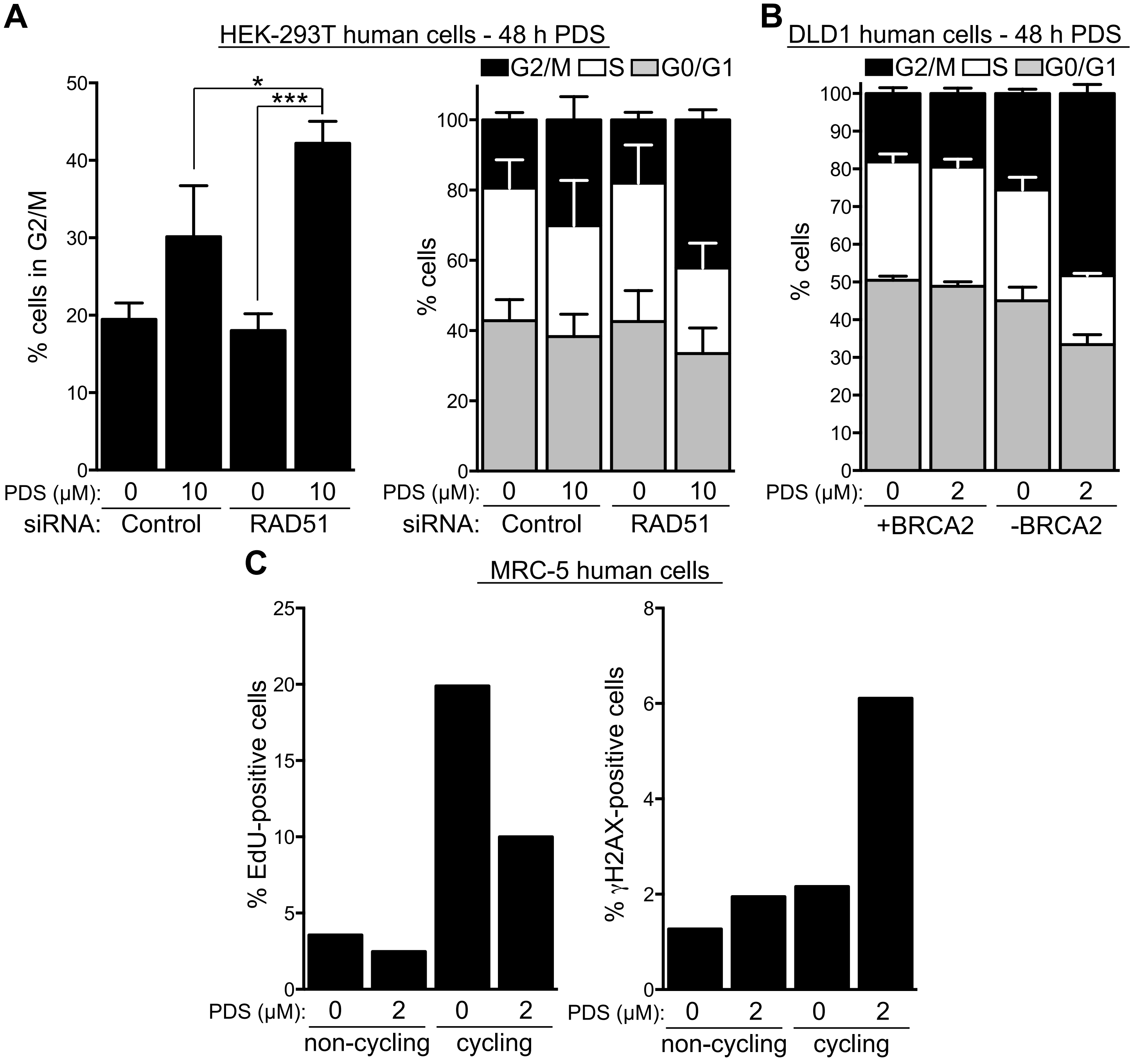

Figure S6 (related to Figure 6). (A) Cell cycle profiles of human HEK-293T cells transfected with control or RAD51 siRNA and treated with PDS for 48 h. Quantification of the percentage of cells in G2/M is also shown (*n*=3; error bars, SD). *P* values were calculated using an unpaired two-tailed *t*-test. *, $P \leq 0.05$; ***, $P \leq 0.001$. (B) Cell cycle profiles of human DLD1 cells, BRCA2-proficient (+BRCA2) and -deficient (-BRCA2), treated with PDS for 48 h (*n*=3; error bars, SD). Quantification of the percentage of cells in G2/M is shown in Figure 6B. (C) Human MRC-5 cells were arrested in G0 (non-cycling) by serum starvation or were allowed to re-enter the cell cycle (cycling) in the presence or absence of PDS. Replicating cells were labelled with EdU and DNA damage was detected with a FITC-conjugated γH2AX antibody. Cells were analysed by flow cytometry. Graphs are representative of two independent experiments.

Figure S7 (related to Figure 7)

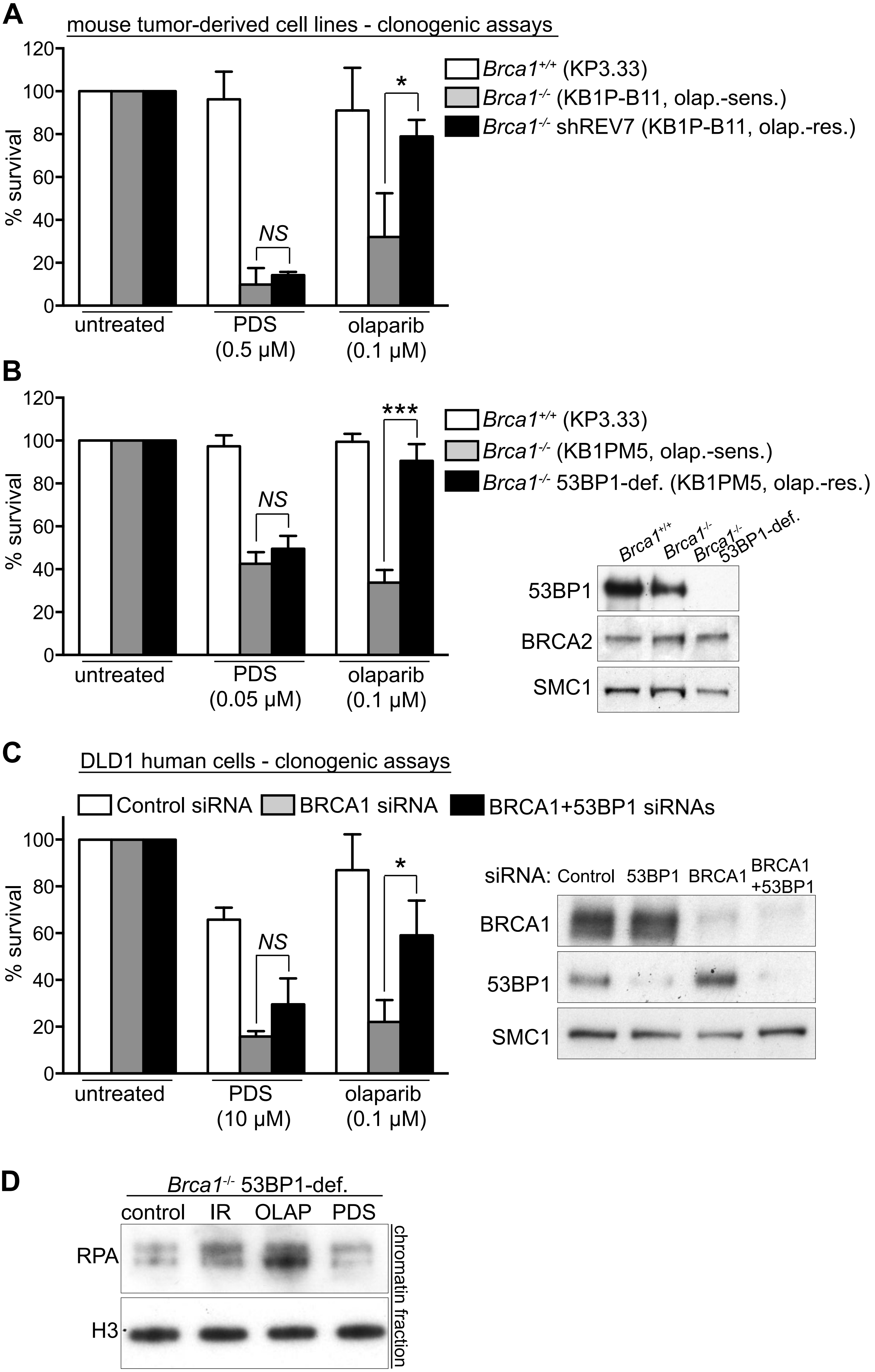

Figure S7 (related to Figure 7). (A and B) Colony survival assays of mouse mammary tumor-derived cell lines deficient in REV7 (A) or 53BP1 (B) . PDS or olaparib were added for 24 h. Graphs shown are representative of at least two independent experiments, each performed in triplicate. Error bars represent SD of triplicate values obtained from a single experiment. (B) Western blot analysis of cell extracts prepared from mouse mammary tumor-derived cell lines of indicated genotype. SMC1 was used as a loading control. (C) Human DLD1 cells were transfected with control, BRCA1 and/or 53BP1 siRNAs 24 h prior to seeding for colony survival assays performed as in (A). Graphs shown are representative of two independent experiments, each performed in triplicate. Error bars represent SD of triplicate values obtained from a single experiment. Cell extracts prepared 24 h after transfection were immunoblotted as indicated. SMC1 was used as a loading control. (D) Western blot analysis of chromatin-bound fractions of *Brca1*$^{-/-}$ 53BP1-deficient cells treated with 0.5 μM olaparib (OLAP) or PDS for 40 h, or irradiated with 10 Gy of ionizing radiation (IR), followed by recovery for 1 h. H3 was used as a loading control.

Supplemental Experimental Procedures

Cell lines and culture conditions

Human embryonic kidney HEK-293T cells, human non-small cell lung carcinoma H1299 cells and primary human fibroblasts MRC-5 (all from American Type Culture Collection), colorectal adenocarcinoma DLD1 cells (parental and *BRCA2*-mutated, Horizon Discovery; (Hucl et al., 2008)), as well as BRCA2-mutated hamster cells transduced with empty vector or BRCA2 (V-C8 and V-C8+BRCA2, respectively; (Kraakman-van der Zwet et al., 2002)) were cultivated in monolayers in DMEM medium (Sigma) supplemented with 10% fetal bovine serum (Life Technologies), penicillin and streptomycin (Sigma). H1299 cells expressing doxycycline (DOX)-inducible shRNAs were established using the 'all-in-one' system (Wiederschain et al., 2009). shRNAs targeting BRCA2 (GGG AAA CAC UCA GAU UAA A) or RAD51C (GAG AAU GUC UCA CAA AUA A) were cloned into $pLKO^{TetOn}$ and constructs were introduced into H1299 cells using lentiviral infection. Pooled cells showed efficient BRCA2 or RAD51C knockdown after eight days in the presence of 2 µg/ml DOX in DMEM medium supplemented with 10% tetracycline free fetal bovine serum (Clontech). A RAD51C shRNA-resistant H1299 cell line was generated by introducing the silent point mutations: 246T>C, 247C>T, 249C>A, 252A>T in a RAD51C-GFP expression construct in pEGFP-C1 vector. This construct and empty vector were introduced into H1299 cells expressing the DOX-inducible RAD51C shRNA using Lipofectamine™ 2000 (Life Technologies), following the manufacturer's protocol. To optimize the expression levels of RAD51C-GFP, single-cell clones were obtained by serial dilutions of the cell populations transfected with either RAD51C-GFP-expressing vector or empty vector control. GFP expression was assessed using an inverted microscope (DMI6000B; Leica) and a fluorescence imaging workstation.

$Brca1^{F/-}$, $Brca2^{F/-}$ and $Rad51c^{F/F}$ primary MEFs (Bouwman et al., 2010; Carlos et al., 2013; Kuznetsov et al., 2009) were isolated from day 13.5 embryos as previously described (Blasco et al., 1997), immortalized by overexpression of SV40 Large T (LT) antigen and cultivated in a low-oxygen (3%) incubator. For mitotic arrest, cells were treated with 0.2 µg/ml KaryoMAX® colcemid (Life Technologies) overnight. The following mouse mammary tumor-derived cell lines were used: $Brca1^{+/+}$ (KP3.33), $Brca1^{-/-}$ (KB1PM5, olaparib-sensitive) and $Brca1^{-/-}$ 53BP1-deficient (KB1PM5, olaparib-resistant; (Jaspers et al., 2013)); $Brca1^{-/-}$ (KB1P-B11, olaparib-sensitive), $Brca1^{-/-}$ shREV7 (KB1P-B11, olaparib-resistant; (Xu et al., 2015)); $Brca2^{-/-}$ (KB2P3.4, BRCA2-deficient), $Brca2^{-/-}$+B2iBAC (BRCA2-proficient; (Evers et al., 2010)). These cell lines were cultured at 37°C, 5% CO_2 and 3% O_2 in complete medium [DMEM/F-12, (Life Technologies) supplemented with 10% fetal bovine serum (Life Technologies), 1% penicillin-streptomycin (Sigma), 5 µg/ml insulin (Sigma), 5 ng/ml epidermal growth factor (Life Technologies), and 5 ng/ml cholera toxin (Sigma)].

MEF retroviral transduction

Retroviral transduction of cultured MEFs was performed as previously described (Palmero and Serrano, 2001). Briefly, HEK-293T packaging cells were grown to 70% confluency and transfected with pCL-Eco helper vector together with either pBabe alone, pBabe plus retroviral vector encoding 'Hit-and-run' Cre recombinase (Silver and Livingston, 2001) or shRNA against 53BP1 (Bouwman et al., 2010) using a standard calcium phosphate protocol. The medium was replaced 24 h after transfection. Recipient MEFs were plated and infected 24 h later with the retroviral supernatants produced by the HEK-293T cells. Additional infections were performed after 24 and 32 h. Twenty-four hours after the last infection, cells were incubated in fresh medium containing 3 µg/ml puromycin for 48 h.

***In vivo* experiment**

CD-1 male nude (nu/nu) mice, 6 weeks old and weighing 26-28 g were purchased from Charles River Laboratories (Calco, Italy). All animal procedures were in compliance with the national and international directives (D.L. March 4, 2014, no. 26; directive 2010/63/EU of the European Parliament and of the council; Guide for the Care and Use of Laboratory Animals, United States National Research Council, 2011). Mice were injected intramuscularly into the hind leg muscles with $5x10^6$ DLD1 BRCA2-proficient or -deficient cells per mouse. When a tumor mass of about 250 mg was evident in BRCA2-proficient (three days after cell injection) and -deficient (six days after cell injection) xenografts, the treatment was initiated. RHPS4 (10 mg/kg) was administered intravenously for ten consecutive days. Tumors were measured three times a week in two dimensions by a caliper and tumor weight was calculated using the formula $a \times b^2/2$, where a and b are the long and short sizes of the tumor, respectively. Each experimental group included eight mice. Therapeutic efficacy of treatment

was assessed by percent tumor weight inhibition (TWI%) calculated as [1-(mean tumor weight of treated mice/ mean tumor weight of controls)]x 100).

Plasmid-based replication assay

The construct containing the telomere sequence $(TTAGGG)_7$ used in Figures 1 and S1B was generated as in Szüts et al. (2008). A plasmid containing the mutant $(TTACGC)_7$ sequence, which abrogates the G4-forming potential of the telomeric sequence (Figure S1C), was made using QuikChange II XL Site-Directed Mutagenesis Kit (Agilent Technologies) following the manufacturer's protocol. H1299 cells expressing DOX-inducible BRCA2 or RAD51C shRNAs were grown for eight days in the presence or absence of DOX and plated at 90% confluency. The following day, Lipofectamine™ 2000 (Life Technologies) was used to co-transfect 10 µg of $pQ1^{Amp}$ plasmid containing $(TTAGGG)_7$ or empty vector together with control $pQ2^{Kan}$ plasmid. Plasmid DNA was extracted 48 h after transfection using a simplified Hirt protocol. Briefly, cells were harvested by trypsinization, washed in PBS, resuspended in buffer P1 from a plasmid miniprep kit (Qiagen), lysed in buffer P2 and neutralized in buffer N3. Plasmid DNA was recovered from the resulting supernatant using glycogen and isopropanol precipitation. Dried pellets were dissolved in DpnI digest mix (containing 10 U DpnI) and incubated at 37°C for 30 min to degrade parental DNA. Plasmid DNA was recovered using isopropanol precipitation, dried and dissolved in 5 µl dH_2O. Recovered DNA was used to electroporate 20 µl E-shot electro-competent cells (Life Technologies) at 200 Ω, 0.25 µF and 1.8 kV in a Bio-Rad E. coli pulser™ (165-2104) and 500 µl SOC was added immediately. Cells were allowed to recover on ice for 5 min and subsequently incubated at 37°C for 1 h. Typically 200 µl cells were plated on kanamycin and 20 µl on ampicillin plates. Colony numbers were quantified and plasmid replication efficiency (%) was determined by normalizing the Amp^R colonies to the internal Kan^R control, followed by expressing the replication efficiency of $pQ1^{Amp}$ plasmid containing $(TTAGGG)_7$ relative to the replication efficiency of the empty vector.

RNAi

1.5×10^6 cells were transfected with 40 nM siRNA per plate by reverse transfection in 10-cm plates. After 24-h incubation, depletion was evident as determined by immunoblotting. When using RAD51 siRNA, cells were transfected again three and six days after the first transfection as above. The 53BP1 siRNA CAG GAC AGT CTT TCC ACG AAT, BRCA1 siRNA CAG CAG TTT ATT ACT CAC TAA and RAD51 siRNA CUU UGG CCC ACA ACC CAU with two-base deoxynucleotide overhangs were obtained from Dharmacon, RAD51 esiRNA from Sigma and AllStars negative control siRNA from Qiagen.

Clonogenic assays

Cells were plated at densities between 100 and 1,000 cells per well in 6-well plates and drug treatment was initiated after cells had adhered. Following 24-h incubation with the drug, fresh media without the drug were added for 6-14 days. Colonies were stained with 0.5% crystal violet (Sigma) in 50% methanol, 20% ethanol in dH_2O. Cell survival was expressed relative to untreated cells of the same cell line, thus accounting for any differences in viability caused by HR deficiency.

Proliferation assays

To determine population doublings, resazurin-based readouts of cell viability were taken after cells had adhered (day 0) and every 24 h for four days.

MRC-5 cell cycle arrest

MRC-5 cells were cultured in media containing 0.1% fetal bovine serum for four days to induce G0 arrest. To enable re-entry into the cell cycle, 10% fetal bovine serum was added to the media for 48 h.

EdU incorporation and gH2AX labeling

To label replicated DNA, cells were incubated with 10 µM EdU for 45 min and incorporated EdU was detected using the Click-iT EdU Alexa Fluor 647 Flow Cytometry Assay Kit (Molecular Probes) according to manufacturer's instructions. To stain for γH2AX, cells were incubated with an anti-γH2AX (Ser139) FITC conjugate (1:50, Millipore) for 1 h at room temperature. Cells were resuspended in PBS containing 20 µg/ml propidium iodide and 10 µg/ml RNase A before samples were processed using flow cytometry (BD FACSCalibur, BD Biosciences) and data was analyzed using FlowJo software.

Alkaline single-cell gel electrophoresis comet assay

2 x 10^5 cells were embedded in 1% low-melting agarose in PBS on a microscope slide. Subsequently, the cells were lysed in buffer containing 2.5 M NaCl, 100 mM EDTA, 10 mM Tris-HCl pH 10.5, 1% DMSO and 1% Triton X-100 for 1 h at 4°C. To denature the DNA, the slides were incubated in cold electrophoresis buffer (300 mM NaOH, 1 mM EDTA, 1% DMSO, pH >13) for 30 min in the dark. Following electrophoresis at 25 V and 300 mA for 25 min, the DNA was neutralized with 0.5 M Tris-HCl pH 8.0 and stained with SYBR Gold (Invitrogen).

Immunofluorescence (IF)

Cells were washed in PBS, swollen in hypotonic solution (85.5 mM NaCl and 5 mM $MgCl_2$) for 5 min, fixed with 4% paraformaldehyde for 10 min at room temperature (and with 100% ice-cold methanol for RPA) and permeabilized by adding 0.03% SDS to the fixative. After blocking with blocking buffer (1% goat serum, 0.3% BSA, 0.005% Triton X-100 in PBS), cells were incubated with primary antibody diluted in blocking buffer overnight at room temperature. Then, they were washed again and incubated with fluorochrome-conjugated secondary antibodies for 1 h at room temperature. Dried coverslips were mounted on microscope slides using the ProLong Antifade kit (Life Technologies) supplemented with 2 μg/ml DAPI.

Preparation of metaphase spreads

Mitotic cells were collected by mitotic shake-off and swollen in hypotonic buffer (0.03 M sodium acetate) at 37°C for 25 min. For telomere FISH, cells were fixed in a freshly prepared 3:1 mix of methanol:glacial acetic acid. Nuclear preparations were dropped onto slides pre-soaked in 45% acetic acid and left to dry overnight.

Telomeric FISH

Telomeric probe mix containing: 10 mM Tris pH 7.5, 2.175 mM $MgCl_2$, 0.08 mM citric acid, 7.2 mM Na_2HPO_4 pH7.0, 70% deionized formamide (Chemicon Int.), 0.5 μg/ml Cy3-conjugated PNA $(CCCTAA)_3$ telomeric probe (Applied Biosystems) and 0.25% blocking reagent (100 mM maleic acid and 50 mM NaCl pH7.5 (Roche) in dH_2O) was dropped onto each slide and sealed with a coverslip. Following denaturation on a hot plate for 3 min at 80°C, the slides were incubated at room temperature for 1.5 h in a dark humidified chamber. After washing twice in formamide (70% formamide (Fluka), 10 mM Tris, 0.1% BSA (Fluka)), three times in PBS and once in dH_2O, the slides were left to dry at room temperature. Slides were then mounted using ProLong Antifade (Life Technologies) supplemented with 2 μg/ml 4,6-diamidino-2-phenylindole (DAPI). Mitotic chromosomes were viewed with a Leica DMI6000B inverted microscope and fluorescence imaging workstation equipped with a HCX PL APO 100x/1.4-0.7 oil objective. Images were acquired using a Leica DFC350 FX R2 digital camera and LAS-AF software (Leica). Brightness levels and contrast adjustments were applied to the whole image using Photoshop CS3 (Adobe).

Telomere chromosome orientation FISH (CO-FISH)

After washing in PBS for 10 min, the slides were treated with 0.5 mg/ mL RNase A (Sigma Aldrich) in PBS for 10 min at 37°C. Following washes in PBS and 2 x saline-sodium citrate (SSC), the DNA was stained with 0.5 mg/ml Hoechst 33258 (Sigma Aldrich) in 2xSSC for 15 min. In order to introduce DNA breaks, slides were exposed to ultraviolet (UV; 365 nm) light for 25 min. Following PBS washes, the DNA was digested with 3 U/mL of Exonuclease III (Promega). The slides were washed again with PBS and then dehydrated with sequential washes of 70, 90 and 100% ethanol. When dried, the first telomeric probe mix (same as described for IF-FISH) containing 0.5 μg/ml Cy3-conjugated PNA $(TTAGGG)_3$ telomeric probe (Applied Biosystems) was added to the slides. DNA was denatured on a hot plate at 80°C for 3 min, then slides were incubated for 1.5 h at room temperature and washed twice with formamide wash, as described for IF-FISH. Next the slides were washed with PBS, dehydrated with ethanol and after air drying, were incubated with the second telomeric probe mix containing 0.5 μg/ml FITC-conjugated PNA $(CCCTAA)_3$ telomeric probe (Applied Biosystems) for 1.5 h at room temperature. Following washes with formamide wash and PBS and dehydration with ethanol, slides were dried and mounted using Vectashield (Vector Laboratories) supplemented with 2 μg/ml DAPI.

DNA fiber assay

DNA was labeled with 25 μM CldU and 250 μM IdU for 30 min each. The reaction was terminated by addition of ice-cold PBS. After cell lysis, DNA was spread on glass slides, fixed in methanol/acetic acid, denatured with HCl, blocked with 2% BSA and stained with anti-rat anti-CldU (1:500, Abcam) and mouse anti-IdU (1:100, Beckton Dickinson) antibodies. Anti-rat Cy3 (1:300, Jackson

ImmunoResearch) and anti-mouse Alexa 488 (1:300, Molecular Probes) were used as secondary antibodies. Images were acquired as described for FISH and analyzed using ImageJ software (National Healthcare Institute, USA).

Immunoblotting and cell fractionation
Cells were harvested by trypsinization, washed with cold PBS, re-suspended in SDS-PAGE loading buffer, sonicated and boiled at 70°C for 10 min to prepare whole cell extracts. Fractionation of human cells was performed as described in Rodrigue et al. (2006). Chromatin fractions of mouse cells were prepared as described in Mendez and Stillman (2000). Equal amounts of protein (50-100 µg) were analysed by gel electrophoresis followed by Western blotting. NuPAGE-Novex 10% Bis-Tris and 3-8% Tris-Acetate gels (Life Technologies) were run according to manufacturer's instructions.

Antibodies
The following antibodies were used for immunoblotting: rabbit polyclonal antisera raised against 53BP1 (NB100-304, Novus), phosphorylated ATM Ser1981 (10H11, Cell Signaling), ATM (MAT3-4G10/8, Sigma-Aldrich), phosphorylated CHK1 Ser317&Ser345 (2344&2341, Cell Signaling), phosphorylated CHK2 Thr68 (Cell Signaling), H2AX (DR1016, Calbiochem), H3 (H3C, a gift from A. Verreault, University of Montreal), phosphorylated KAP1 Ser824 (A300-767A, Bethyl Laboratories), KAP1 (A300-274A, Bethyl Laboratories), cleaved PARP1 Asp214 (9541, Cell Signaling), PARP1 (46D11, Cell Signaling), RAD51 (H92, Santa Cruz), phosphorylated RPA32 Ser4&Ser8 (A300-245A, Bethyl Laboratories), SMC1 (BL308, Bethyl Laboratories), mouse monoclonal antibodies raised against RAD51C (2H11, Cancer Research UK Monoclonal Antibody Service), BRCA2 (OP95, Calbiochem), CHK1 (sc-8408, Santa Cruz), CHK2 (clone 7, Merck Millipore), GAPDH (6C5, Novus Biologicals), α-tubulin (TAT1, Cancer Research UK Monoclonal Antibody Service), phosphorylated histone H2AX Ser139 (clone JBW301, Merck Millipore), a rat monoclonal antibody raised against RPA2 (a gift from H. P. Nasheuer, NUI Galway) and a sheep polyclonal antibody raised against mouse BRCA2 ((Min et al., 2012); a gift from H. Lee, Seoul National University). Additional antibodies used for immunofluorescence detection were: rabbit polyclonal antiserum raised against RAD51 (H92, Santa Cruz), rabbit polyclonal antiserum raised against RPA (SWE34, a gift from Steve West, Cancer Research UK Clare Hall Laboratories) and mouse monoclonal antibody raised against phosphorylated histone H2AX Ser139 (clone JBW301, Merck Millipore).

References

Blasco, M.A., Lee, H.W., Hande, P.M., Samper, E., Lansdorp, P.M., DePinho, R.A., and Greider, C.W. (1997). Telomere shortening and tumor formation by mouse cells lacking telomerase RNA. Cell *91*, 25-34.

Bouwman, P., Aly, A., Escandell, J.M., Pieterse, M., Bartkova, J., van der Gulden, H., Hiddingh, S., Thanasoula, M., Kulkarni, A., Yang, Q*., et al.* (2010). 53BP1 loss rescues BRCA1 deficiency and is associated with triple-negative and BRCA-mutated breast cancers. Nat Struct Mol Biol *17*, 688-695.

Carlos, A.R., Escandell, J.M., Kotsantis, P., Suwaki, N., Bouwman, P., Badie, S., Folio, C., Benitez, J., Gomez-Lopez, G., Pisano, D.G*., et al.* (2013). ARF triggers senescence in Brca2-deficient cells by altering the spectrum of p53 transcriptional targets. Nature communications *4*, 2697.

Evers, B., Schut, E., van der Burg, E., Braumuller, T.M., Egan, D.A., Holstege, H., Edser, P., Adams, D.J., Wade-Martins, R., Bouwman, P*., et al.* (2010). A high-throughput pharmaceutical screen identifies compounds with specific toxicity against BRCA2-deficient tumors. Clinical cancer research : an official journal of the American Association for Cancer Research *16*, 99-108.

Hucl, T., Rago, C., Gallmeier, E., Brody, J.R., Gorospe, M., and Kern, S.E. (2008). A syngeneic variance library for functional annotation of human variation: application to BRCA2. Cancer Res *68*, 5023-5030.

Jaspers, J.E., Kersbergen, A., Boon, U., Sol, W., van Deemter, L., Zander, S.A., Drost, R., Wientjens, E., Ji, J., Aly, A*., et al.* (2013). Loss of 53BP1 causes PARP inhibitor resistance in Brca1-mutated mouse mammary tumors. Cancer discovery *3*, 68-81.

Kraakman-van der Zwet, M., Overkamp, W.J.I., van Lange, R.E.E., Essers, J., van Duijn-Goedhart, A., Wiggers, I., Swaminathan, S., van Buul, P.P.W., Errami, A., Tan, R.T.L*., et al.* (2002). BRCA2 (XRCC11) deficiency results in radioresistant DNA synthesis and a higher frequency of spontaneous deletions. Mol. Cell Biol. *22*, 669-679.

Kuznetsov, S., Haines, D.C., Martin, B.K., and Sharan, S.K. (2009). Loss of RAD51C leads to embryonic lethality and modulation of Trp53-dependent tumorigenesis in mice. Cancer Res. *69*, 863-872.

Mendez, J., and Stillman, B. (2000). Chromatin association of human origin recognition complex, cdc6, and minichromosome maintenance proteins during th ecell cycle: assembly of prereplication complexes in late mitosis. Mol. Cell Biol. *20*, 8602-8612.

Min, J., Choi, E.S., Hwang, K., Kim, J., Sampath, S., Venkitaraman, A.R., and Lee, H. (2012). The breast cancer susceptibility gene BRCA2 is required for the maintenance of telomere homeostasis. J. Biol. Chem. *287*, 5091-5101.

Palmero, I., and Serrano, M. (2001). Induction of senescence by oncogenic Ras Methods in Enzymology *333*, 247-256.

Rodrigue, A., Lafrance, M., Gauthier, M.-C., McDonald, D., Hendzel, M., West, S.C., Jasin, M., and Masson, J.-Y. (2006). Interplay between human DNA repair proteins at a unique double-strand break in vivo The EMBO journal *25*, 222-231.

Silver, D.P., and Livingston, D.M. (2001). Self-excising retroviral vectors encoding the Cre recombinase overcome Cre-mediated cellular toxicity. Mol. Cell *8*, 233-243.

Szüts, D., Marcus, A.P., Himoto, M., Iwai, S., and Sale, J.E. (2008). REV1 restrains DNA polymerase zeta to ensure frame fidelity during translesion synthesis of UV photoproducts in vivo. Nucleic Acids Res. *36*, 6767-6780.

Wiederschain, D., Wee, S., Chen, L., Loo, A., Yang, G., Huang, A., Chen, Y., Caponigro, G., Yao, Y.M., Lengauer, C., *et al.* (2009). Single-vector inducible lentiviral RNAi system for oncology target validation. Cell Cycle *8*, 498-504.

Xu, G., Chapman, J.R., Brandsma, I., Yuan, J., Mistrik, M., Bouwman, P., Bartkova, J., Gogola, E., Warmerdam, D., Barazas, M., *et al.* (2015). REV7 counteracts DNA double-strand break resection and affects PARP inhibition. Nature *521*, 541-544.
